# Sampling and ranking spatial transcriptomics data embeddings to identify tissue architecture

**DOI:** 10.3389/fgene.2022.912813

**Published:** 2022-08-12

**Authors:** Yu Lin, Yan Wang, Yanchun Liang, Yang Yu, Jingyi Li, Qin Ma, Fei He, Dong Xu

**Affiliations:** ^1^ School of Artificial Intelligence, Jilin University, Changchun, China; ^2^ Department of Electrical Engineering and Computer Science and Christopher S. Bond Life Sciences Center, University of Missouri, Columbia, MO, United States; ^3^ Key Laboratory of Symbol Computation and Knowledge Engineering of Ministry of Education, College of Computer Science and Technology, Jilin University, Changchun, China; ^4^ School of Computer Science, Zhuhai College of Science and Technology, Zhuhai, China; ^5^ School of Information Science and Technology, Northeast Normal University, Changchun, China; ^6^ Department of Biomedical Informatics, The Ohio State University, Columbus, OH, United States

**Keywords:** embedding evaluation, deep learning, message passing, spatial transcriptomics, tissue architecture, spatial autocorrelation

## Abstract

Spatial transcriptomics is an emerging technology widely applied to the analyses of tissue architecture and corresponding biological functions. Substantial computational methods have been developed for analyzing spatial transcriptomics data. These methods generate embeddings from gene expression and spatial locations for spot clustering or tissue architecture segmentation. Although the hyperparameters used to produce an embedding can be tuned for a given training set, a fixed embedding has variable performance from case to case due to data distributions. Therefore, selecting an effective embedding for new data in advance would be useful. For this purpose, we developed an embedding evaluation method named message passing-Moran’s I with maximum filtering (MP-MIM), which combines message passing-based embedding transformation with spatial autocorrelation analysis. We applied a graph convolution to aggregate spatial transcriptomics data and employed global Moran’s I to measure spatial autocorrelation and select the most effective embedding to infer tissue architecture. Sixteen spatial transcriptomics samples generated from the human brain were used to validate our method. The results show that MP-MIM can accurately identify high-quality embeddings that produce a high correlation between the predicted tissue architecture and the ground truth. Overall, our study provides a novel method to select embeddings for new test data and enhance the usability of deep learning tools for spatial transcriptome analyses.

## Introduction

Spatial transcriptomics is a method for profiling the transcriptome of tissues within the spatial context. The method combines spatial information and tissue morphology with the transcriptional profiles of individual cells/spots in the tissue. This novel method is gaining ground through the 10x Genomics Visium (Stahl et al., 2016; Asp et al., 2019; Burgess, 2019) to characterize tissue architecture, which provides a basis for studying spatial heterogeneity and the underlying biological process. This research direction includes discovering tissue architecture, inferring cell types, detecting spatially variable genes, and explaining biological mechanisms by using spatial transcriptomics data (Svensson et al., 2018; Queen et al., 2019).

Several methods have been proposed for spatial transcriptome analysis. SpaGCN (Hu et al., 2021) and stLearn (Pham et al., 2020) are deep learning models used to identify tissue architecture. BayesSpace (Zhao et al., 2020) employs a Bayesian approach for spot clustering analysis by imposing prior knowledge. Giotto (Dries et al., 2019) applied graph-based clustering methods for characterizing tissue spatial organization and data visualization. RESEPT (Chang et al., 2021) provides a deep learning framework for characterizing and visualizing the tissue architecture by reconstructing and segmenting a transcriptome-mapped RGB (red, green, and blue) image. These tools can be used to analyze and visualize many spatial transcriptomics data for developing new hypotheses, such as linking tissue architecture with biological functions. These methods typically use generative embeddings for performing different tasks of downstream analyses. Rich features are learned and converted into embedding values by deep learning models using both gene expressions and spatial location information.

Among these spatial transcriptome analyses, graph neural networks (GNNs) (Wu et al., 2020; Zhang et al., 2020; Zhou et al., 2020) have been widely employed recently (Chang et al., 2021; Hu et al., 2021). GNNs learn graph embeddings in spatial transcriptomics data with spatial location information. The efficient embeddings are generated by neighborhood information aggregation involving pushing messages from surrounding spots around a given reference spot (Cai et al., 2018). Several spatial transcriptome analysis tools, such as SpaGCN and RESEPT, use a popular type of GNN, the graph convolutional network (GCN) (Kipf and Welling, 2016), in their workflows to learn effective embeddings for the downstream analyses of clustering and image segmentation. For these tools, it is possible to adjust hyperparameters to produce better embedding to improve model performance. This study focuses on evaluating embeddings sampled from a deep learning model with different hyperparameter settings and selecting high-quality embeddings to enhance the performance of a spatial transcriptome analysis model. For this purpose, we adapt spatial autocorrelation, which describes the correlation among values of a single variable based on their proximity in space to reflect a pattern of attribute values that is nonrandom over a set of spatial units (Griffith, 1987). Moran’s I and Geary’s C (Moran, 1950; Geary, 1954) are two leading measuring indices used to detect spatial relevance. In addition, the Getis-Ord G index (Getis and Ord, 2010) measures the degree of clustering for hot or cold spots. Spatial autocorrelation is widely used in various research fields, such as georeferenced data (Griffith and Chun, 2016), remote sensing for information extraction (Curran, 1988; Warner and Shank, 1997), landslide susceptibility (Lee, 2005), and hazard modeling (Pardeshi et al., 2013). Spatial transcriptome analysis, such as SpaGCN, employs Moran’s I to detect spatially variable genes.

This study presents a novel embedding evaluation method named MP-MIM (message passing-Moran’s I with maximum filtering), which employs message passing and the spatial autocorrelation index to evaluate embeddings from deep learning models used for spatial transcriptome analysis. The message passing part generates a more accurate and representative embedding. The global Moran’s I index is applied to indicate the spatial correlation intensity to measure the quality of each dimension, and the maximum value of Moran’s I is used to select the most effective embedding. We used 16 spatial transcriptomics samples (Chen et al., 2021; Maynard et al., 2021) for our study. According to the test results, our proposed method accurately identified high-quality embeddings, thereby enhancing the tissue architecture identification of spatial analysis models.

## Materials and methods

**FIGURE 1:**
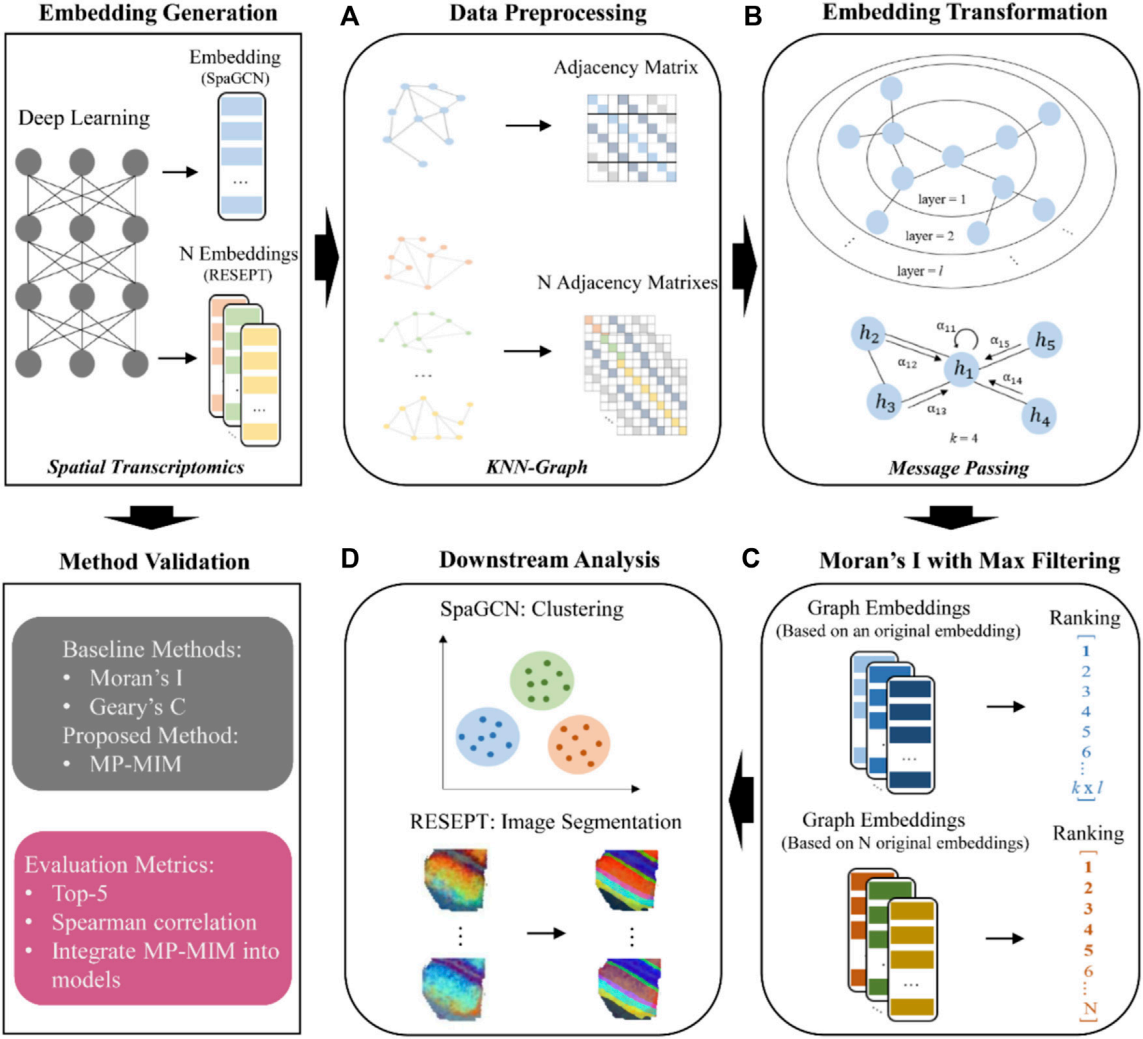
Workflow of the proposed method MP-MIM and method validation. MP-MIM takes the embedding generated from the deep learning model by using spatial transcriptomics data as the input. **(A)**, **(B)**, **(C),** and **(D)** show the process starting from converting the input into the KNN graph. Message passing based on graph convolution transforms the original embedding with the KNN-graph structure to graph embedding. Moran’s I evaluated the multiple transformed embeddings with maximum filtering according to all the dimensions of each embedding. Finally, the graph embeddings and quality ranking are the output for the downstream analysis in their respective models. The method validation panel shows the baseline comparison methods and the specific evaluation metrics.

The main workflow of MP-MIM is aimed at assessing the embeddings generated from deep learning models with different hyperparameters on spatial transcriptomics data and identifying high-quality embeddings. As shown in Figure 1, the embedding generation panel illustrates the process of embedding sampling by deep learning models. The overall workflow of integrating MP-MIM into spatial models includes four parts: (A) data preprocessing to convert the input embedding into graph-structured data, (B) embedding transformation by message passing, (C) embedding evaluation by using Moran’s I with maximum filtering, and (D) the graph embeddings and ranking used for the downstream analysis of the respective models. To validate the proposed method, MP-MIM, the panel of method validation also details the baseline comparison methods and evaluation metrics used in this study.

### Conversion of input data into graph-structured data

Figure 1A shows that our method takes each embedding generated from spatial transcriptomics data as the input. Each spot that contains one or more cells is used as a node unit in our *k*-nearest neighbor (KNN) graph. We first convert the embedding of cell spot features (gene expression) into an undirected graph employing the KNN algorithm (Hastie and Tibshirani, 1996) in the graph. In the KNN graph, the preset parameter *k* is used to decide the degree of interaction between each node. Each node creates edges to its neighbors with the *k*-nearest Euclidian distances. To maintain a more efficient graph structure, we further search for an adaptive number of neighbors for each node by pruning the graph (Wang et al., 2021), where the outliers in *k* neighbors of each node are deleted. We can generate the final adjacency matrix based on the aforementioned KNN graph for the next step of MP-MIM.

### Embedding transformation

We employ the message-passing framework used in a popular deep learning model, GAT (Velickovic et al., 2017), to transform embedding layer by layer. The input is a set of node features from each sample; this set is denoted as h = $\left\{h_{1},h_{2},\ldots ,h_{N}\right\}$ , where *N* represents the number of nodes. The message passing process for each node in embedding with the graph structure is shown in Figure 1B, where every node aggregates features from its neighborhood nodes with different weights. We consider that long-distance dependency in spatial space can also contribute to tissue architecture identification, but existing GNN-based methods usually pay more attention to short-distance dependency. To generate an enhanced and complementary embedding for ranking embeddings, we define the initial weight between two nodes by using the Euclidian distance as follows:

$$e_{ij}=distance\left(h_{i},h_{j}\right).$$ (1)

Then, the softmax function is used to make the weight easily comparable among different nodes:

$$\alpha_{ij}=\;softmax_{j}\left(e_{ij}\right)=\;\frac{\text{exp}\left(e_{ij}\right)}{\sum_{k\in N_{i}}\text{exp}\left(e_{ik}\right)},$$ (2)

where $N_{i}$ denotes the neighborhood of node *i* in the graph. $\alpha_{ij}$ , as a long-distance dependency, can balance the contributions of the short-distance dependency in the GNN. The aggregated feature of each node that uses the distance-based graph convolution of message passing to execute the feature transformation in each layer is calculated as in Eq. 3:

$${h^{\prime }}_{i}=\sum_{j\in N_{i}}\alpha_{ij}h_{j}.$$ (3)

In the case of a large value that cannot be used in the softmax function, we also employ a basic graph convolution assigning the same importance to each neighbor.

In MP-MIM, with the increase in the number of stacked layers, we can obtain the transformed embeddings based on different degrees of neighborhood information aggregation. We set the hyperparameters *k_num* and *l_num* when the appropriate message passing type was used in different cases. The hyperparameter settings of MP-MIM are shown in Supplementary Table S1. The maximum values of *k_num* and *l_num* in the value range are recommended to be set in MP-MIM.

### Embedding evaluation by using Moran’s I statistics

Gene expressions at different spatial locations may be interdependent, and the expression values of a nearby gene may be more relevant than those farther apart according to spatial autocorrelation. Positive spatial autocorrelation means that geographically nearby values tend to be similar in space and vice versa. Spatial autocorrelation can evaluate whether a potential spatial variable gene exhibits an organized pattern and spatial layout (Hu et al., 2021). In the embedding space, the embedding integrates the input from the expression values of all genes through a deep learning model. Each dimension in the embedding space contains the underlying spatial information of all genes. For downstream analysis based on the latent variables of the embeddings, such as clustering and image segmentation, spatial autocorrelation (Griffith, 1987) provides an estimate for information content. The higher the spatial autocorrelation is, the clearer the spatial pattern is, thus indicating that the underlying embedding contains more diverse information with a suitable contrast. This process is similar to the automatic exposure correction of photos (Yuan and Sun, 2012). Therefore, the global spatial autocorrelation index can be used to evaluate the quality of embeddings with different spatial information.

Global Moran’s I is a commonly used correlation coefficient that measures global spatial autocorrelation. To evaluate the quality of a given dimension in the embedding, we calculate Moran’s I as follows:

$$\;\;\;\;\;\;\;\;\;\;\;MI=\;\frac{n\sum_{i=1}^{n}\sum_{j=1}^{n}w_{ij}\left(x_{i}-\bar{x}\right)\left(x_{j}-\bar{x}\right)}{\left(\sum_{i=1}^{n}\sum_{j=1}^{n}w_{ij}\right)\sum_{i=1}^{n}{\left(x_{i}-\bar{x}\right)}^{2}},$$ (4)

where $x_{i}$ and $x_{j}$ denote the values of spots *i* and *j*, respectively; $\bar{x}$ denotes the mean of all values; *n* denotes the number of spots; and $w_{ij}$ denotes the predefined spatial weight. The value range of Moran’s I is [−1,1], where a value close to 1 indicates a strong spatial dependence, which represents a positive correlation; a value close to −1 indicates a weak spatial dependence, which represents a negative correlation; and a value close to 0 indicates a random spatial distribution. We implement two kinds of spatial weight matrices (each denoted as $w_{ij}$ ) used in Moran’s I. One is the radius distance weight matrix, which is defined as follows:

$$w_{ij}=\{\begin{array}{c} 1,\;d_{ij}\leq \;d_{radius},\\ 0,\;d_{ij}>d_{radius}, \end{array}$$ (5)

where $d_{ij}$ denotes the Euclidian distance between two spots based on spatial coordinates and $d_{radius}$ denotes the distance threshold. The other kind of spatial weight matrix is the KNN weight matrix, in which the *k*-nearest neighbors of each spot using spatial coordinates based on the Euclidian distance are selected. We assign $w_{ij}$ = 1 between spots and their nearest neighbors; otherwise, $w_{ij}=0$ .

In MP-MIM, we use global Moran’s I with the highest correlation among different dimensions as the final evaluation result for each embedding. Global Moran’s I is calculated as follows:

$$MI_{embedding}=\text{max}\left(MI_{0},MI_{1},\ldots ,MI_{i}\right),$$ (6)

where *i* denotes the number of dimensions in embedding. When the transformed embeddings are generated with a certain hyperparameter setting of message passing, Moran’s I with maximum filtering is used to produce the final evaluation result for each embedding. Different settings of spatial weight matrices in Moran’s I are recommended according to the message passing types. The details are shown in Supplementary Table S1.

### Embedding sampling

A deep learning model can sample multiple embeddings with different hyperparameter settings by using spatial transcriptomics data. In this study, embedding sampling methods were used in the deep learning models RESEPT and SpaGCN to generate embeddings for experimental analysis. All hyperparameters in RESEPT were kept as the default except for two hyperparameters of the feature autoencoder, which integrates a variational autoencoder with positional encoding (Zhong et al., 2021). *Zdim* represents the number of latent space dimensions, and *PEalpha* represents the degree to which location information is used. RESEPT takes the spatial gene expression data as the input. RESEPT keeps the genes expressed as nonzero in more than 1% of spots and further selects the top 2,000 variable genes. Gene expression based on raw counts was used when we implemented the data preprocessing. We kept all the default parameters except for several hyperparameters in SpaGCN, including *PCA_num* (the number of dimensions of GCN input), *Resolution* (the setting of initial Louvain’s clustering), and *L_value* (the percentage of total expression contributed by neighbors). As a result, 56 three-dimensional RESEPT embeddings with different hyperparameter settings were generated for 16 samples. In total, 36 three-dimensional SpaGCN embeddings with varying hyperparameter settings were generated for six samples. Supplementary Tables S2, S3 list the details about the hyperparameter settings in RESEPT and SpaGCN, respectively.

### Evaluation metrics

The adjusted Rand index (ARI) (Hubert and Arabie, 1985) reflects the agreement between two divisions. The index measures the performance between the predicted tissue architecture and its ground truth. The index can be described as follows:

$$ARI=\frac{\sum_{ij}\left(\begin{array}{c} n_{ij}\\ 2 \end{array}\right)\;\;-\left[\sum_{i}\left(\begin{array}{c} a_{i}\\ 2 \end{array}\right)\;\sum_{j}\left(\begin{array}{c} b_{j}\\ 2 \end{array}\right)\right]/\left(\begin{array}{c} n\\ 2 \end{array}\right)\;}{\frac{1}{2}\left[\sum_{i}\left(\begin{array}{c} a_{i}\\ 2 \end{array}\right)+\;\sum_{j}\left(\begin{array}{c} b_{j}\\ 2 \end{array}\right)\right]-\left[\sum_{i}\left(\begin{array}{c} a_{i}\\ 2 \end{array}\right)+\;\sum_{j}\left(\begin{array}{c} b_{j}\\ 2 \end{array}\right)\right]/\left(\begin{array}{c} n\\ 2 \end{array}\right)\;\;\;},$$ (7)

where $n_{ij}$ denotes the number of objects in classes *i* and *j* simultaneously; and *a*_*i*_ and *b*_*j*_ represent the number of objects in classes *i* and *j,* respectively. The value range of ARI is [−1,1], where a larger value indicates a higher consistency.

The Spearman correlation coefficient with the associated *p* value (Kokoska and Zwillinger, 2000) is used to measure the performance between the baseline ranking we set in this study and the ranking generated by our evaluation method. The Spearman correlation coefficient is a nonparametric rank correlation calculated using the following equation:

$$r_{s}=1-\frac{6\sum d_{i}^{2}}{n\left(n^{2}-1\right)},$$ (8)

where *n* denotes the number of observations and $d_{i}$ marks the difference between the two rankings of each observation. The value range of the Spearman correlation is [−1,1], where a larger value means a stronger correlation. The *p*-value indicates the statistical significance of the Spearman correlation. The range of the *p*-value is [0,1], where the smaller the *p*-value is, the more significant the Spearman correlation is.

We also use global Geary’s C as the baseline for comparing different methods in the Spearman correlation analysis. Global Geary’s C is another classical index used for global spatial autocorrelation and that is defined as

$$GC=\;\frac{n-1}{2\sum_{i=1}^{n}\sum_{j=1}^{n}w_{ij}}.\;\frac{\sum_{i=1}^{n}\sum_{j=1}^{n}w_{ij}{\left(x_{i}-x_{j}\right)}^{2}}{{\sum_{i=1}^{n}\left(x_{i}-\bar{x}\right)}^{2}}.$$ (9)

The value range of Geary’s C is [0,2]. To make this range the same as that of Moran’s I, we convert the result of Geary’s C by 1-GC, where 1 means perfect positive autocorrelation, −1 means perfect negative autocorrelation, and 0 means no autocorrelation. The radius distance spatial weight matrix is used for Geary’s C (see the Embedding evaluation by using Moran’s I statistics section).

### Dataset and experimental settings

We analyzed 12 published and four 10x Genomics Visium samples generated from the human brain in this study. The four postmortem human brain samples were obtained from the study program at Banner Sun Health Research Institute (Beach et al., 2015) and New York Brain Bank at Columbia University Medical Center (Vonsattel et al., 2008). Two samples are from non-AD cases at Braak stages I and II (namely, samples 2–5 and 18–64), and the other two are from early-stage AD cases at Braak stages III and IV (namely, samples 2–8 and T4857). The details about the number of ground-truth labels and spots for each sample are shown in Supplementary Table S4.

The proposed method MP-MIM and other algorithm models were implemented by Python 3.6.8. All algorithms were run on a computing server with 2.2 GHz, 144-core CPU, and 503 GB RAM under an Ubuntu 18.04 operating system.

We implemented the K-means algorithm (MacQueen., 1967) on 56 embeddings for each sample by using the cluster number from the ground-truth labels and measured ARI by the result of K-means and the ground-truth labels. The value of ARI was considered the quality representation of each embedding, and the ranking of ARI was used as the ground-truth ranking in this study. To validate our proposed method, we evaluated three performance metrics, which are the top 5 results of the ranking, the Spearman correlation (which is measured by the rankings of different comparison methods and ground-truth ranking), and the effectiveness of integrating MP-MIM into the SpaGCN and RESEPT models. In the case of the top 5 metric, using native Moran’s I to measure the first, second, and third dimensions of each embedding is set as the baseline method. In the case of the Spearman correlation metric, both native Moran’s I and native Geary’s C were used to measure the first, second, and third dimensions of each embedding as the baseline methods. Finally, the baseline method that can obtain the highest Spearman correlation among all dimensions is used for comparison with MP-MIM.

## Results

Our proposed method performed competitively on three evaluation metrics in this study. The top 5 rankings generated by MP-MIM can identify high-quality embedding on most spatial transcriptomics samples. Integrating MP-MIM into two bioinformatics tools used for spatial transcriptome analysis shows that the performance of tissue architecture identification can be improved in most cases.

### High-quality embedding can be identified among the top 5

To set a comprehensive comparison metric, we used the quality of the top 5 embeddings rather than the top 1 in the ranking to assess an evaluation method. The top 5 evaluation is also applied to other research problems (Xiong et al., 2017; Chang et al., 2020). The top 5 in the ranking represents the first five high-quality embeddings among all input embeddings identified by our evaluation method. The higher the quality of each of the top 5 embeddings is, the more capable our evaluation method is considered. This study mainly validated the top 5 RESEPT embeddings according to native Moran’s I and MP-MIM for 16 samples. In MP-MIM, a distance-based graph convolution was used by setting *k_num* to be 90 and *l_num* to be 15. To better show the effect before and after message passing, the original and corresponding graph embeddings were transformed into RGB images by the conversion method in RESEPT (Chang et al., 2021).

RGB images of the original and the transformed embeddings that are top 5 ranked by MP-MIM are visualized in Supplementary Figure S1. As shown in Supplementary Figure S1, the top 5 RGB images have a consistent pattern in almost all 16 samples, with better color contrast and clearer architectures in the transformed embeddings than the original embeddings. Message passing as a process of smoothing information can help aggregate more original graph information. The embedding values are converted into pixel values by using the RESEPT method. RGB images can be better visualized after embedding transformation. Supplementary Figure S2 shows the ARI comparison results between the top 5 original embeddings and the corresponding transformed embeddings on the 16 samples. As shown in Supplementary Figure S2, the top 5 original embeddings can obtain a higher ARI when transformed by MP-MIM in most cases. Among the 12 samples (151507, 151508, 151509, 151670, 151671, 151672, 151673, 151674, 151676, 2–5, 18–64, and T4857), the embedding transformation based on the message passing of MP-MIM can improve at least over three original embeddings in the top 5 in terms of ARI results when using the embedding; in the two samples (151671 and 151673), the embedding transformation improved all original embeddings in the top 5.

**FIGURE 2:**
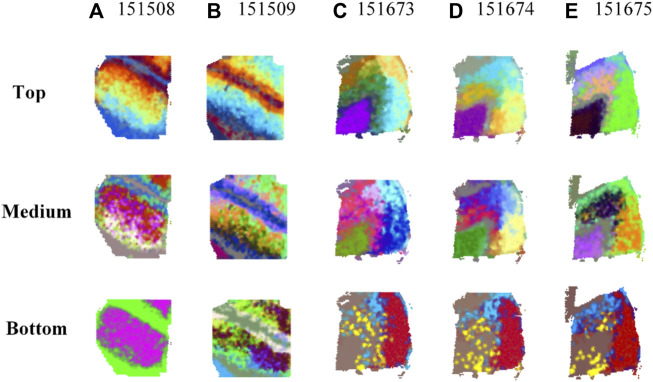
Comparison results of top-, medium-, and bottom-ranked RGB images based on transformed embeddings in MP-MIM on **(A)** 151508, **(B)** 151509, **(C)** 151673, **(D)** 151674, and **(E)** 151675 samples.

Comparison results of top-, medium-, and bottom-ranked RGB images based on transformed embeddings in MP-MIM rankings on five samples are shown in Figure 2. The results of the remaining 11 samples are shown in Supplementary Figure S3. Compared with the medium and bottom RGB images, the top RGB images are clear with less noise, as shown in Figure 2. Image layout and architecture can be clearly visible from top-ranked RGB images. The underlying spatial patterns of the embeddings are shown in the transformed RGB images. The clearer the spatial pattern of the RGB image is, the more diverse information and features the embedding contains, thus indicating that the embedding is a higher-quality embedding. Disorganized and fuzzy patterns are shown in the bottom-ranked RGB images. The image effects of medium-ranked RGB images are between those of top- and bottom-ranked images. MP-MIM can accurately identify the embeddings with different qualities.

The top 5 embeddings in the rankings of native Moran’s I and MP-MIM for RESEPT are shown in Supplementary Table S5. Among the comparison methods, the embedding achieving the clustering result closest to the ground truth is among the top 5 selected by MP-MIM on 10 out of 16 samples (151507, 151508, 151509, 151510, 151669, 151670, 151671, 151672, 151673, and 18–64). By applying message passing, each node aggregates information constantly from its neighborhood, and the underlying biological information of each embedding can be explicitly expressed. Message passing helps Moran’s I with maximum filtering to evaluate and identify the high-quality embeddings. Therefore, the overall high-quality top 5 embeddings can be obtained when MP-MIM is used.

In addition, Supplementary Table S7 compares the top 5 embeddings of the SpaGCN model for native Moran’s I and MP-MIM methods based on the ground truth on six samples (151507, 151508, 151509, 151510, 18–64, and T4857). In MP-MIM, the basic graph convolution was used with setting *k_num* to be 9 and *l_num* to be 15 for SpaGCN embedding. Overall, the top 5 selections have some good results, but are not as good as in RESEPT.

### MP-MIM predicts accurate rankings according to high Spearman correlation with the ground truth

A higher Spearman correlation means that the two rankings are more similar. First, we measured the Spearman correlation between the ground-truth ranking and the ranking evaluated by the baseline method (see the “Dataset and experimental settings” section) and MP-MIM on the RESEPT embedding for 16 samples. MP-MIM was used by a distance-based graph convolution of message passing with the values of *k_num* to be 90 and *l_num* to be 15. Then, the SpaGCN embedding on six samples was applied with the basic graph convolution, and the setting *k_num* to be 9 and *l_num* to be 15 in MP-MIM. If the Spearman correlation is a negative value, we set the value at zero.

**FIGURE 3:**
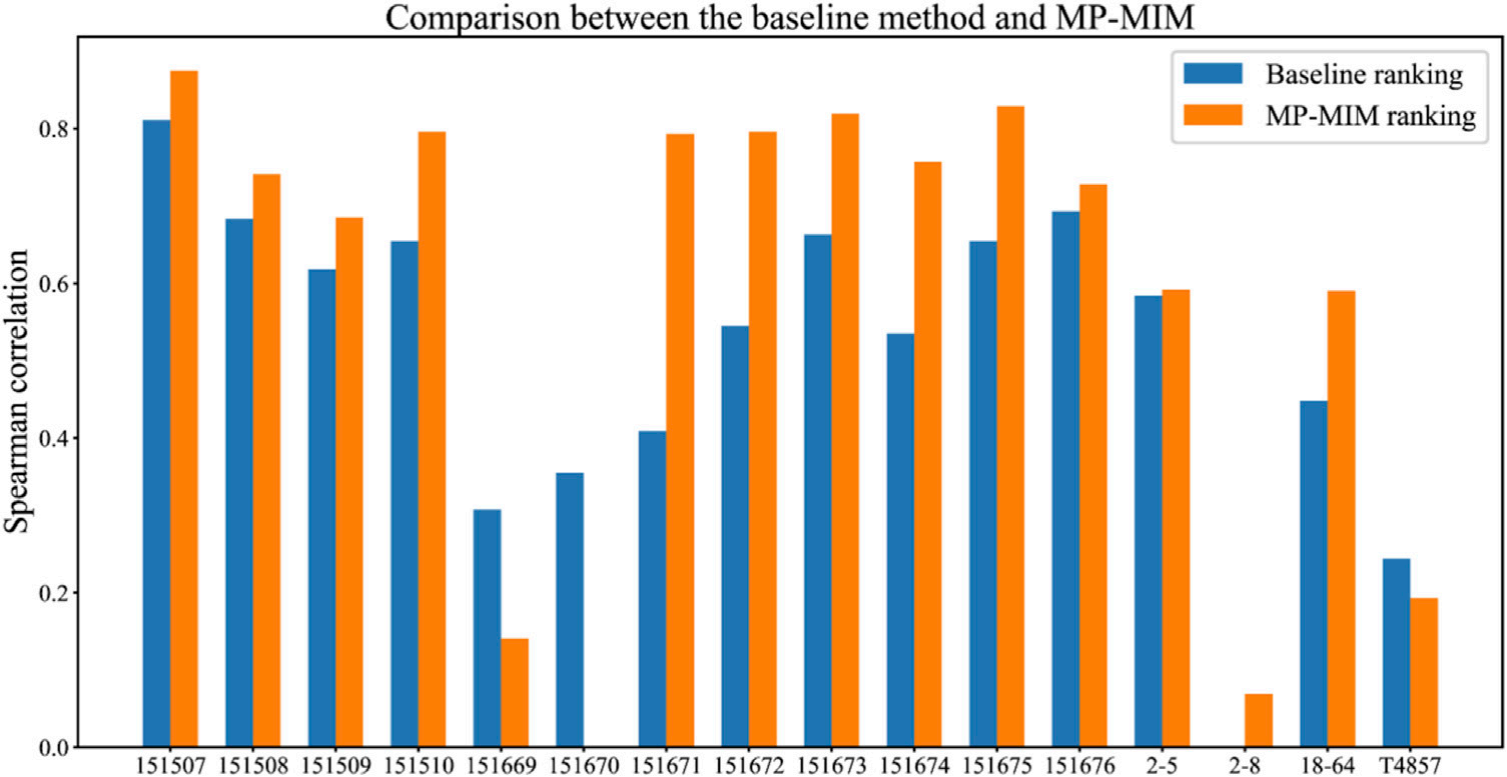
Comparison between the baseline method (without applying MP-MIM) and MP-MIM on the 16 samples. In this study, Spearman correlation coefficients of the baseline ranking are measured between the ground-truth ranking based on embedding quality according to the ARI and the ranking of the baseline method. The Spearman correlation coefficients of the MP-MIM ranking are measured using the ground-truth ranking and the ranking of the MP-MIM.

Figure 3 shows the comparison results between the baseline method and MP-MIM. A higher-correlation ranking with the ground truth can be obtained by MP-MIM in which the hyperparameters *k_num* and *l_num* are 90 and 15, respectively. The Spearman correlations on 13 out of 16 samples are improved to different degrees, especially on sample 151671, the performance of MP-MIM is two times higher than the performance of the baseline method using the original embedding. In most samples, except for 151669, 151670, 2–8, and T4857, the Spearman correlations are larger than 0.5, thus indicating that the rankings are highly consistent with the ground truth. Supplementary Table S6 compares the baseline method in terms of Moran’s I or Geary’s C on each dimension of the original embedding and MP-MIM. In this study, the baseline ranking is generated by the best performance of the baseline methods. In most cases, the Spearman correlations of Moran’s I are higher than those of Geary’s C on the same dimension of the original embedding. The highest Spearman correlation can be obtained using Moran’s I to measure the first dimension (emb0) and the third dimension (emb2) on samples 151669 and T4857, respectively. Using Geary’s C to measure the second dimension can obtain the best Spearman correlation among all comparison methods on sample 151670.

**FIGURE 4:**
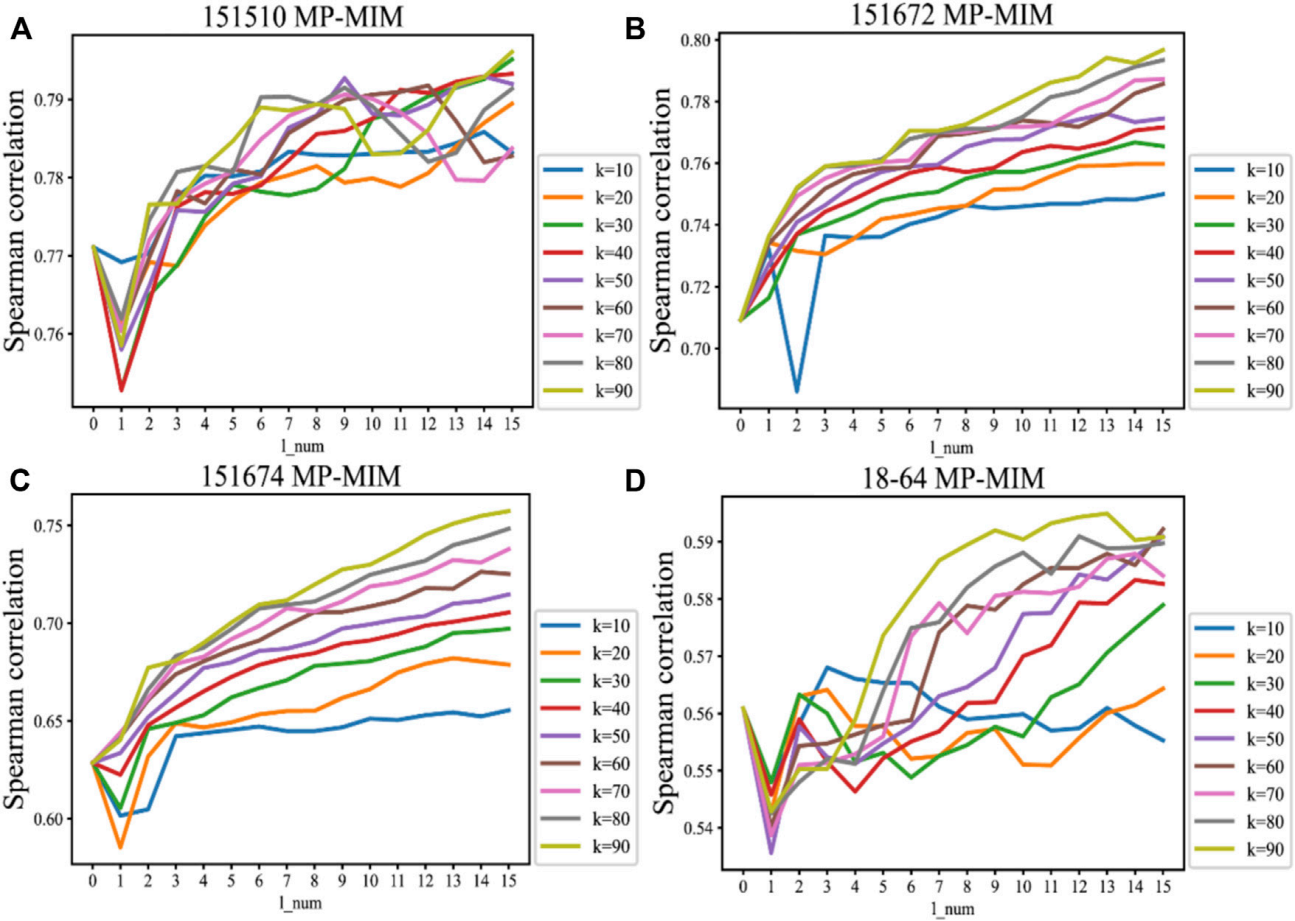
Sensitivities of the hyperparameters *k_num* and *l_num* in message passing to the Spearman correlation on the following samples: **(A)** 151510, **(B)** 151672, **(C)** 151674, and **(D)** 18–64. The horizontal axis represents the number of layers in the message passing. The vertical axis represents the Spearman correlation measured by the ground-truth ranking and the ranking of the MP-MIM. *k* denotes the number of nearest neighbors, which is the predefined parameter in the KNN graph.

The sensitivities of the hyperparameters *k_num* and *l_num* in message passing on four samples are shown in Figure 4. These sensitivities reflect the value of Spearman correlation changes with the hyperparameters *k_num* and *l_num* in MP-MIM. The Spearman correlation becomes larger as the degree of message passing increases, and in most cases, the correlation reaches the maximum when *k_num* is 90 and *l_num* is 15. With message passing, each embedding can express more representative features and be more suitable for Moran’s I to rank. When the number of aggregation layers is larger, the rankings are more consistent with the ground truth. As *k_num* increases, the spots can obtain more neighborhood nodes in the graph and aggregate more information in each iteration. When *k_num* is 90, MP-MIM usually outperforms the other hyperparameter settings of *k_num*. Only when *k_num* is 10 does message passing not constantly help MP-MIM for ranking, such as on samples 18–64. Supplementary Figure S4 demonstrates the significance of each Spearman correlation and reflects the *p*-value changes with the hyperparameters *k_num* and *l_num* in MP-MIM. In 12 samples (151507, 151508, 151509, 151510, 151671, 151672, 151673, 151674, 151675, 151676, 2–5, and 18–64), all of the *p*-values are smaller than 0.01. The *p*-value decreases as the degree of message passing increases, and in most cases, the *p*-value reaches the minimum when *k_num* is 90 and *l_num* is 15. Our results show a significant Spearman correlation between the ranking of MP-MIM and the ground truth.

The sensitivities of more settings of hyperparameters *k_num* and *l_num* in message passing are shown in Supplementary Figures S5–S7. As shown in Supplementary Figure S5, the changing trend of sensitivity continues when the hyperparameters *k_num* and *l_num* are increased. In most cases, the greater the degree of message passing, the more accurate identification MP-MIM can achieve on all input embeddings. More neighborhood information aggregation helps the embeddings better express features and are accurately evaluated. Supplementary Figures S6, S7 detail the cases of large settings of hyperparameters *k_num* and *l_num*. They show that the increasing trend of the Spearman correlation can eventually plateau and that the Spearman correlation converges when the hyperparameters *k_num* and *l_num* are large enough. Therefore, to reduce the complexity of MP-MIM and obtain a promising result, the hyperparameter settings of *k_num* = 90 and *l_num* = 15 are used in this study.

**FIGURE 5:**
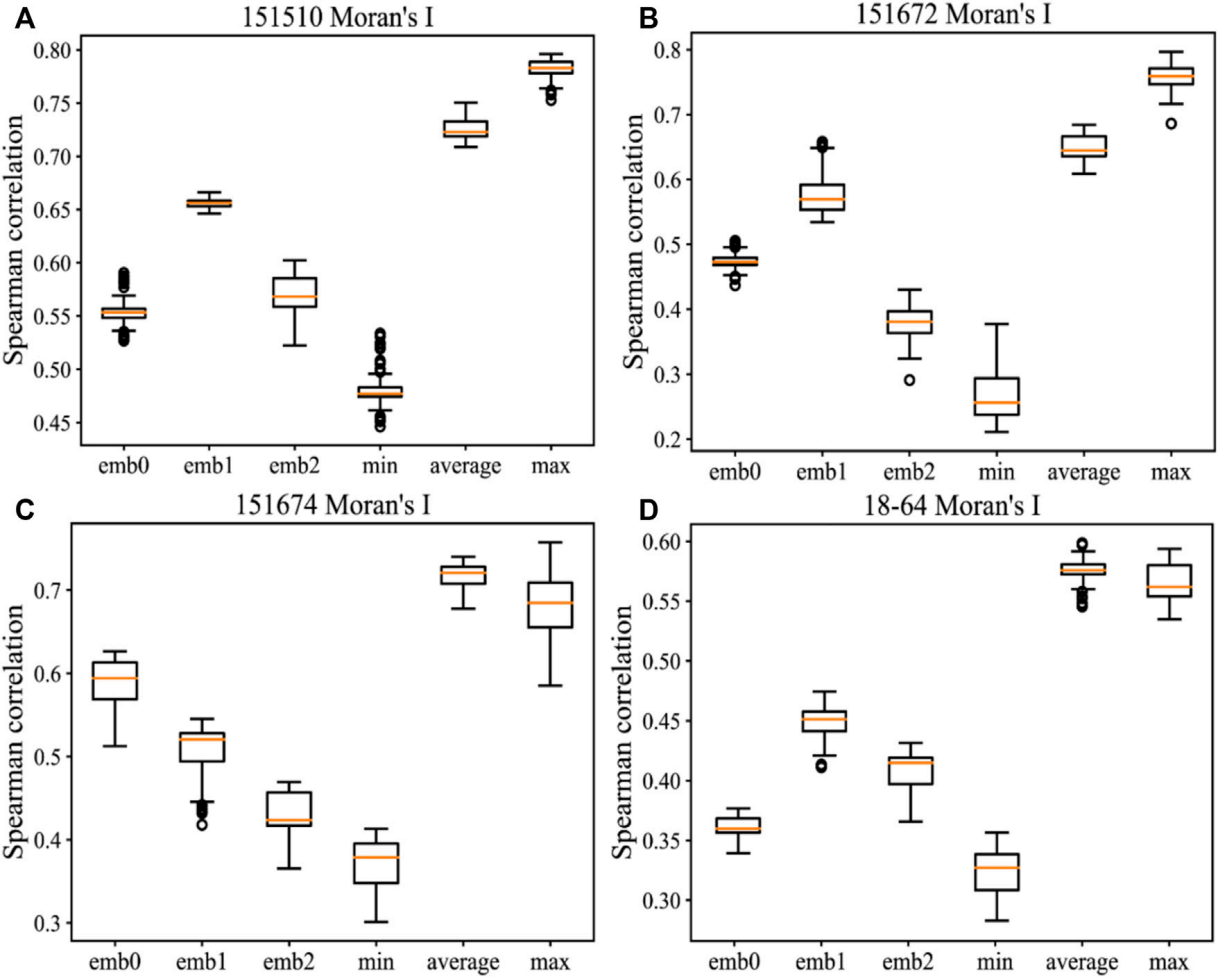
Comparison of different methods for measuring Moran’s I on the following samples: **(A)** 151510, **(B)** 151672, **(C)** 151674, and **(D)** 18–64. The horizontal axis shows the methods of directly measuring Moran’s I on the first dimension (emb0), second dimension (emb1), and third dimension (emb2) in each embedding and the methods of combining Moran’s I with the minimum, average, and maximum filtering in all dimensions. The vertical axis shows the Spearman correlation measured by the ground-truth ranking and the ranking by MP-MIM. Each box shows all results in message passing with *k_num* values from 10 to 90 and *l_num* values from 1 to 15.

During the experiments, we also compared the Spearman correlations of minimum and average filtering on Moran’s I. Figure 5 details the comparison results of different dimensions and minimum, average, or maximum filtering on Moran’s I by applying message passing on four samples. Compared with minimum filtering, the maximum and average filtering on Moran’s I can contribute more to the evaluation method when message passing is applied. However, the minimum filtering negatively affects the evaluations of embeddings. The variation ranges of the maximum filtering based on message passing are larger than those of the average filtering, and the optimal Spearman correlation can be obtained in most cases. Hence, the maximum filtering on Moran’s I is the most effective method. Spearman correlations of three dimensions within the independent variation range change inconsistently, thus indicating that different dimensions of this embedding contain individual and diverse information and features. Supplementary Figure S8 lists the sensitivity results on the hyperparameters *k_num* and *l_num* in message passing, and Supplementary Figure S9 shows the same comparison results as Figure 5 on the remaining 12 samples. In addition, Supplementary Table S8 compares the baseline method in terms of Moran’s I or Geary’s C on each dimension of the original embedding and MP-MIM for SpaGCN. Supplementary Figure S10 shows the sensitivity of the hyperparameters *k_num* and *l_num* in message passing on six SpaGCN embedding samples.

### Result analysis in embedding data distribution

We selected samples 151507, 151510, and 151673 with promising Spearman correlations and samples 151669, 151670, and 2–8 with poor Spearman correlations to further analyze the distribution of graph embedding. Supplementary Figure S11 lists the distributions of top-, medium-, and bottom-ranked graph embeddings in MP-MIM rankings on six RESEPT embedding samples.

As shown in Supplementary Figure S11, the distributions of top embeddings are scattered and well-proportioned under a stable density change of all dimensions in most cases, and the distributions of bottom-ranked embeddings have nonuniform density variations with an apparent accumulation around zero. The distributions of medium embeddings are usually between top-ranked embeddings and bottom-ranked embeddings. For samples 151669 and 2–8, there is one dimension with a poor proportion distribution in the top embedding, and there are two dimensions with density distribution accumulation around zero in the top embedding. The ranks of the last embeddings of samples 151669, 151670, and 2–8 in the corresponding ground-truth rankings are shown in the front. After observing all distributions of 56 embeddings for each sample, the percentages of embeddings under nonuniform density distribution with an obvious accumulation around zero of 151669, 151670, and 2–8 samples are larger than those of other samples. The higher percentage of low-quality embeddings may confuse Moran’s I to evaluate and mislead the final output ranking of MP-MIM. In this study, a few cases have poor Spearman correlations, and we can obtain competitive performance in the top 5 but only with low Spearman correlation results, such as on sample 151669.

### Effectiveness of integrating MP-MIM into models

To validate the effectiveness of integrating our proposed method into the model by using spatial transcriptomics data, SpaGCN and RESEPT were used as the validation objects. We kept all the hyperparameters of SpaGCN and RESEPT as default and took the gene expression based on raw count as the input for both models. Supplementary Table S1 details the hyperparameter settings of MP-MIM. The histology and the ground truth for each sample are shown in Supplementary Figures S12, S13, respectively. Compared with the performances of the original models, the performances improved after integrating MP-MIM in both cases of RESEPT and SpaGCN.

**FIGURE 6:**
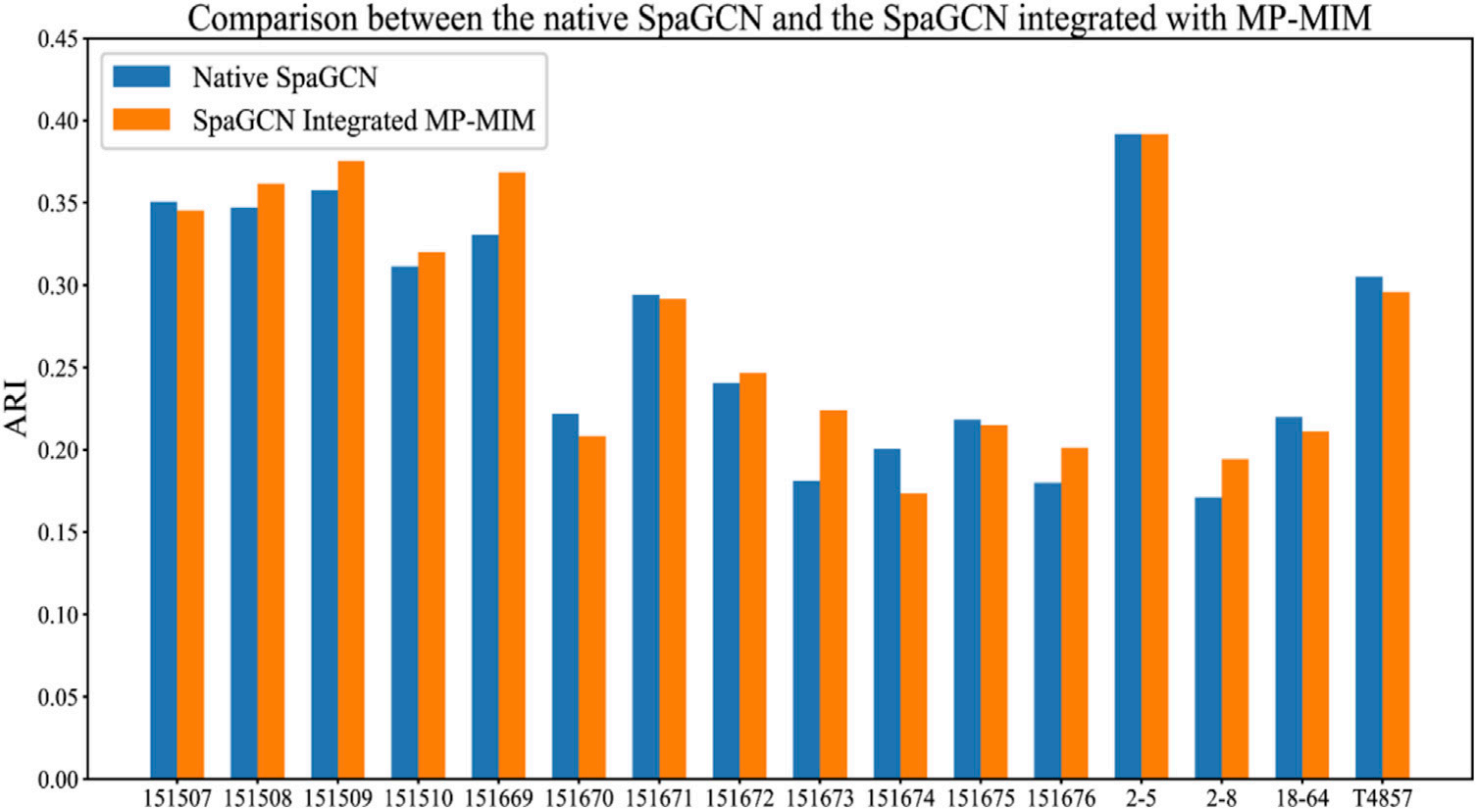
Comparison between the original SpaGCN and the SpaGCN integrated with MP-MIM. The results of the original SpaGCN are obtained by keeping all hyperparameters of SpaGCN as default, and SpaGCN integrated with MP-MIM shows the results after integrating MP-MIM into the original SpaGCN.

SpaGCN uses GCN to generate embedding based on gene expression, spatial location, and histology information of spatial transcriptomics data and then employs unsupervised iterative clustering based on the embedding of GCN to separate spots into different spatial domains. We inserted MP-MIM into the model to enhance the spatial tissue architecture identification before implementing unsupervised iterative clustering. The K-means algorithm with the cluster number of corresponding sample labels was used for the initial clustering in SpaGCN. MP-MIM started to transform the embedding of the original SpaGCN into multiple embeddings with different degrees of message passing and selected the transformed embedding with the highest value of Moran’s I with maximum filtering in the next training step. A basic graph convolution of message passing was applied in MP-MIM. We compared the ARI of the original SpaGCN integrated with MP-MIM with the ARI calculated by the original SpaGCN. The comparison details of the 16 samples are shown in Figure 6, and the tissue architecture identifications of SpaGCN and SpaGCN integrated with MP-MIM are illustrated in Supplementary Figure S12. MP-MIM improves the performance of SpaGCN on 9 out of 16 samples. Embedding transformation using message passing can enhance identifying the spatial tissue architecture. Some samples, such as 151669 and 151673, have a more obvious improvement.

**FIGURE 7:**
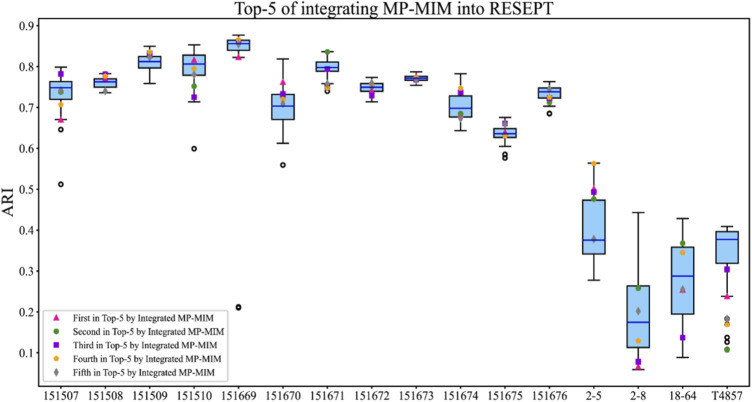
Top 5 tissue architecture identifications of integrating MP-MIM into RESEPT. The blue horizontal line indicates the median ARI of all values, and this median value is set as the baseline on each sample in this study. The top 5 ARI values are marked in different colors and shapes.

In the case of RESEPT, MP-MIM was used to evaluate the generative embeddings with multiple hyperparameter settings before being converted into RGB images. RESEPT semantically segmented these RGB images converted by the original embeddings to identify the spatial tissue architecture. The ranking of the embeddings with the corresponding tissue architecture identification results can be obtained after integrating MP-MIM into the original RESEPT. Measuring the results of the top 5 is commonly used in spatial analysis. We defined the median value of all ARIs obtained by RESEPT as the baseline ARI. Figure 7 details the spatial tissue architecture identifications on the 16 samples. The top 5 ARI values are marked in different colors and shapes. As shown in Figure 7, compared with the ARI value of the baseline, the higher ARI value can be obtained from the top 5 ARI values of MP-MIM on 15 out of 16 samples. On three samples (151509, 151670, and 2–5), all the top 5 ARI values obtained by MP-MIM are higher than those obtained by the baseline method. On samples 151508, 151671, and 2–5, RESEPT obtains the best architecture identifications from the top 5 ARI values after the method is integrated with MP-MIM. Supplementary Figure S13 illustrates the baseline segmentation with the median value of all ARIs and the top 5 segmentations from RESEPT integrated with MP-MIM for each sample.

In the two cases, each sample took more than several hours and a half hour of computing time on a desktop machine when MP-MIM was integrated into SpaGCN and RESEPT. The computational scalability of embedding sampling methods and MP-MIM depends on the numbers of cells, genes, and tissue types.

### Software tool

The proposed method, MP-MIM, can be used as a software tool to support tissue architecture identification in spatial analysis; the software tool is available at https://github.com/YuLin-code/MP-MIM. The software tool can be easily integrated with high compatibility for computational methods involving generating embeddings for tissue architecture identification by using gene expression and spatial locations as the input. Additionally, MP-MIM provides a novel perspective to evaluate and enhance the embeddings generated by the deep learning model by using spatial transcriptomics data.

MP-MIM can be used as two kinds of software tools according to different cases. For spatial methods, such as SpaGCN, generative embedding during training is directly used for clustering. Our tool can be a supplement to generate graph-based embeddings and identify the most effective embedding for further clustering. For spatial methods such as RESEPT, generative embeddings with various hyperparameter settings are used to produce RGB images and conduct image segmentation. We can apply the tool to select high-quality generative embeddings for downstream analysis.

## Discussion and conclusion

This study proposed an embedding evaluation method, MP-MIM, used for spatial transcriptome analysis. MP-MIM can accurately evaluate multiple sampling embeddings with different hyperparameter settings and identify high-quality embeddings by producing a high correlation between the predicted spatial architecture and the ground truth. The combination of message passing and Moran’s I with maximum filtering was used for selecting the most effective embedding. Sixteen spatial transcriptomics samples based on the human brain were used to validate the proposed method in our experiments. The method of directly using Moran’s I or Geary’s C to measure different dimensions of the evaluated embeddings was set as the baseline. We also integrated the proposed method into two models, SpaGCN and RESEPT, to validate performance.

The experimental results show that compared with the baseline method on most samples, MP-MIM can accurately identify higher-quality embeddings from the top 5 results and rank embeddings with a higher and more significant Spearman correlation between MP-MIM’s output ranking and the ground truth. Using message passing, the MP-MIM can predict a more precise ranking for evaluating embeddings. With a greater degree of message passing employed, the ranking closer to the ground truth can be output by the MP-MIM. Compared with Geary’s C, Moran’s I obtains a higher Spearman correlation between the predicted ranking and the ground-truth ranking of the original embeddings. Moran’s I appeared to be better suited to measure spatial autocorrelation for spatial transcriptome analysis. Integrating MP-MIM improves the performance of SpaGCN and RESEPT on 9 out of 16 samples and 15 out of 16 samples, respectively. The promising results are usually based on sufficient message passing in MP-MIM. Overall, MP-MIM enhances the identification of spatial tissue architecture in spatial transcriptome analysis.

In summary, the main innovations of this study are message passing-based embedding transformation for better expressing the underlying information and using spatial autocorrelation indices to evaluate embedding quality. In message passing, the graph built using the expression values of each spot offers the communication path for neighborhood information aggregation. With message passing, each spot can aggregate more information from the spot’s neighborhood with further distance, and the original information of the entire graph can be presented more obviously. The original embeddings are commonly generated by a deep learning model with a few stacked layers. In this study, the transformed embeddings based on aggregating information from the remote neighborhood can represent comprehensive values involving near and remote neighborhood information of the input spatial transcriptomics data. Moran’s I is used to evaluate embedding quality rather than to detect spatially variable genes in general spatial analysis. Moran’s I can obtain the most representative result of spatial autocorrelation among different dimensions of each embedding with maximum filtering. Consequently, the proposed method MP-MIM can accurately identify high-quality embeddings.

Sample dependency is the main limitation for cases in which the training and validation data sizes are relatively small. Selecting the most representative embedding can help improve the performance by using few spatial transcriptomics data, as MP-MIM produces a sampling of each data and identifies a high-quality embedding by selecting the appropriate embedding to better represent the experimental data to serve for downstream analysis. The method of MP-MIM can also be extended and recommended to any spatial transcriptome analysis tool. In this study, the ground-truth labels are manually annotated by expert, and the spatial spots in the tissues can be assigned to different layers. The ARI values are low in most cases, probably due to the clustering method we employed. The purpose of this article was not to explore the best clustering methods, and our study can be applied to any clustering method. However, MP-MIM may not always improve analysis performance. In several cases in this study, integrating a method with MP-MIM did not improve sample performances, such as the performance of SpaGCN integrated with MP-MIM on 151674 and the performance of RESEPT integrated with MP-MIM on T4857. Improving the computing time of MP-MIM and using it as a built-in regularization in deep architecture design need to be further explored. More types of spatial transcriptomics data with different sequencing methods will be used for analysis and verification in the future.

## Data Availability

The 12 published 10x Genomics Visium samples are generated from the human brain and can be accessed from https://www.10xgenomics.com/products/spatial-gene-expression. The four AD samples (2-5, 2-8, 18-64, T4857) are available upon request.
