# Supplementary material for: Sampling and ranking spatial transcriptomics data embeddings to identify tissue architecture
Source: Front Genet. 2022 Aug 12;13:912813. doi: 10.3389/fgene.2022.912813 (PMC9411666; doi:10.3389/fgene.2022.912813)
Supplement: Supplementary file 1 [file DataSheet1.PDF]

Supplementary Materials

1 Supplemental Figures

1.1 Supplemental Figure S1

1.1.1 151507

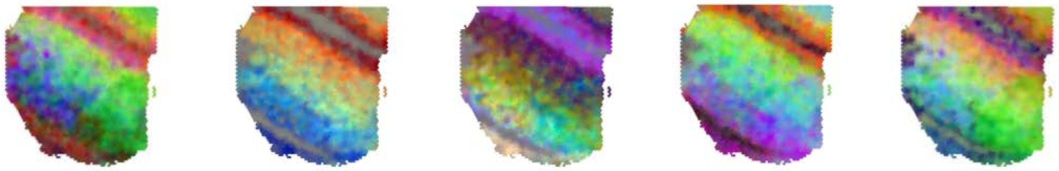

(A) First native embedding. (B) Second native embedding. (C) Third native embedding. (D) Fourth native embedding. (E) Fifth native embedding.

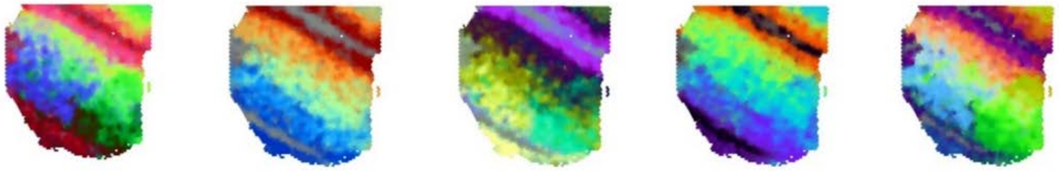

(F) First graph embedding. (G) Second graph embedding. (H) Third graph embedding. (I) Fourth graph embedding. (J) Fifth graph embedding.

1.1.2 151508

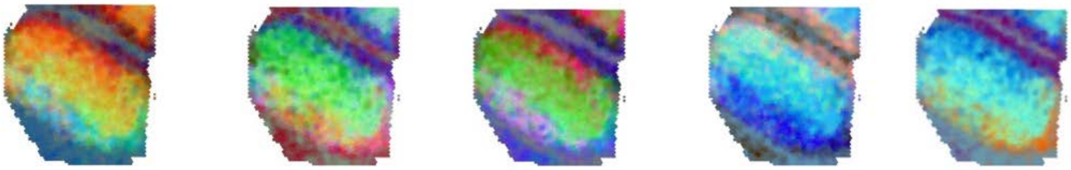

(A) First native embedding. (B) Second native embedding. (C) Third native embedding. (D) Fourth native embedding. (E) Fifth native embedding.

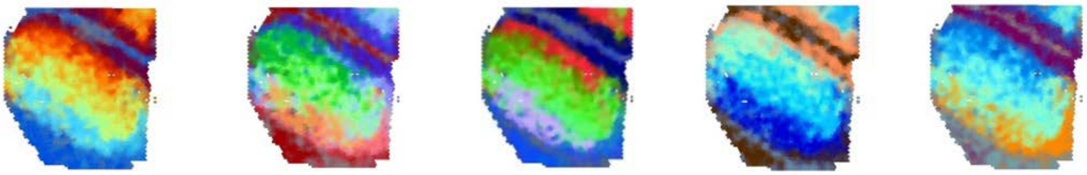

(F) First graph embedding. (G) Second graph embedding. (H) Third graph embedding. (I) Fourth graph embedding. (J) Fifth graph embedding.

1.1.3 151509

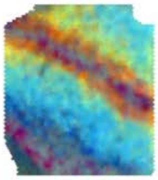
(A) First native embedding.

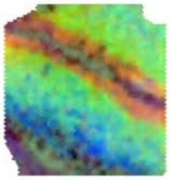
(B) Second native embedding.

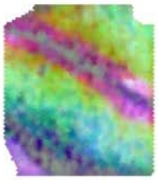
(C) Third native embedding.

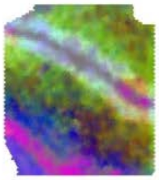
(D) Fourth native embedding.

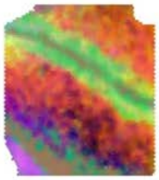
(E) Fifth native embedding.

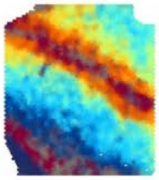
(F) First graph embedding.

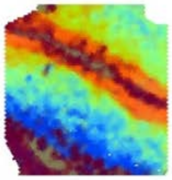
(G) Second graph embedding.

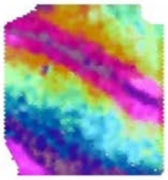
(H) Third graph embedding.

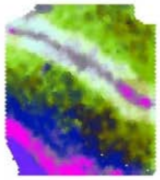
(I) Fourth graph embedding.

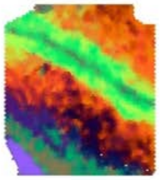
(J) Fifth graph embedding.

1.1.4 151510

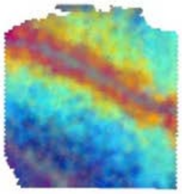
(A) First native embedding.

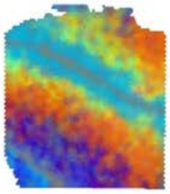
(B) Second native embedding.

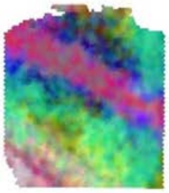
(C) Third native embedding.

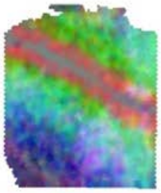
(D) Fourth native embedding.

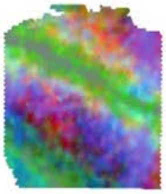
(E) Fifth native embedding.

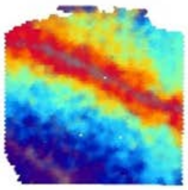
(F) First graph embedding.

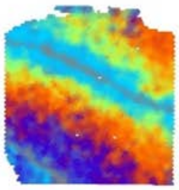
(G) Second graph embedding.

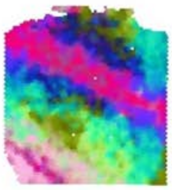
(H) Third graph embedding.

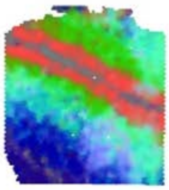
(I) Fourth graph embedding.

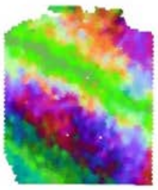
(J) Fifth graph embedding.

1.1.5 151669

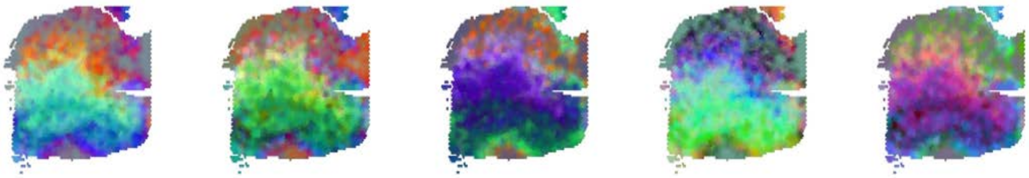

(A) First native embedding. (B) Second native embedding. (C) Third native embedding. (D) Fourth native embedding. (E) Fifth native embedding.

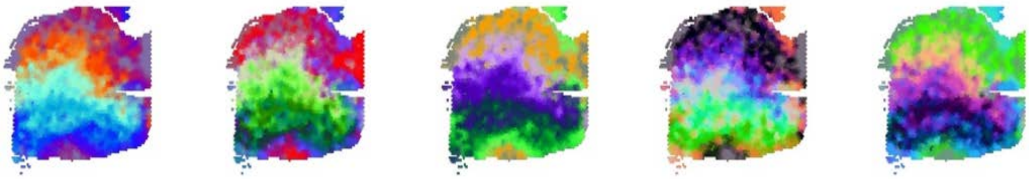

(F) First graph embedding. (G) Second graph embedding. (H) Third graph embedding. (I) Fourth graph embedding. (J) Fifth graph embedding.

1.1.6 151670

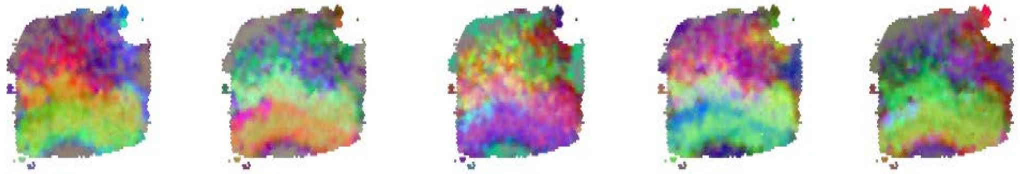

(A) First native embedding. (B) Second native embedding. (C) Third native embedding. (D) Fourth native embedding. (E) Fifth native embedding.

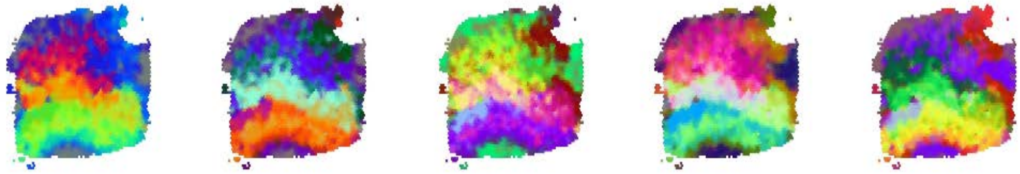

(F) First graph embedding. (G) Second graph embedding. (H) Third graph embedding. (I) Fourth graph embedding. (J) Fifth graph embedding.

1.1.7 151671

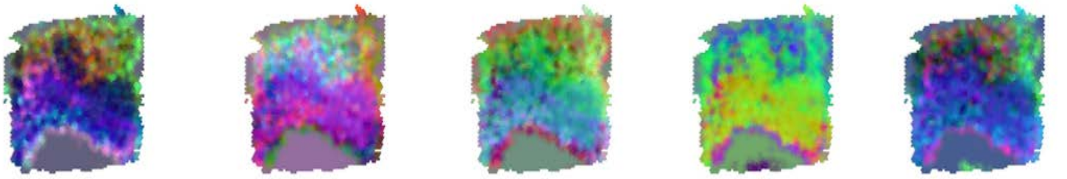

(A) First native embedding. (B) Second native embedding. (C) Third native embedding. (D) Fourth native embedding. (E) Fifth native embedding.

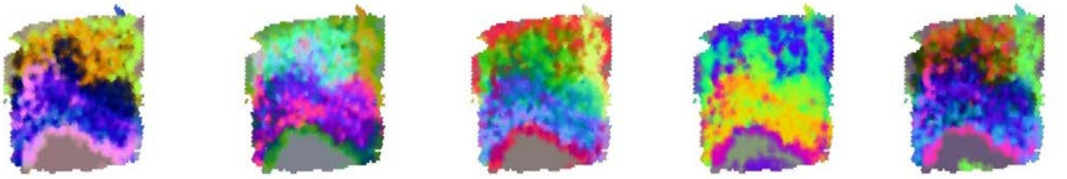

(F) First graph embedding. (G) Second graph embedding. (H) Third graph embedding. (I) Fourth graph embedding. (J) Fifth graph embedding.

1.1.8 151672

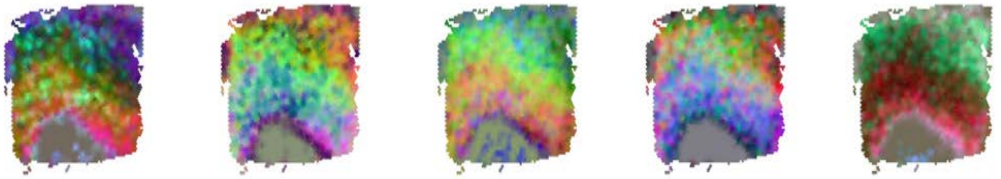

(A) First native embedding. (B) Second native embedding. (C) Third native embedding. (D) Fourth native embedding. (E) Fifth native embedding.

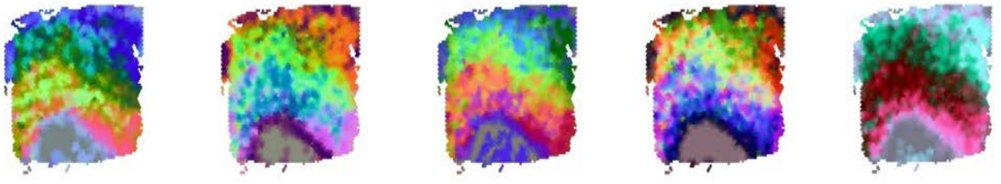

(F) First graph embedding. (G) Second graph embedding. (H) Third graph embedding. (I) Fourth graph embedding. (J) Fifth graph embedding.

1.1.9 151673

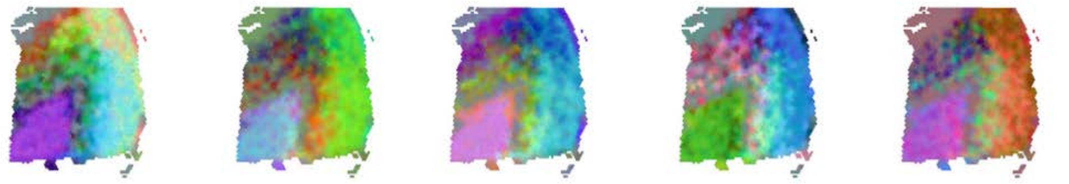

(A) First native embedding. (B) Second native embedding. (C) Third native embedding. (D) Fourth native embedding. (E) Fifth native embedding.

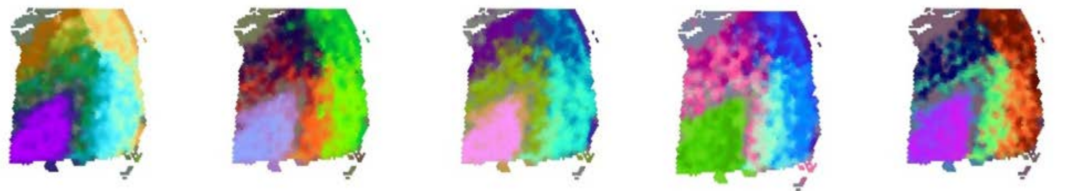

(F) First graph embedding. (G) Second graph embedding. (H) Third graph embedding. (I) Fourth graph embedding. (J) Fifth graph embedding.

1.1.10 151674

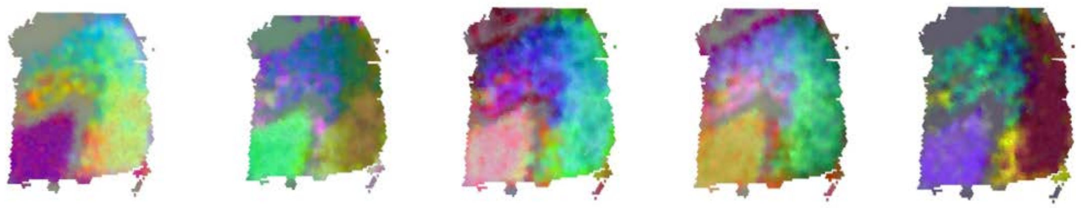

(A) First native embedding. (B) Second native embedding. (C) Third native embedding. (D) Fourth native embedding. (E) Fifth native embedding.

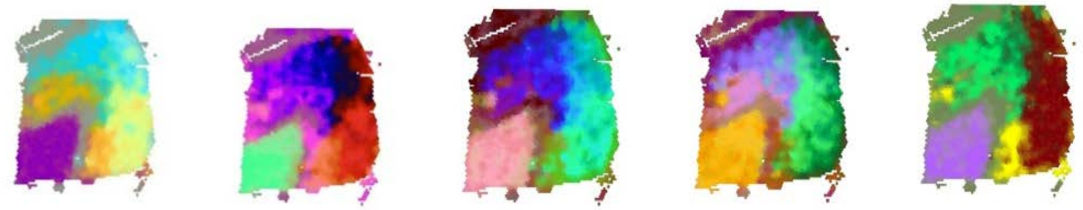

(F) First graph embedding. (G) Second graph embedding. (H) Third graph embedding. (I) Fourth graph embedding. (J) Fifth graph embedding.

1.1.11 151675

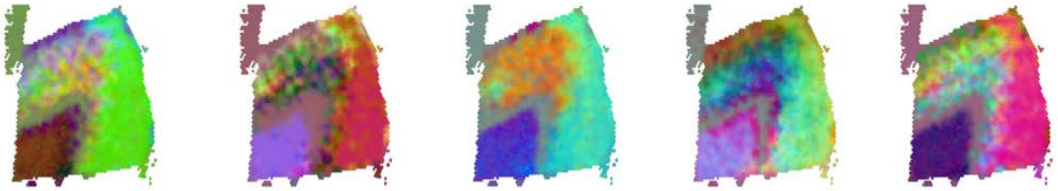

(A) First native embedding. (B) Second native embedding. (C) Third native embedding. (D) Fourth native embedding. (E) Fifth native embedding.

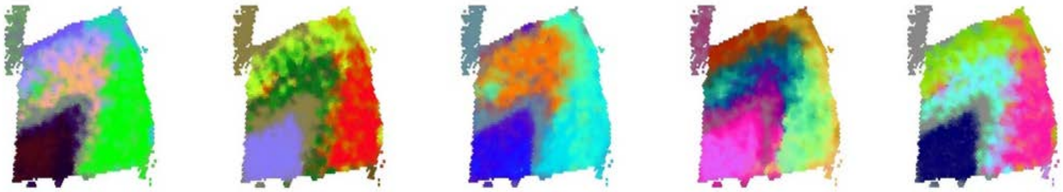

(F) First graph embedding. (G) Second graph embedding. (H) Third graph embedding. (I) Fourth graph embedding. (J) Fifth graph embedding.

1.1.12 151676

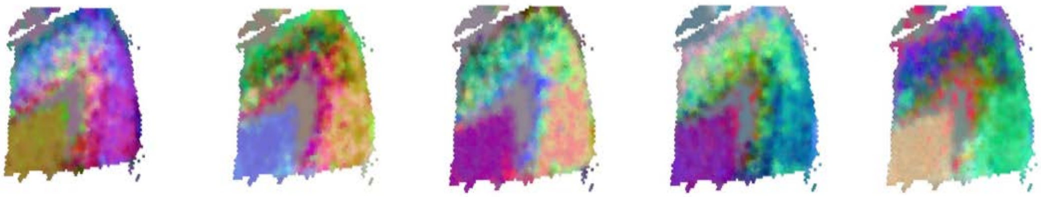

(A) First native embedding. (B) Second native embedding. (C) Third native embedding. (D) Fourth native embedding. (E) Fifth native embedding.

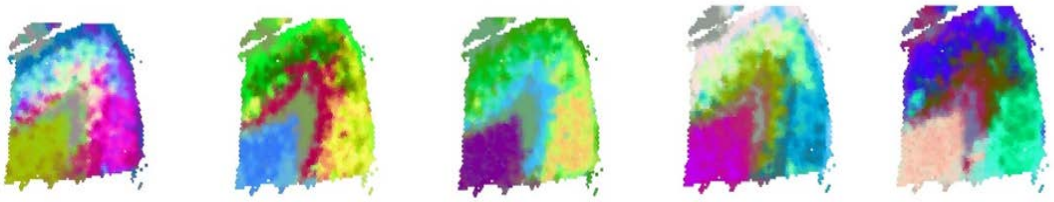

(F) First graph embedding. (G) Second graph embedding. (H) Third graph embedding. (I) Fourth graph embedding. (J) Fifth graph embedding.

1.1.13 2-5

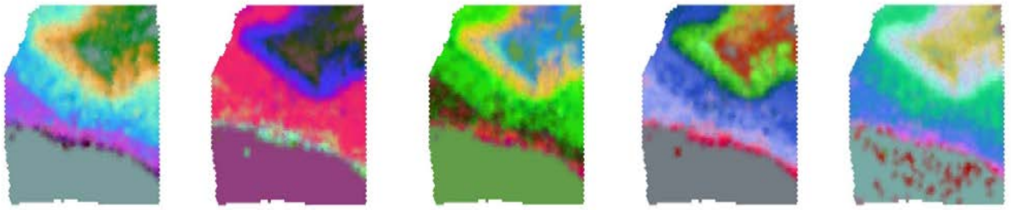

(A) First native embedding. (B) Second native embedding. (C) Third native embedding. (D) Fourth native embedding. (E) Fifth native embedding.

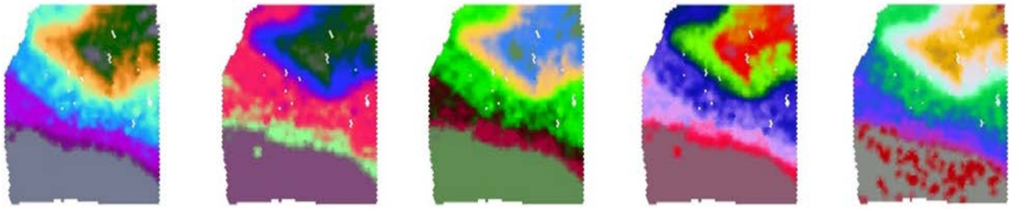

(F) First graph embedding. (G) Second graph embedding. (H) Third graph embedding. (I) Fourth graph embedding. (J) Fifth graph embedding.

1.1.14 2-8

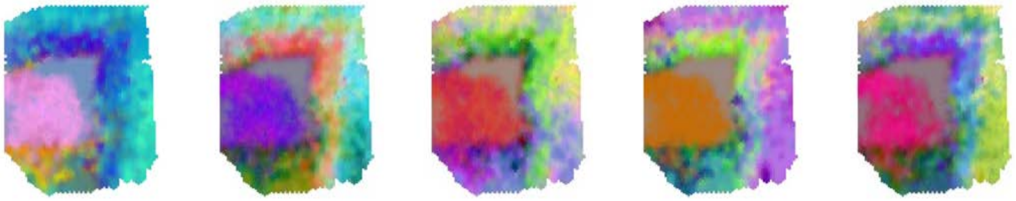

(A) First native embedding. (B) Second native embedding. (C) Third native embedding. (D) Fourth native embedding. (E) Fifth native embedding.

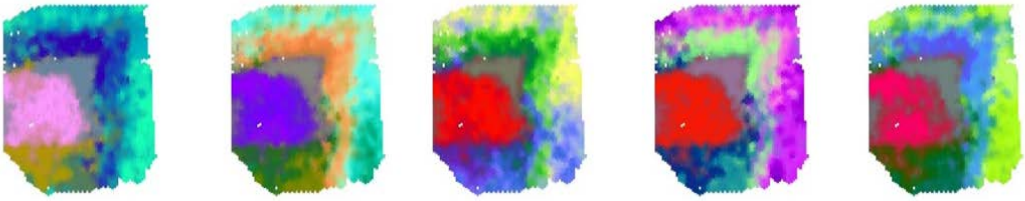

(F) First graph embedding. (G) Second graph embedding. (H) Third graph embedding. (I) Fourth graph embedding. (J) Fifth graph embedding.

1.1.15 18-64

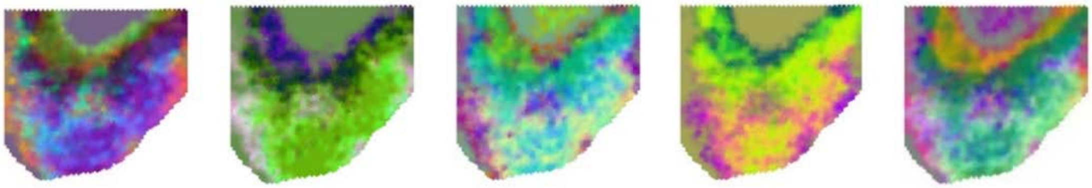

(A) First native embedding. (B) Second native embedding. (C) Third native embedding. (D) Fourth native embedding. (E) Fifth native embedding.

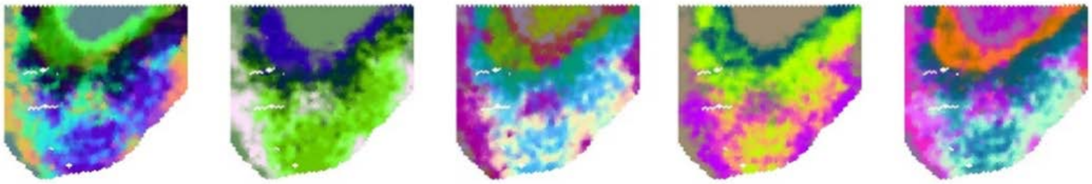

(F) First graph embedding. (G) Second graph embedding. (H) Third graph embedding. (I) Fourth graph embedding. (J) Fifth graph embedding.

1.1.16 T4857

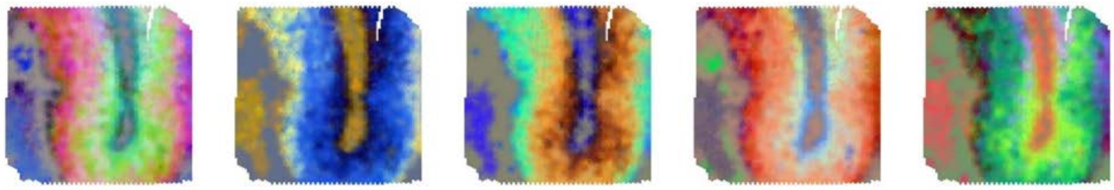

(A) First native embedding. (B) Second native embedding. (C) Third native embedding. (D) Fourth native embedding. (E) Fifth native embedding.

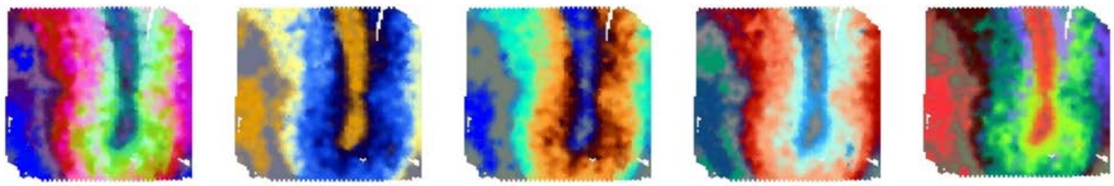

(F) First graph embedding. (G) Second graph embedding. (H) Third graph embedding. (I) Fourth graph embedding. (J) Fifth graph embedding.

Supplemental Figure S1. The RGB images of top 5 embeddings ranked by MP-MIM for 16 samples. From **(A)** to **(E)** in the first row, they are the RGB images based on the original embeddings of the top 5 output ranked by MP-MIM. From **(F)** to **(J)** in the second row, they are the RGB images generated by graph embeddings transformed from the original embeddings in the first row.

1.2 Supplemental Figure S2

1.2.1 151507, 151508, 151509, and 151669

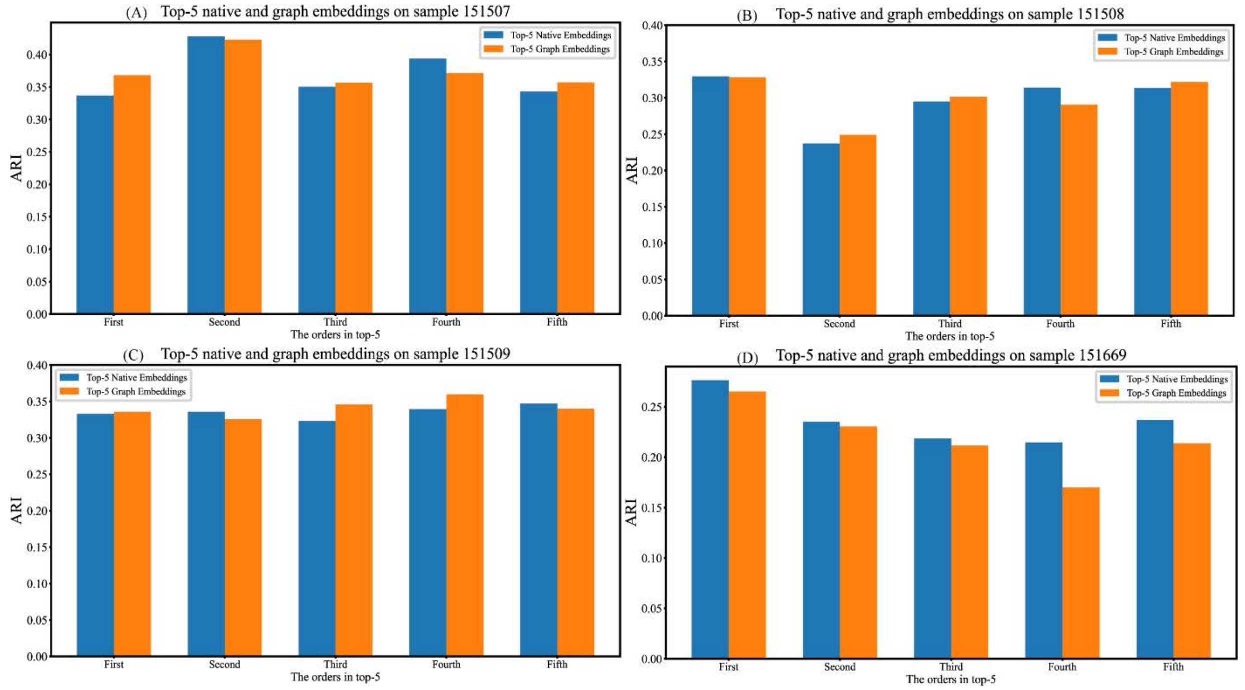

1.2.2 151510, 151672, 151674, and 18-64

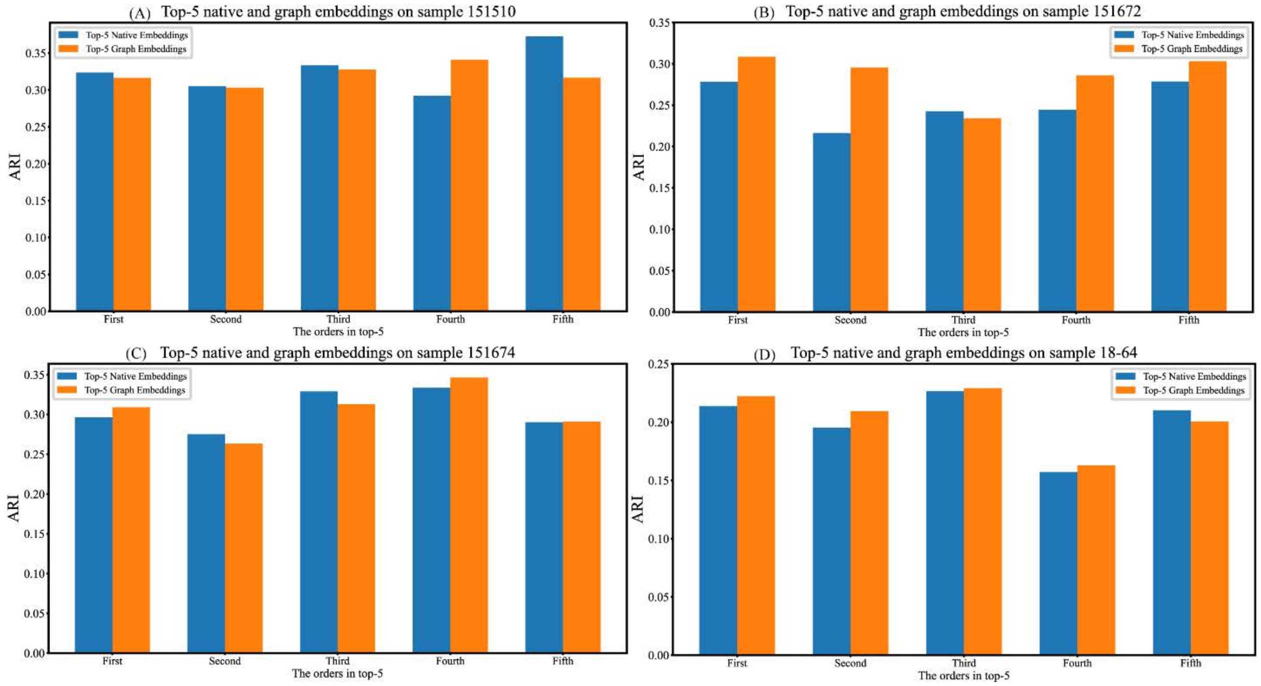

1.2.3 151670, 151671, 151673 and 151675

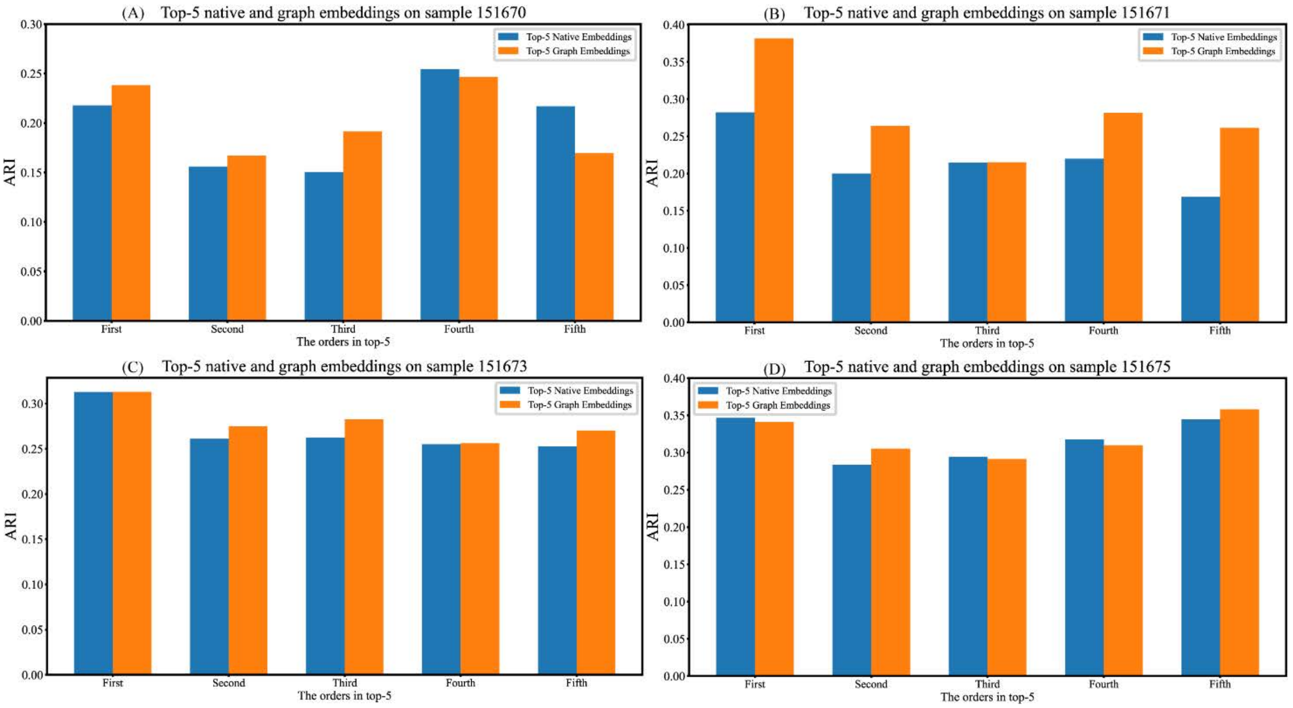

1.2.4 151676, 2-5, 2-8 and T4857

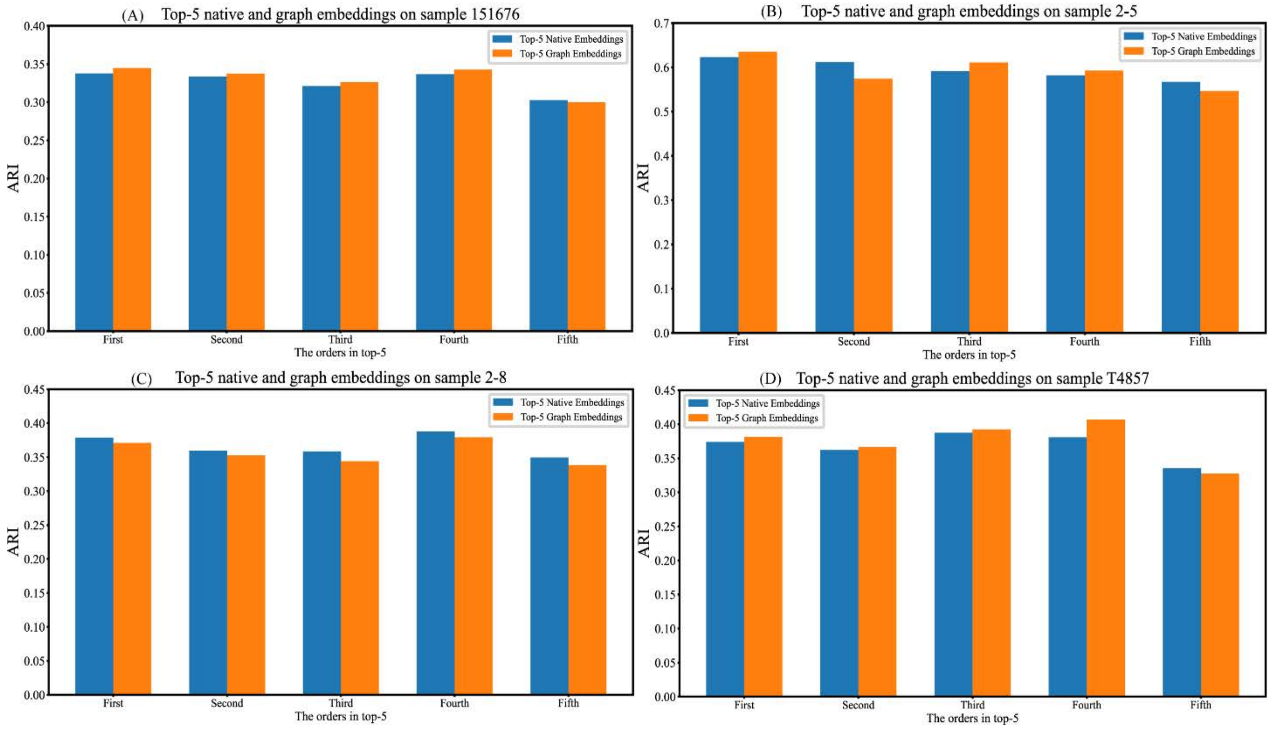

Supplemental Figure S2. Comparison ARI results between the top 5 original embeddings and the corresponding transformed embeddings on 16 samples. The horizontal axis represents the specific orders of the embeddings among the top 5. The vertical axis represents the ARI between the ground truth labels and the labels of K-means.

1.3 Supplemental Figure S3

1.3.1 151507, 151510, 151669, 151670, 151671 and 151672

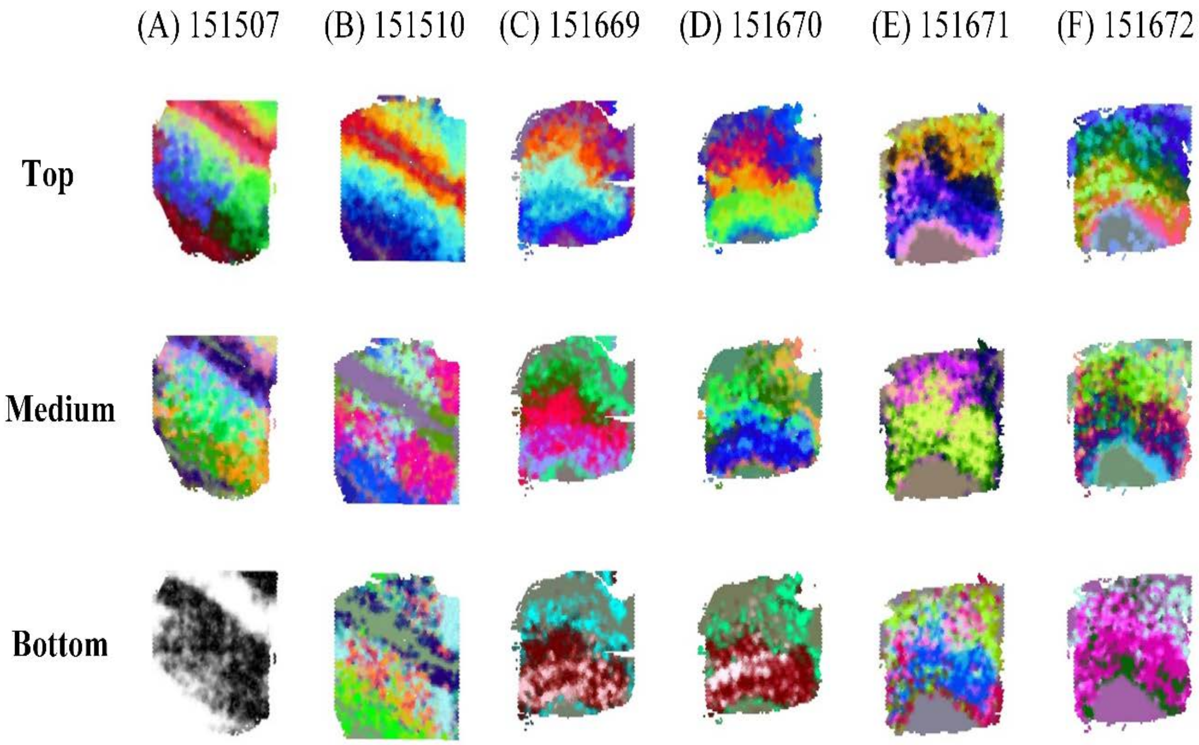

1.3.2 151676, 2-5, 2-8, 18-64 and T4857

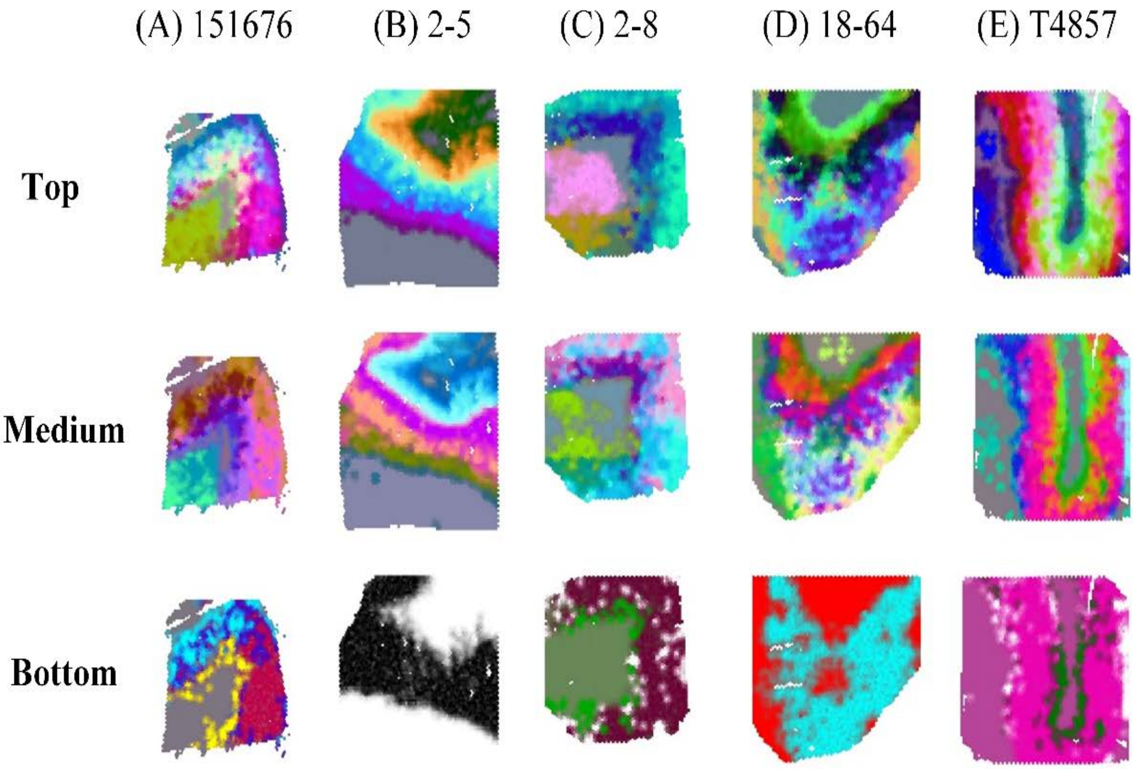

Supplemental Figure S3. Comparison results of the top, medium, and bottom-ranked RGB images based on transformed embeddings in MP-MIM rankings on 11 samples.

1.4 Supplemental Figure S4

1.4.1 151507, 151508, 151509, and 151669

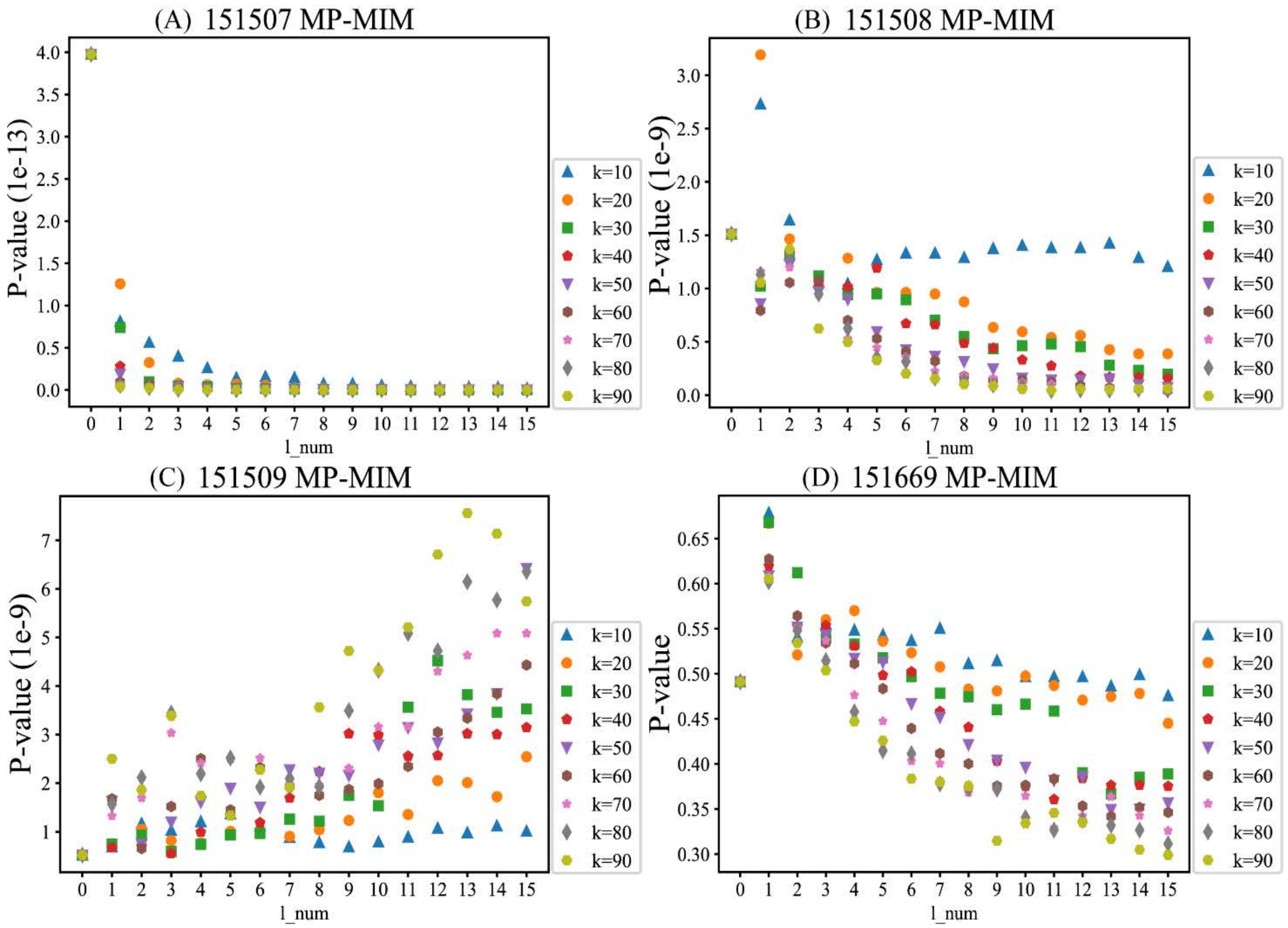

1.4.2 151510, 151672, 151674, and 18-64

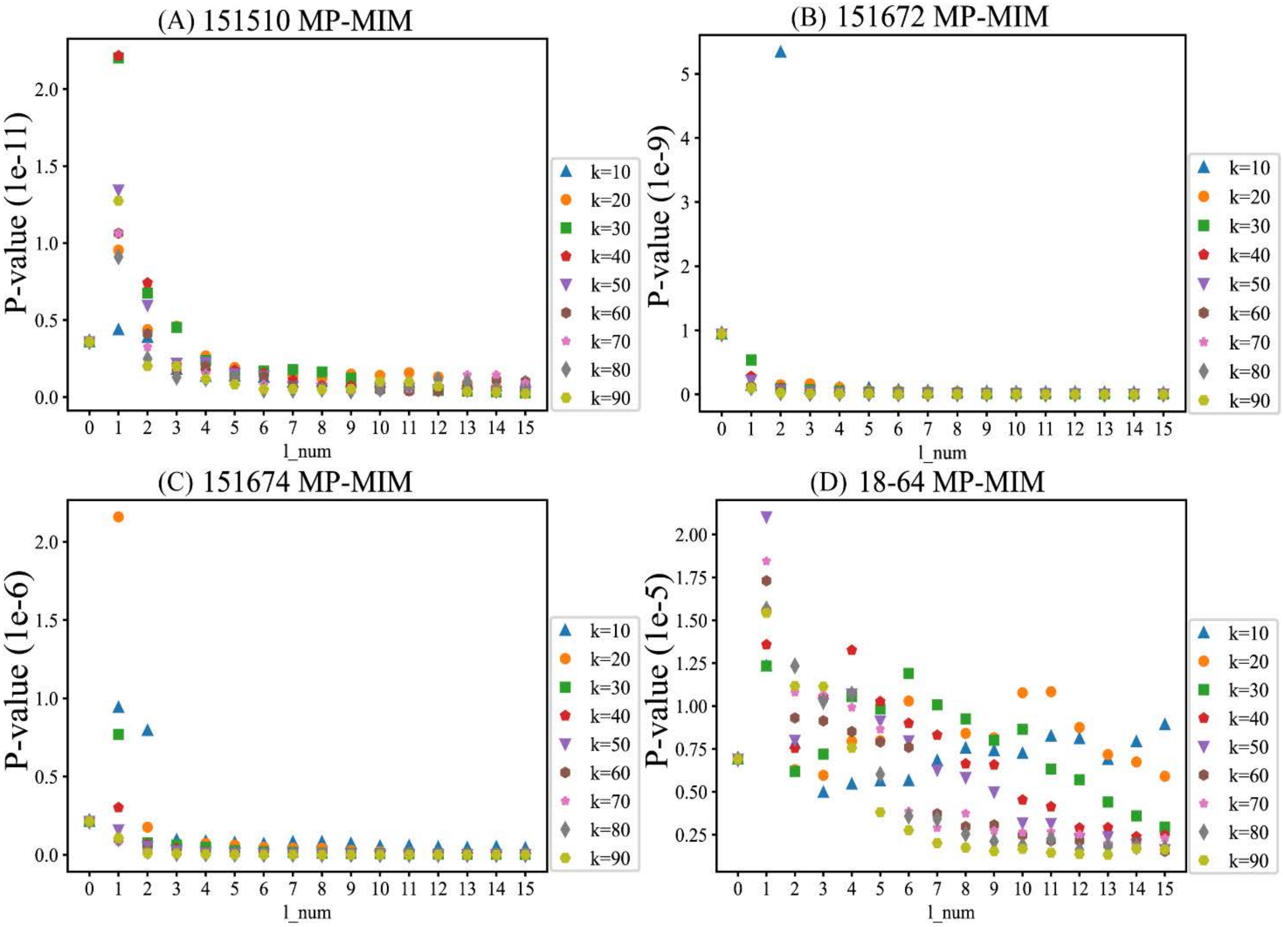

1.4.3 151670, 151671, 151673, and 151675

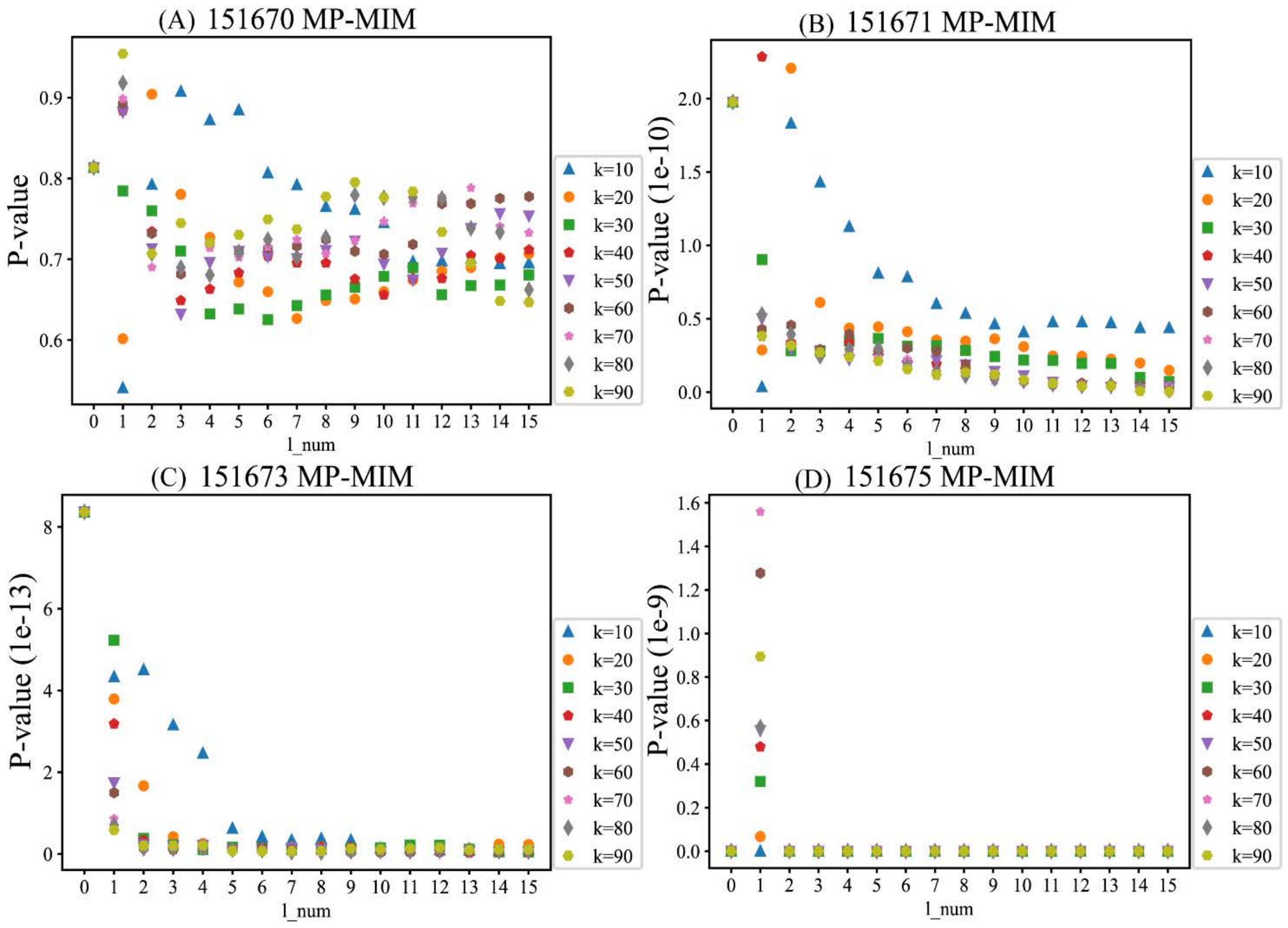

1.4.4 151676, 2-5, 2-8, and T4857

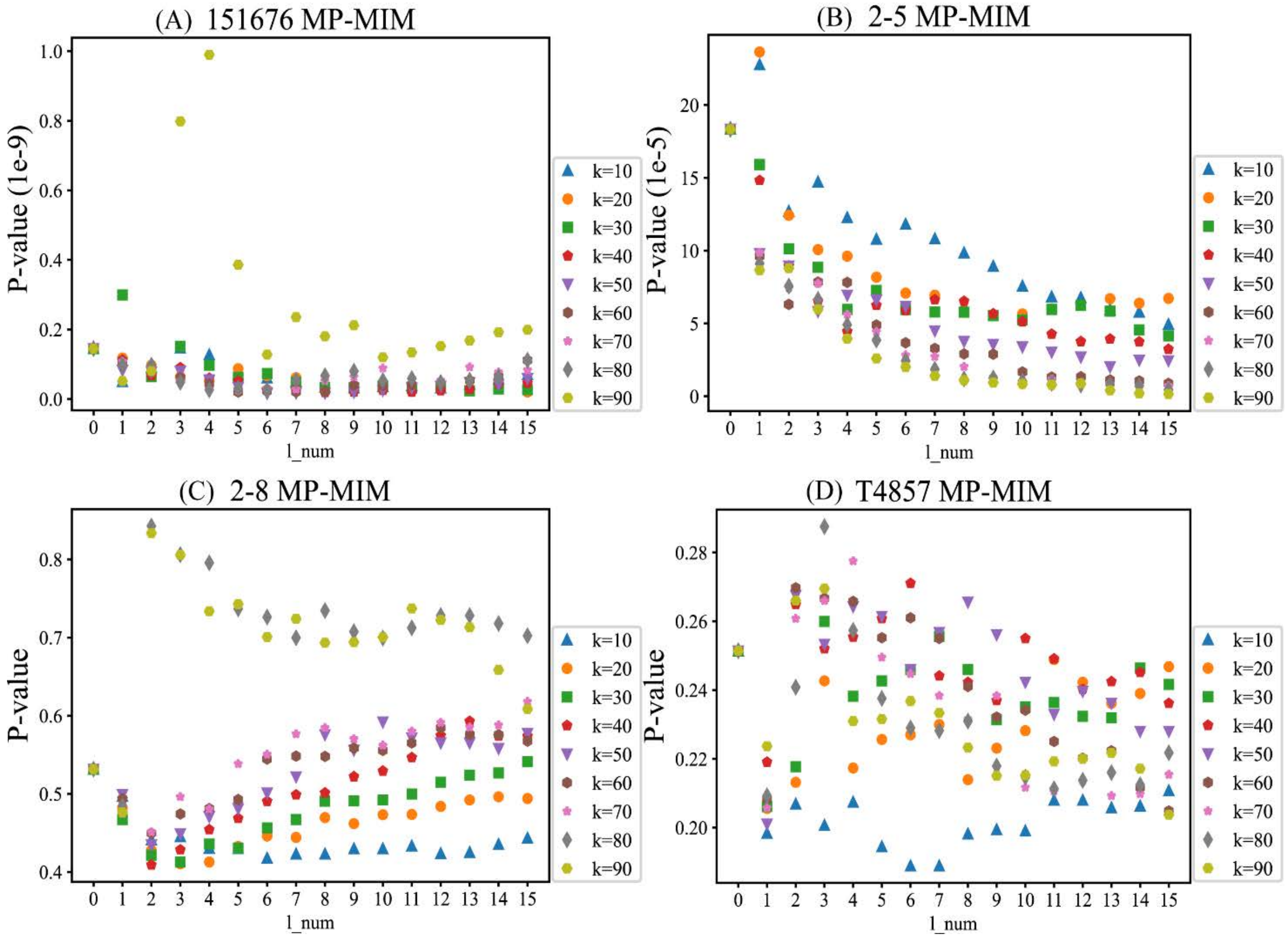

Supplemental Figure S4. *P value* on the hyperparameters *k_num* and *l_num* in message passing to the Spearman correlation on 12 samples. The horizontal axis represents the number of layers in the message passing process. The vertical axis represents the P-value of Spearman correlation measured by the ranking of MP-MIM and the ground truth. *k* is the number of nearest neighbors, which is the predefined parameter in the KNN graph.

1.5 Supplemental Figure S5

1.5.1 151507, 151508, 151509, and 151669

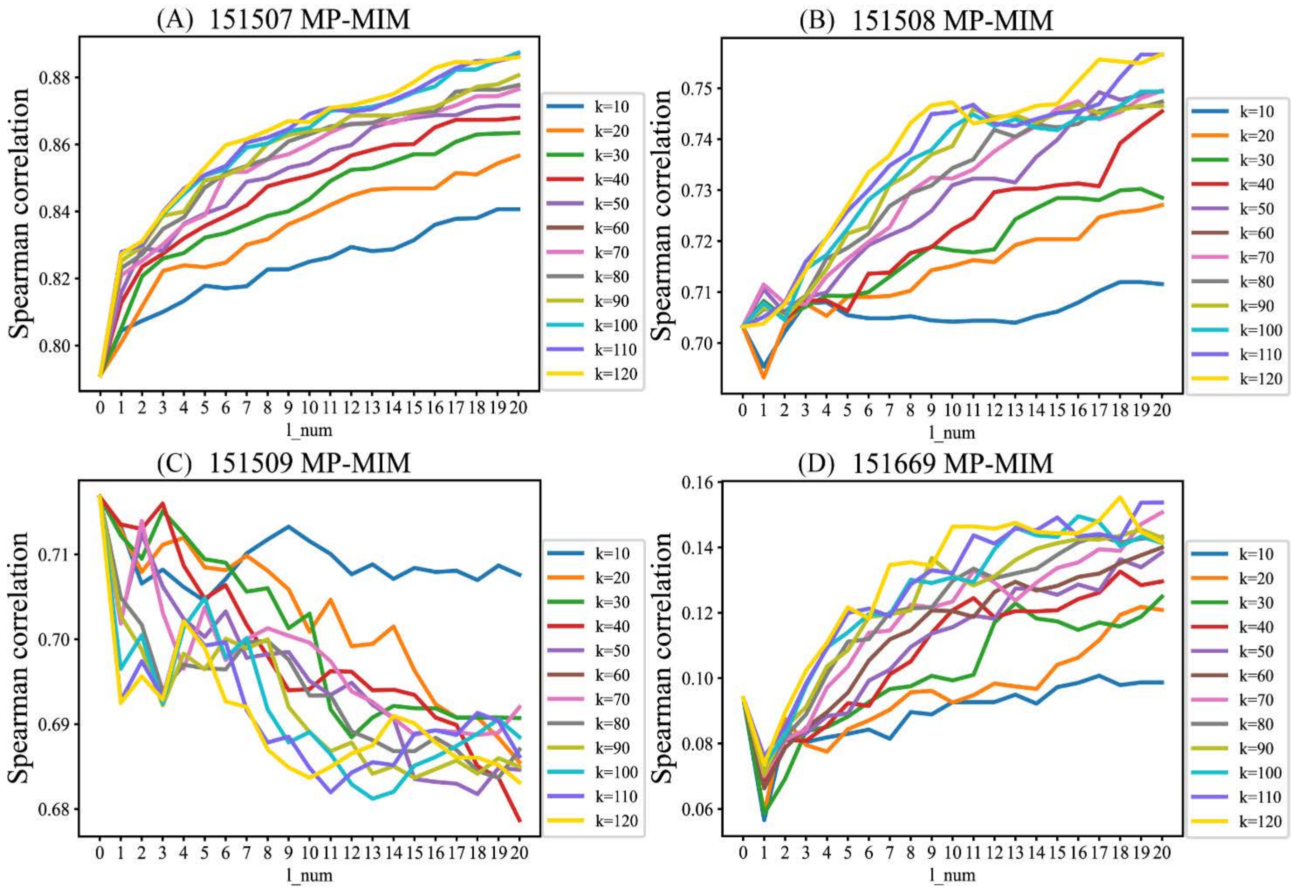

1.5.2 151510, 151672, 151674, and 18-64

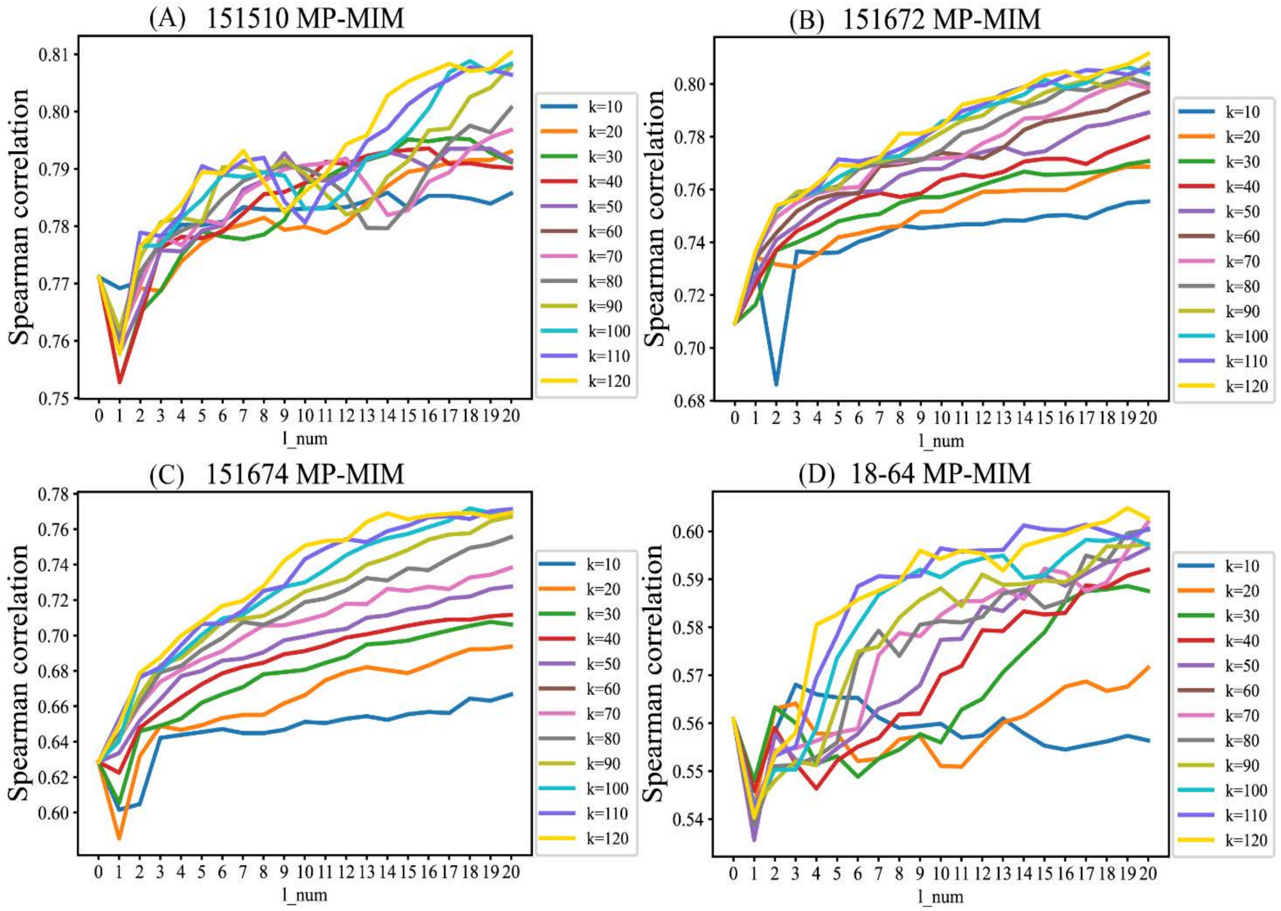

1.5.3 151670, 151671, 151673, and 151675

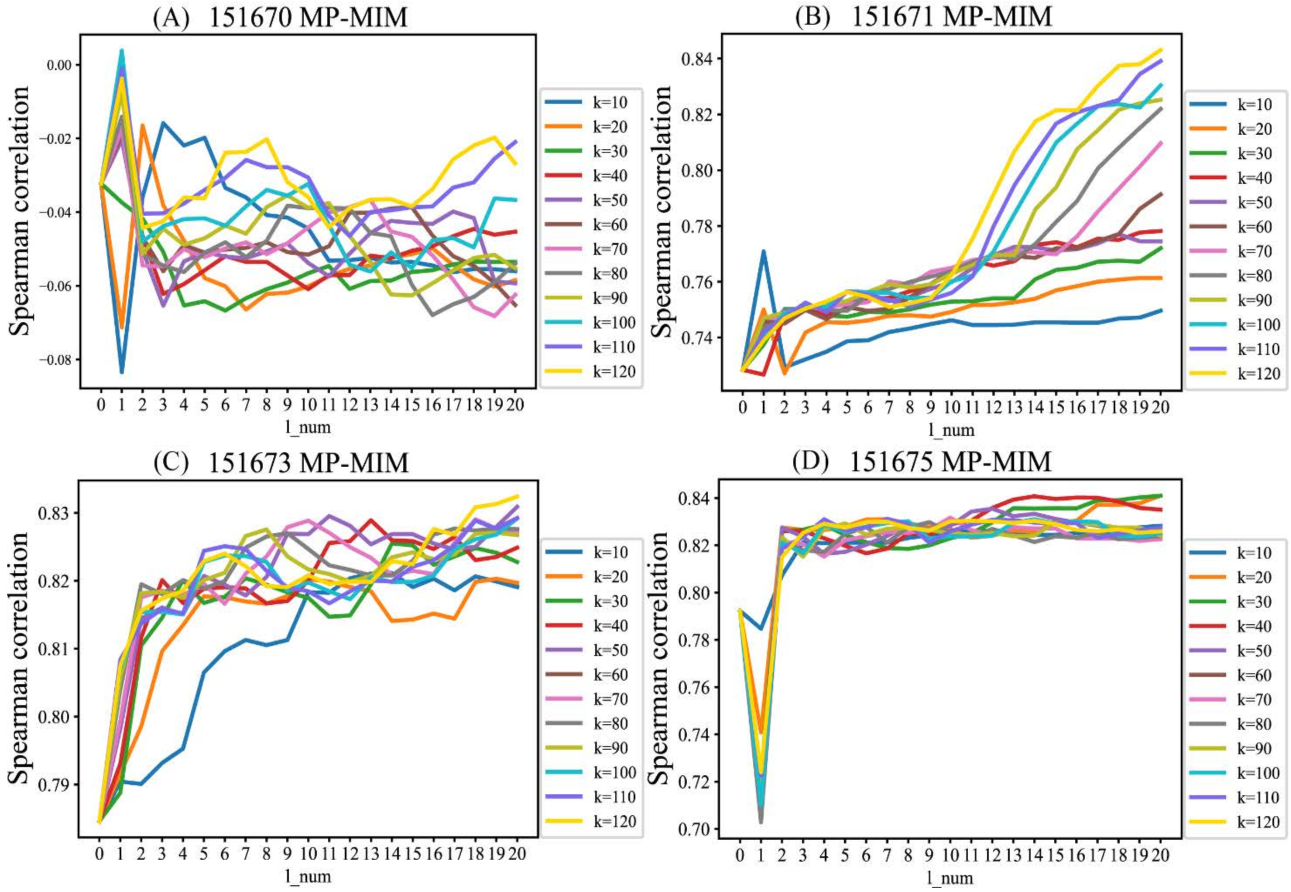

1.5.4 151676, 2-5, 2-8, and T4857

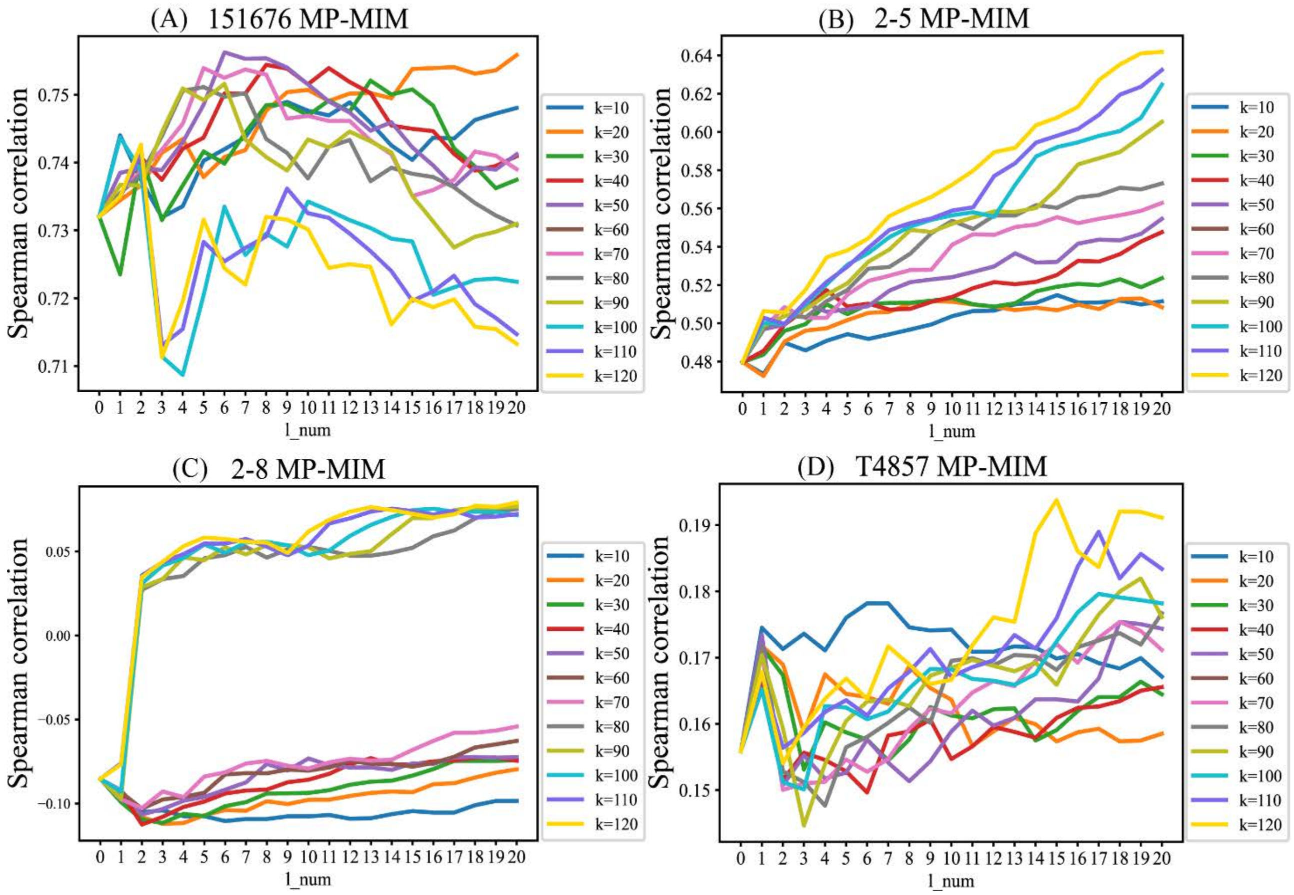

Supplemental Figure S5. Sensitivities of more settings of the hyperparameters *k_num* and *l_num* in message passing to the Spearman correlation on 16 samples. The horizontal axis represents the number of layers in the message passing. The vertical axis represents the Spearman correlation measured by the ground-truth ranking and the ranking of the MP-MIM. The value range of *k_num* is from 10 to 120, and the value range *l_num* is from 1 to 20. *k* denotes the number of nearest neighbors, which is the predefined parameter in the KNN graph.

1.6 Supplemental Figure S6

1.6.1 151507, 151508, 151509, and 151669

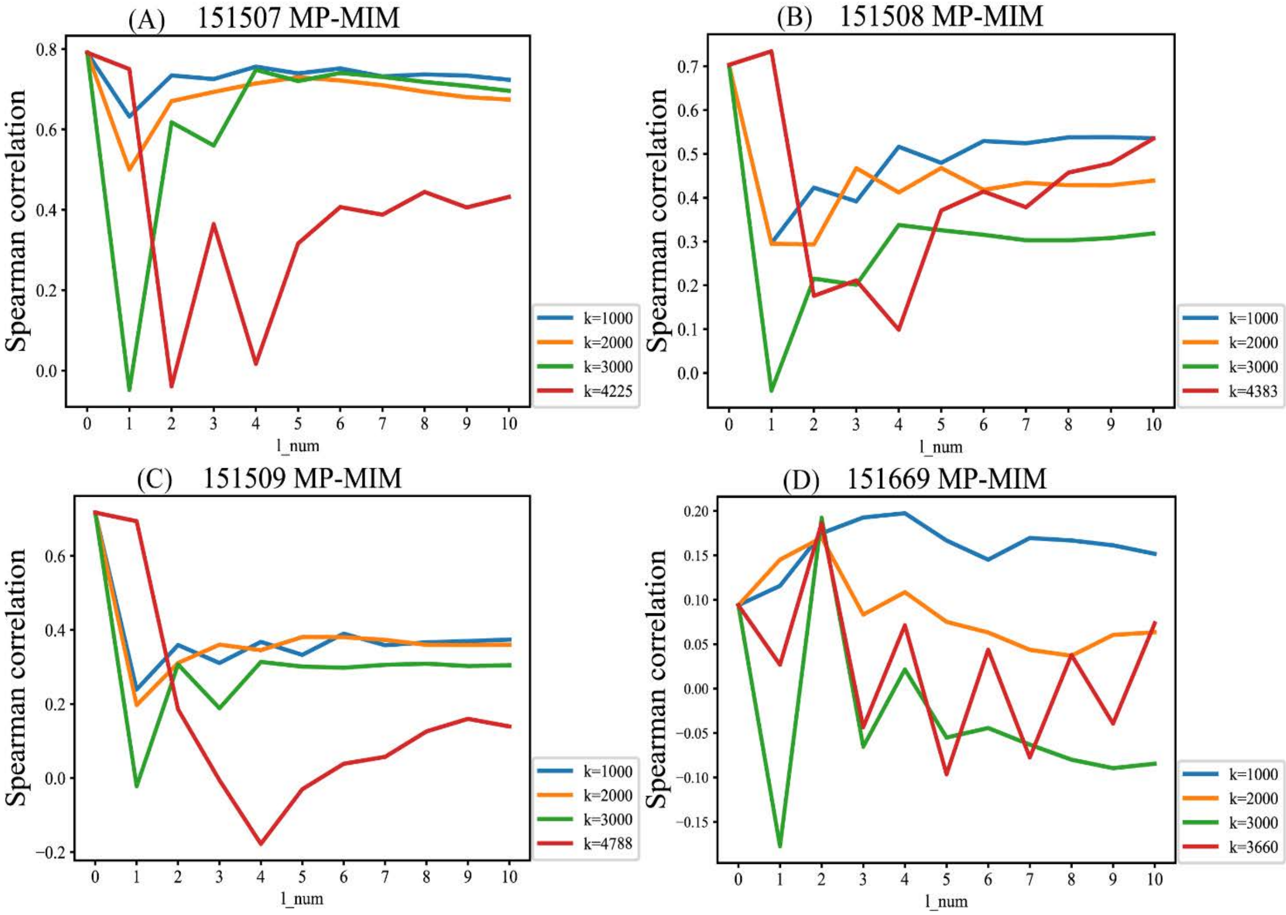

1.6.2 151510, 151672, 151674, and 18-64

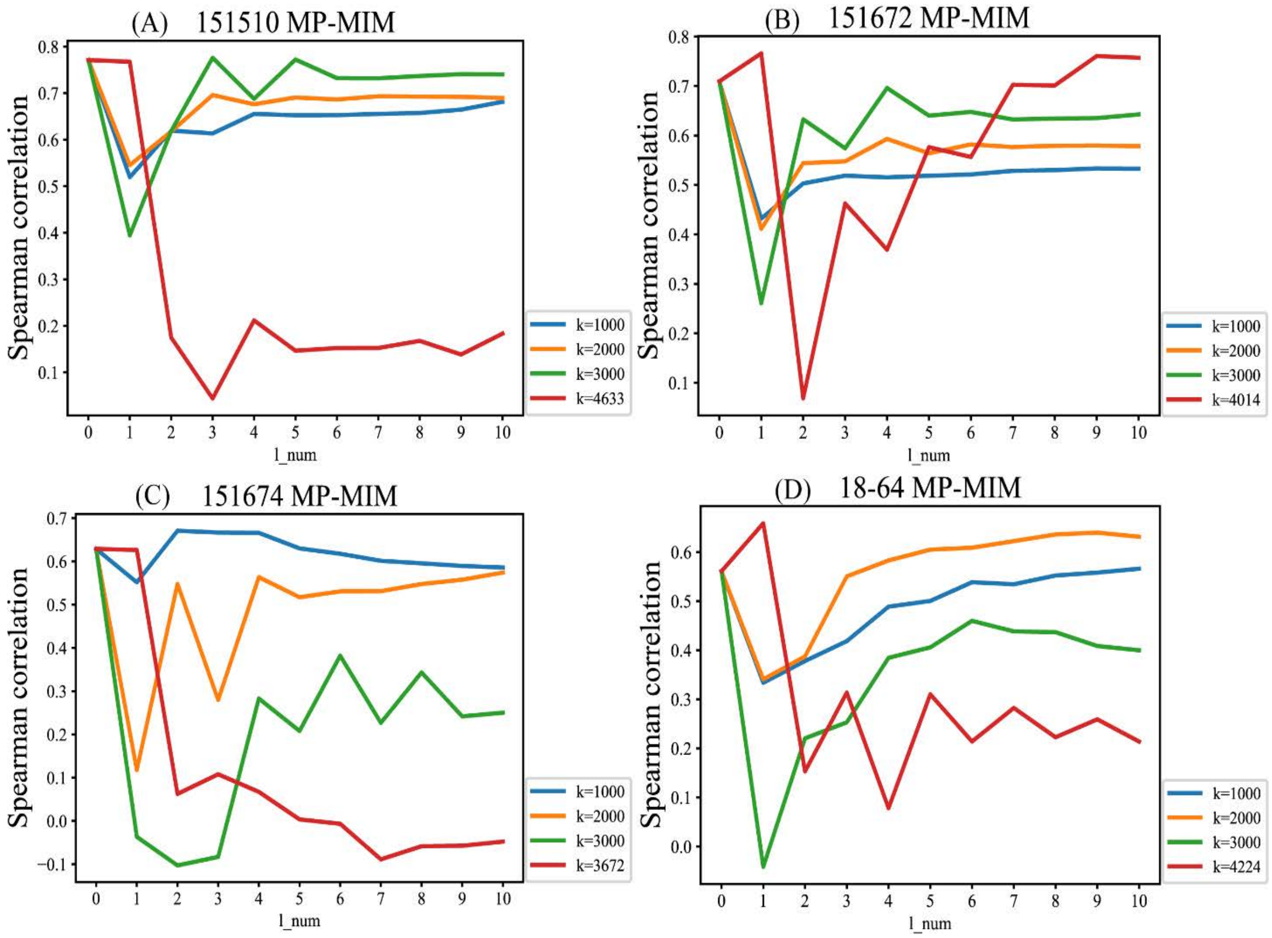

1.6.3 151670, 151671, 151673, and 151675

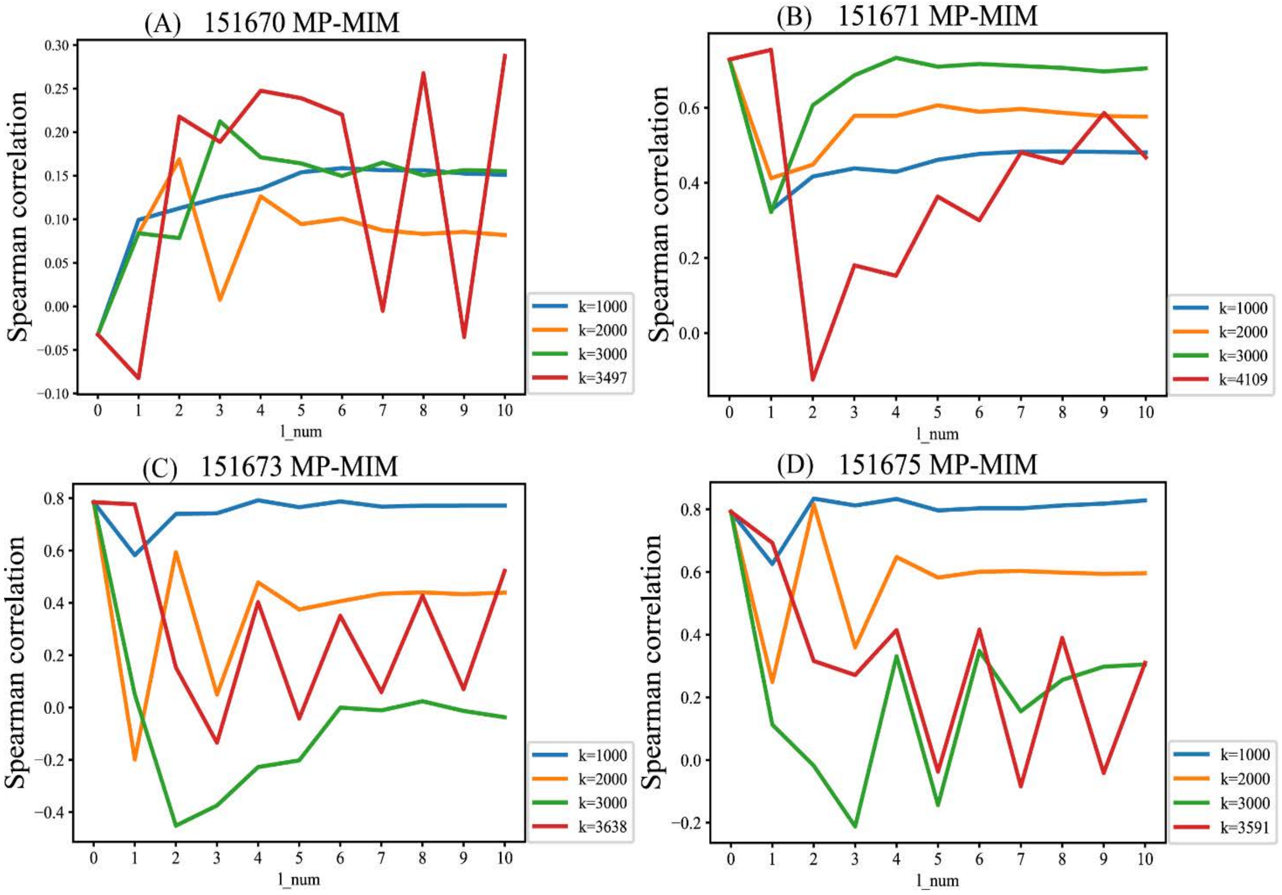

1.6.4 151676, 2-5, 2-8, and T4857

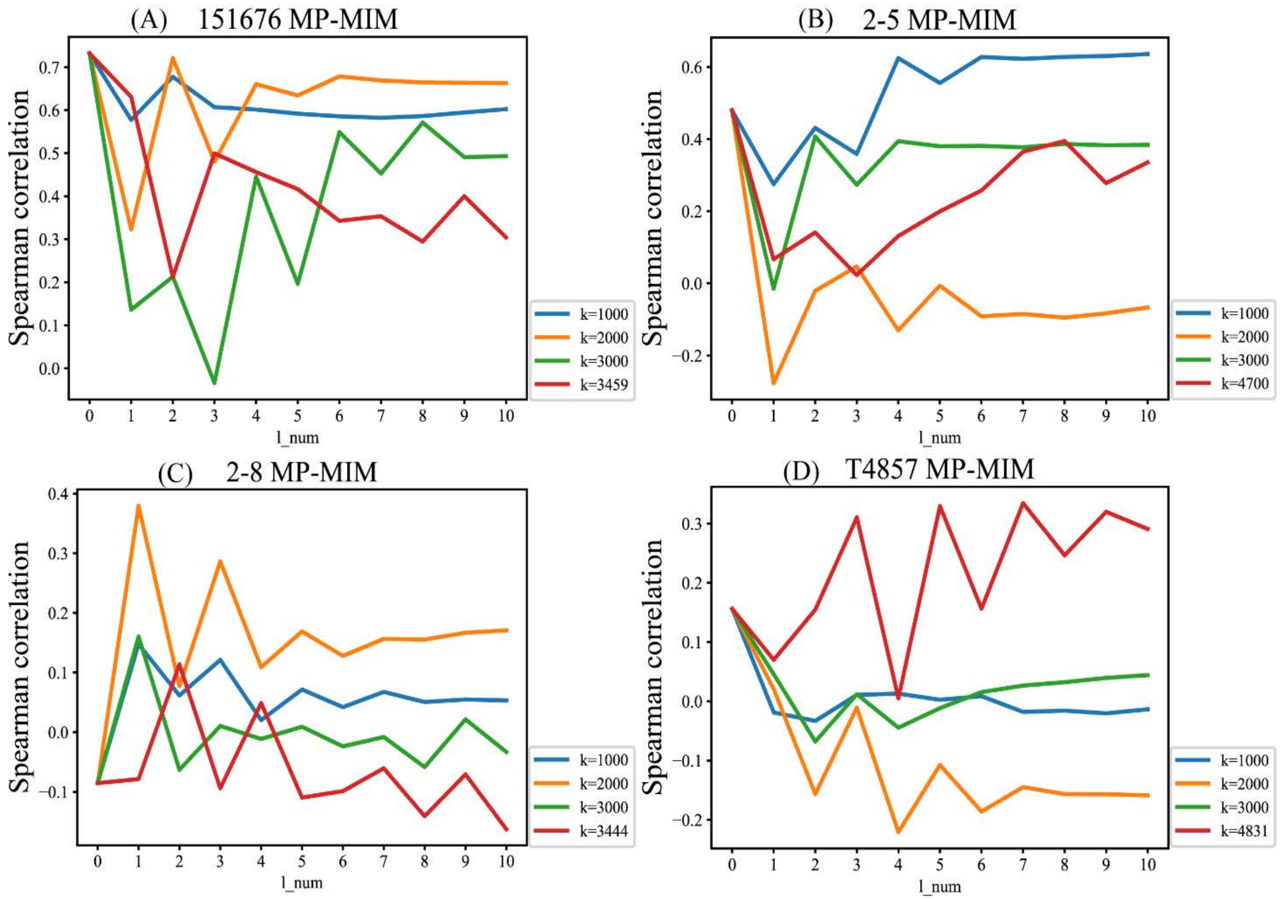

Supplemental Figure S6. Sensitivities of more settings of the hyperparameters *k_num* and *l_num* in message passing to the Spearman correlation on 16 samples. The horizontal axis represents the number of layers in the message passing. The vertical axis represents the Spearman correlation measured by the ground-truth ranking and the ranking of the MP-MIM. The considered values of *k_num* are 1000, 2000, 3000, and the maximum number of neighbors and the value range *l_num* is from 1 to 10. *k* denotes the number of nearest neighbors, which is the predefined parameter in the KNN graph.

1.7 Supplemental Figure S7

1.7.1 151507, 151508, 151509, and 151669

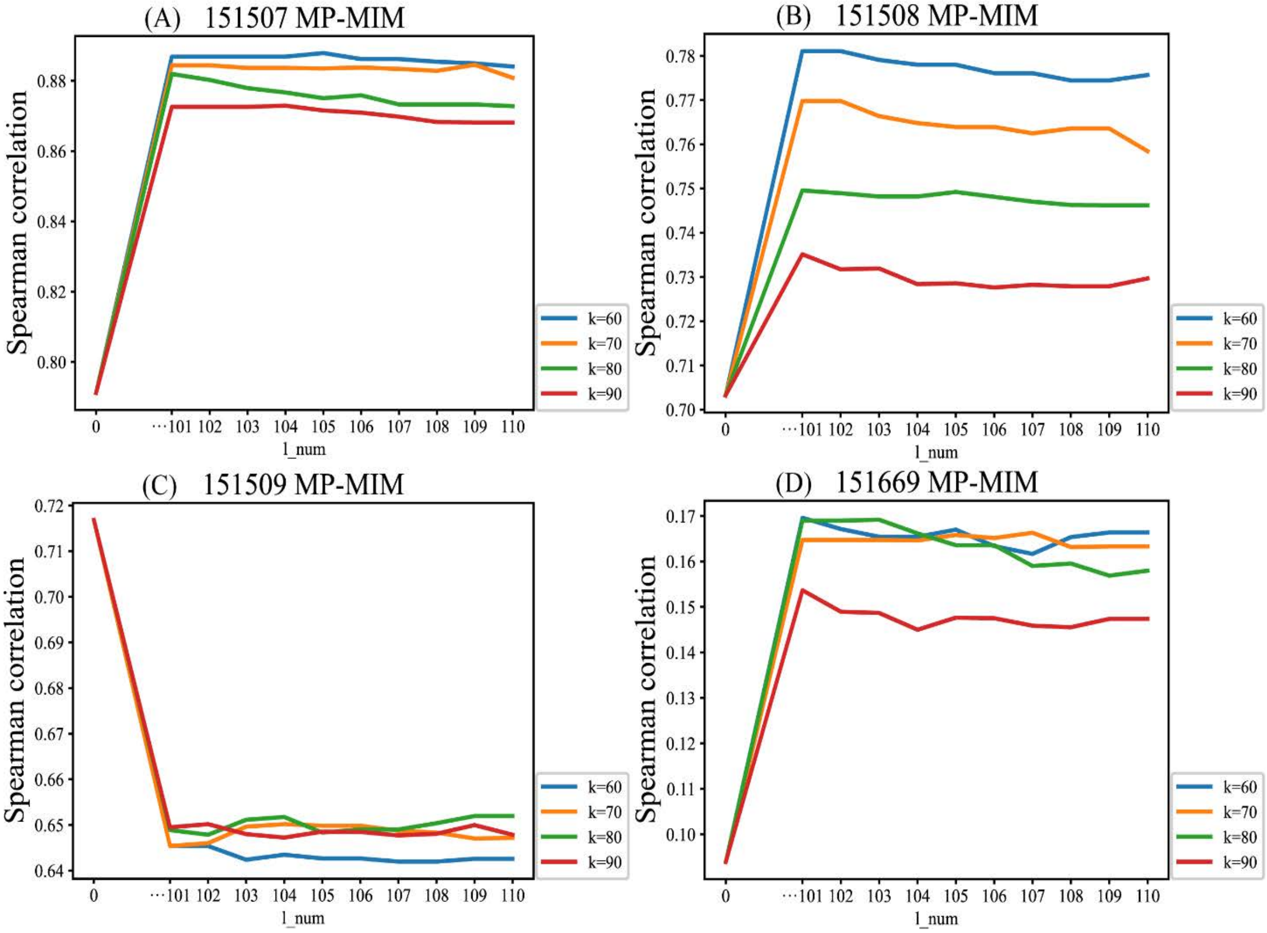

1.7.2 151510, 151672, 151674, and 18-64

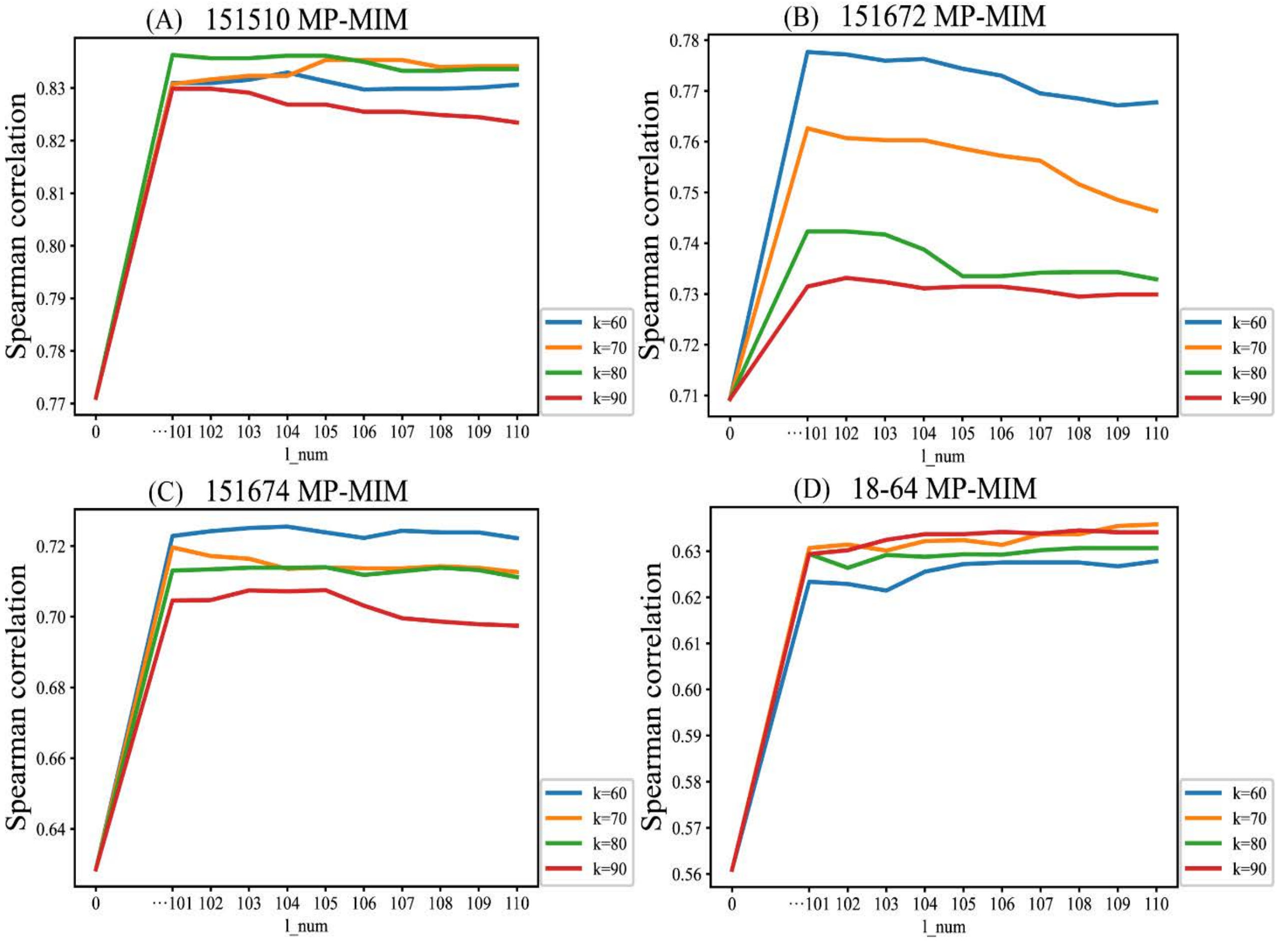

1.7.3 151670, 151671, 151673, and 151675

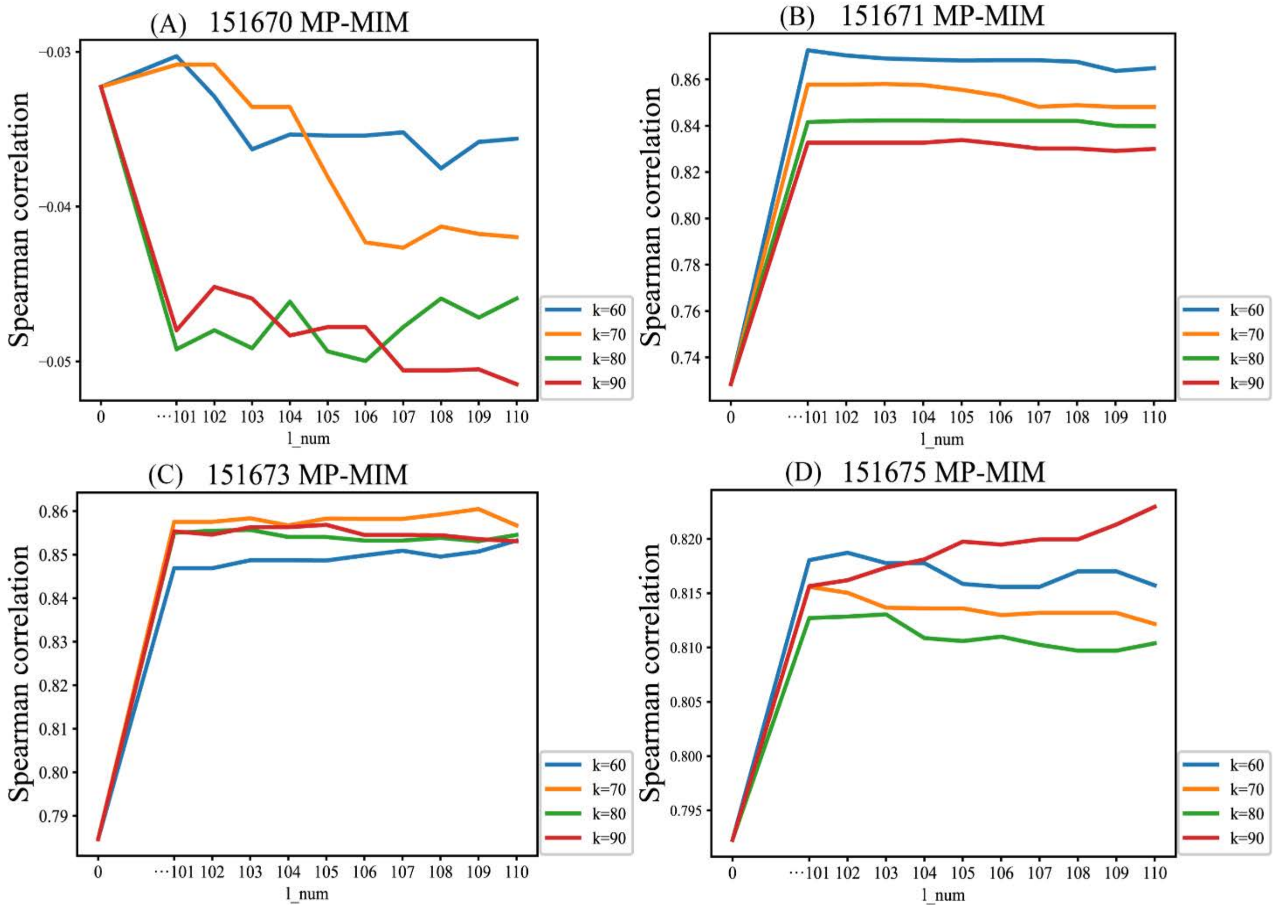

1.7.4 151676, 2-5, 2-8, and T4857

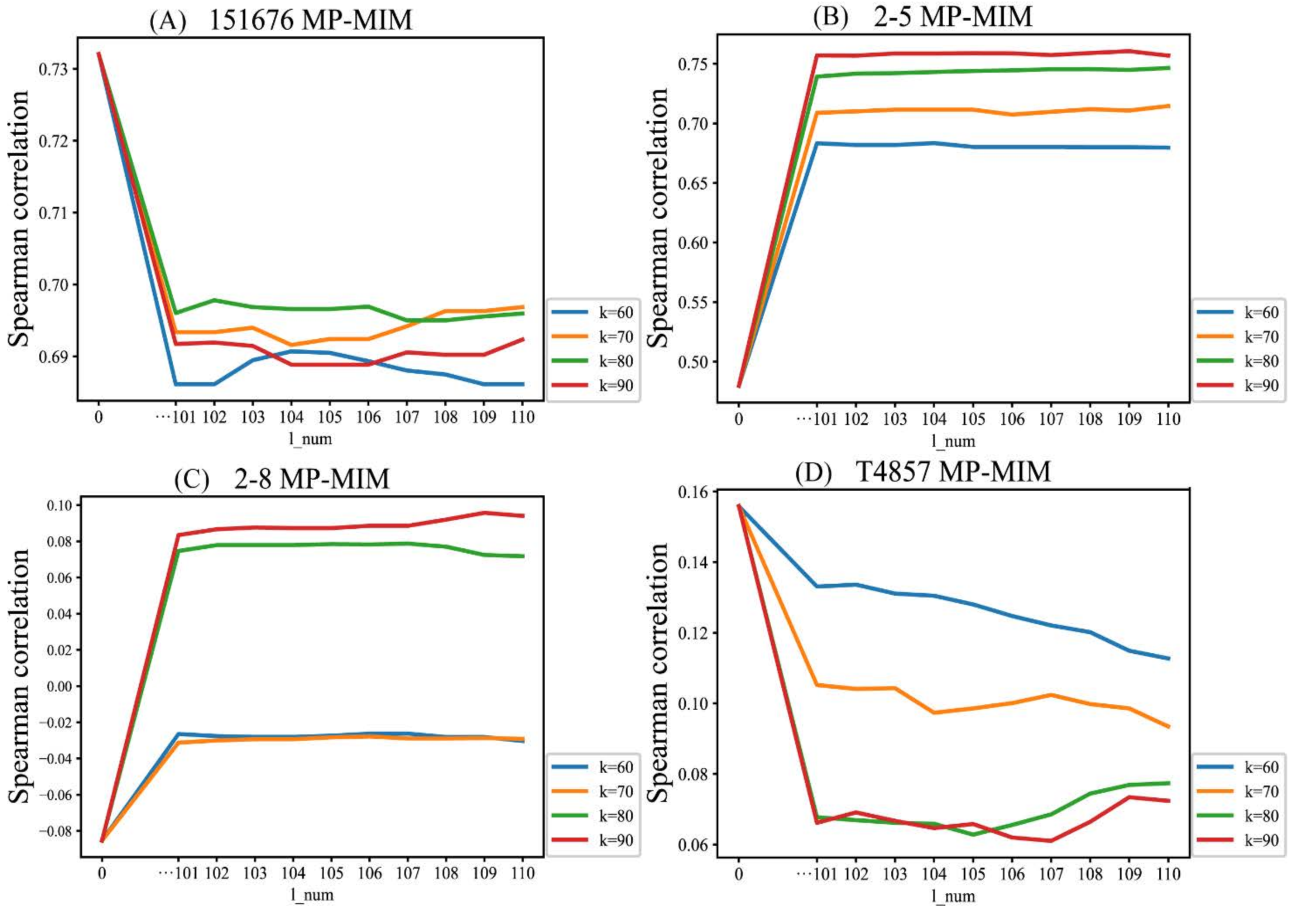

Supplemental Figure S7. Sensitivities of more settings of the hyperparameters *k_num* and *l_num* in message passing to the Spearman correlation on 16 samples. The horizontal axis represents the number of layers in the message passing. The vertical axis represents the Spearman correlation measured by the ground-truth ranking and the ranking of the MP-MIM. The considered values of *k_num* are 60, 70, 80, and 90, and the value range *l_num* is from 101 to 110. *k* denotes the number of nearest neighbors, which is the predefined parameter in the KNN graph.

1.8 Supplemental Figure S8

1.8.1 151507, 151508, 151509, and 151669

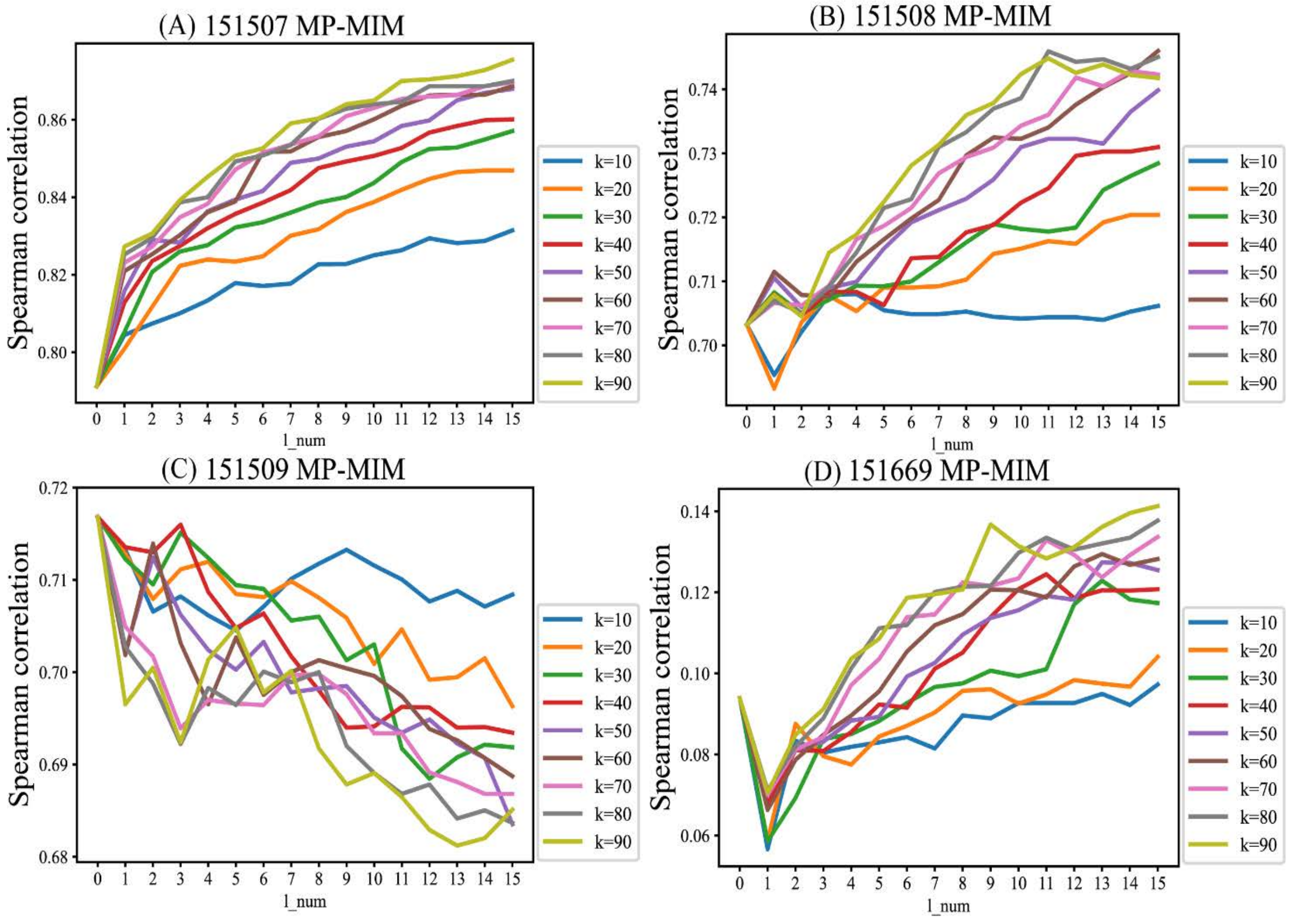

1.8.2 151670, 151671, 151673, and 151675

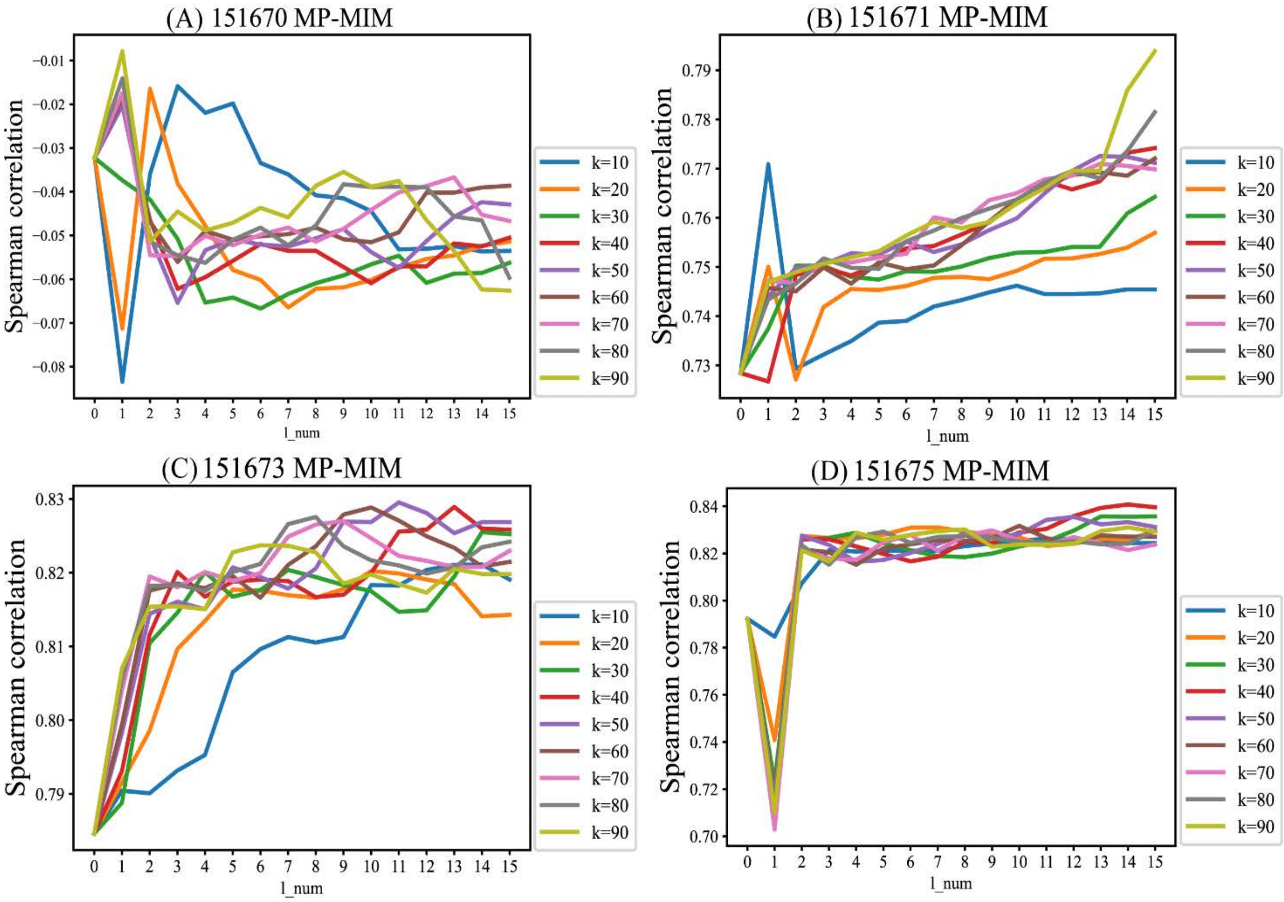

1.8.3 151676, 2-5, 2-8, and T4857

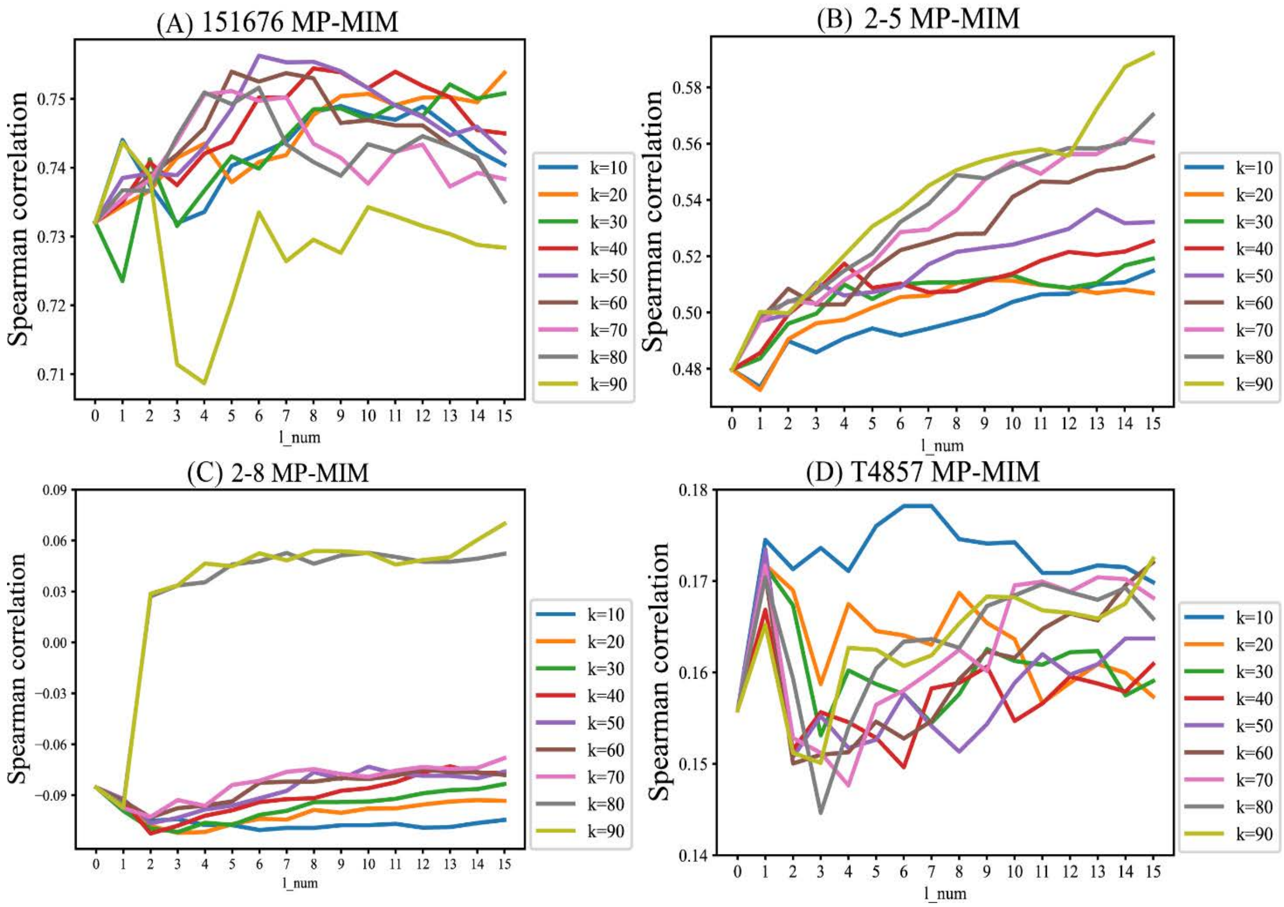

Supplemental Figure S8. Sensitivities of the hyperparameters *k_num* and *l_num* in message passing to the Spearman correlation on 12 samples. The horizontal axis represents the number of layers in the message passing. The vertical axis represents the Spearman correlation measured by the ground-truth ranking and the ranking of the MP-MIM. *k* denotes the number of nearest neighbors, which is the predefined parameter in the KNN graph.

1.9 Supplemental Figure S9

1.9.1 151507, 151508, 151509, and 151669

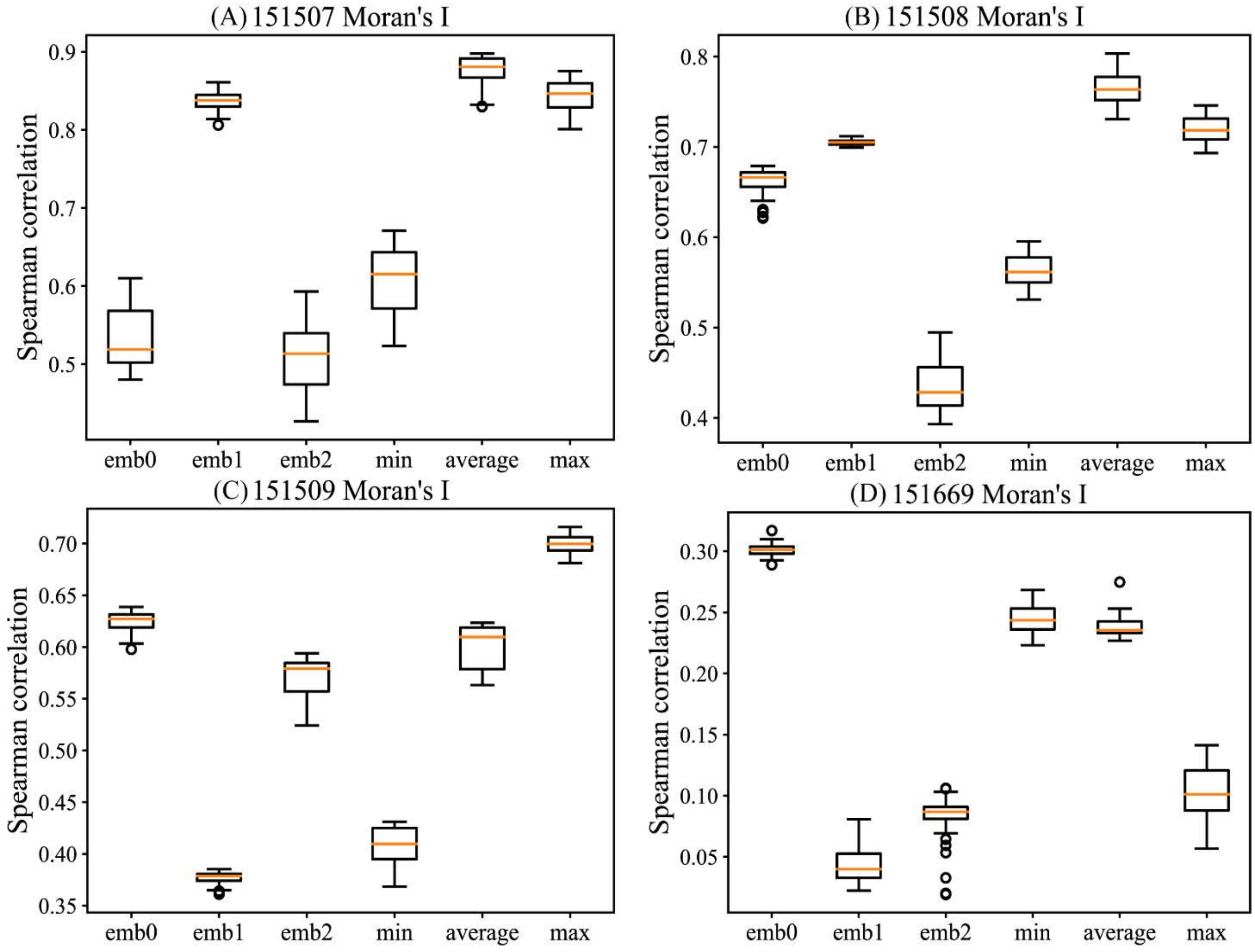

1.9.2 151670, 151671, 151673, and 151675

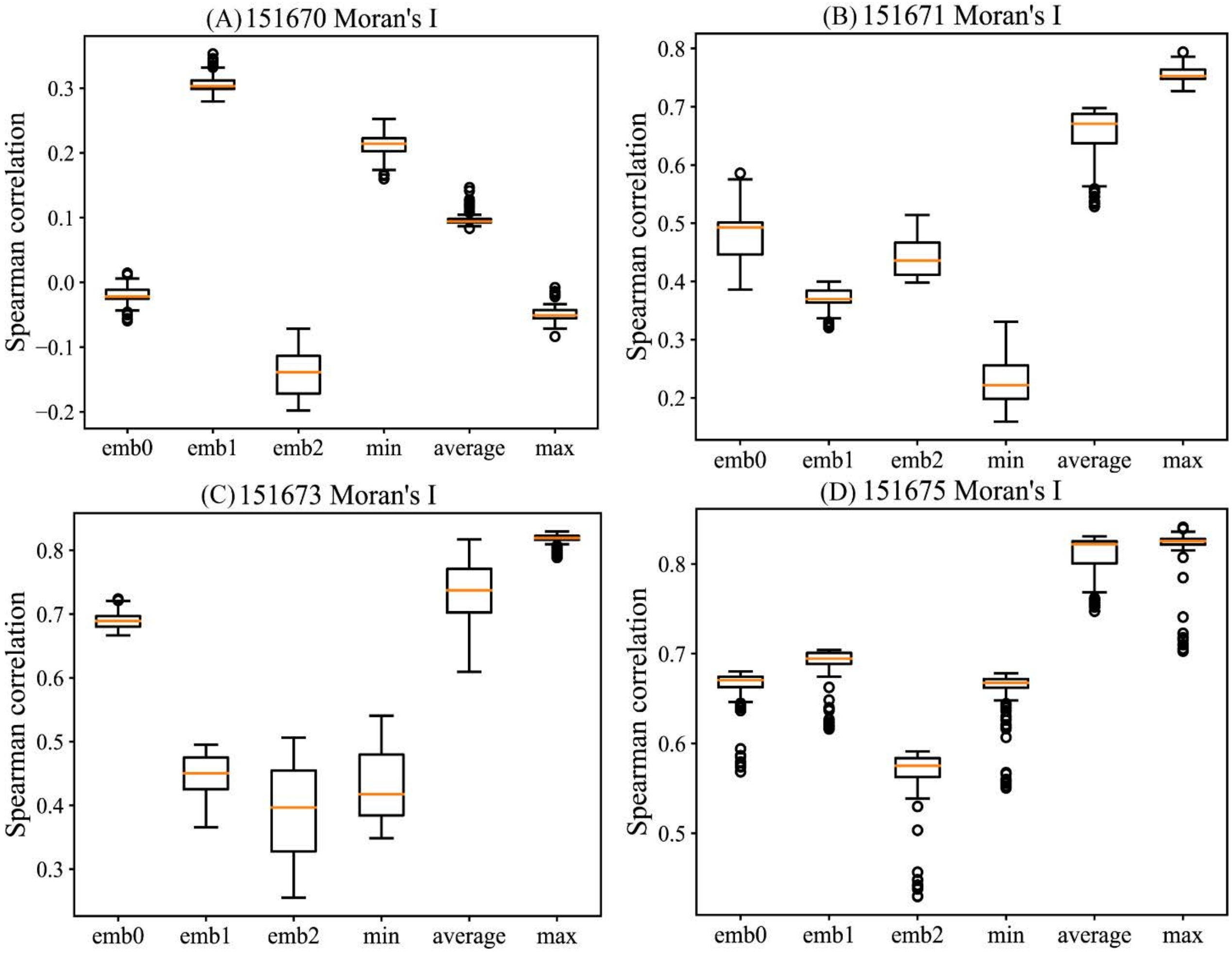

1.9.3 151676, 2-5, 2-8, and T4857

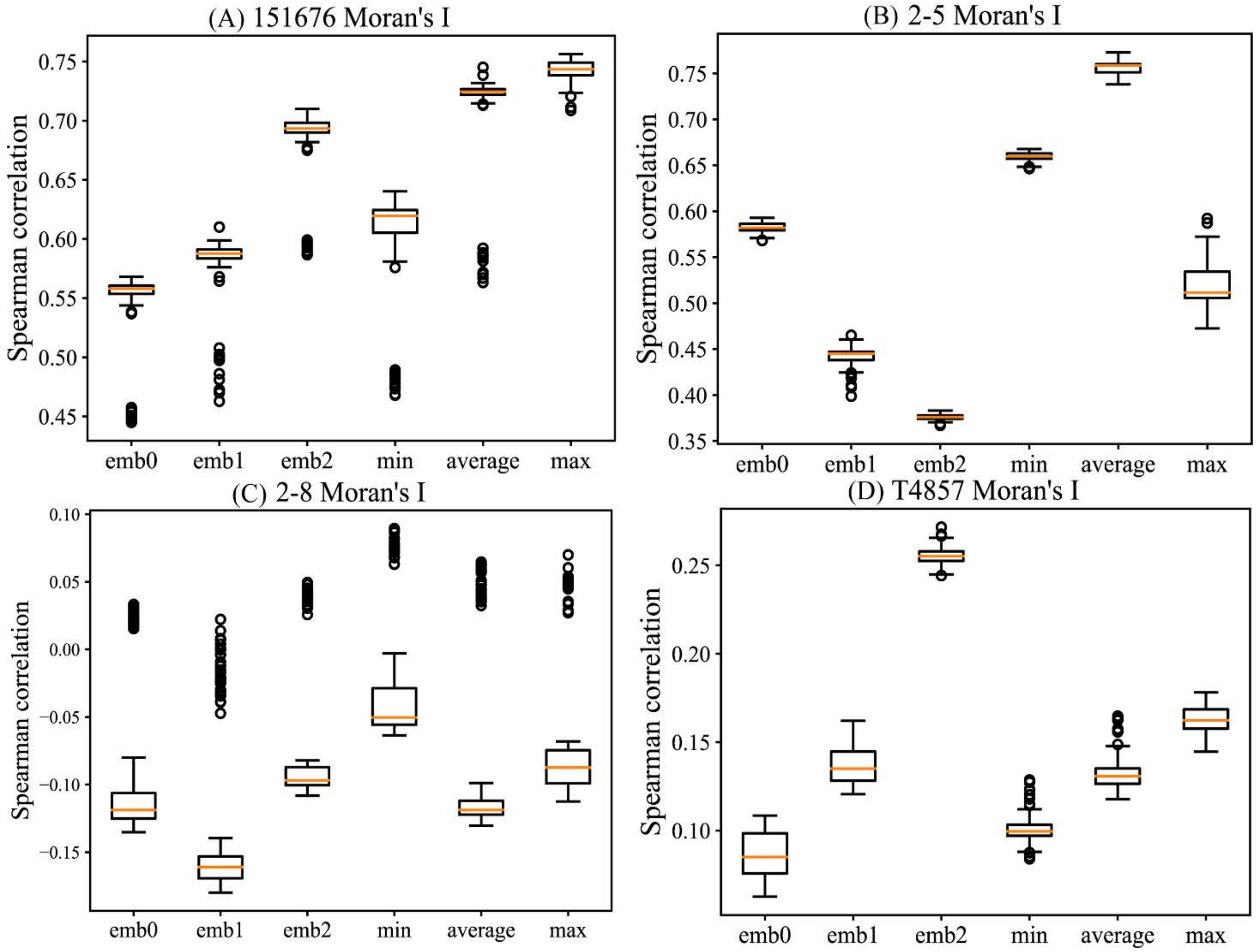

Supplemental Figure S9. Comparison among different methods to measure Moran's I by applying the message passing process on 12 samples. The horizontal axis shows the methods of directly measuring Moran's I on the first dimension (emb0), second dimension (emb1), and third dimension (emb2) in each embedding and the methods of combining Moran's I with the minimum, average and maximum filtering in all dimensions. The vertical axis shows the Spearman correlation measured by the ground-truth ranking and the ranking by MP-MIM. Each box shows all results in message passing with *k_num* values from 10 to 90 and *l_num* values from 1 to 15.

1.10 Supplemental Figure S10

1.10.1 151507, 151508, 151509, 151510, 18-64, and T4857

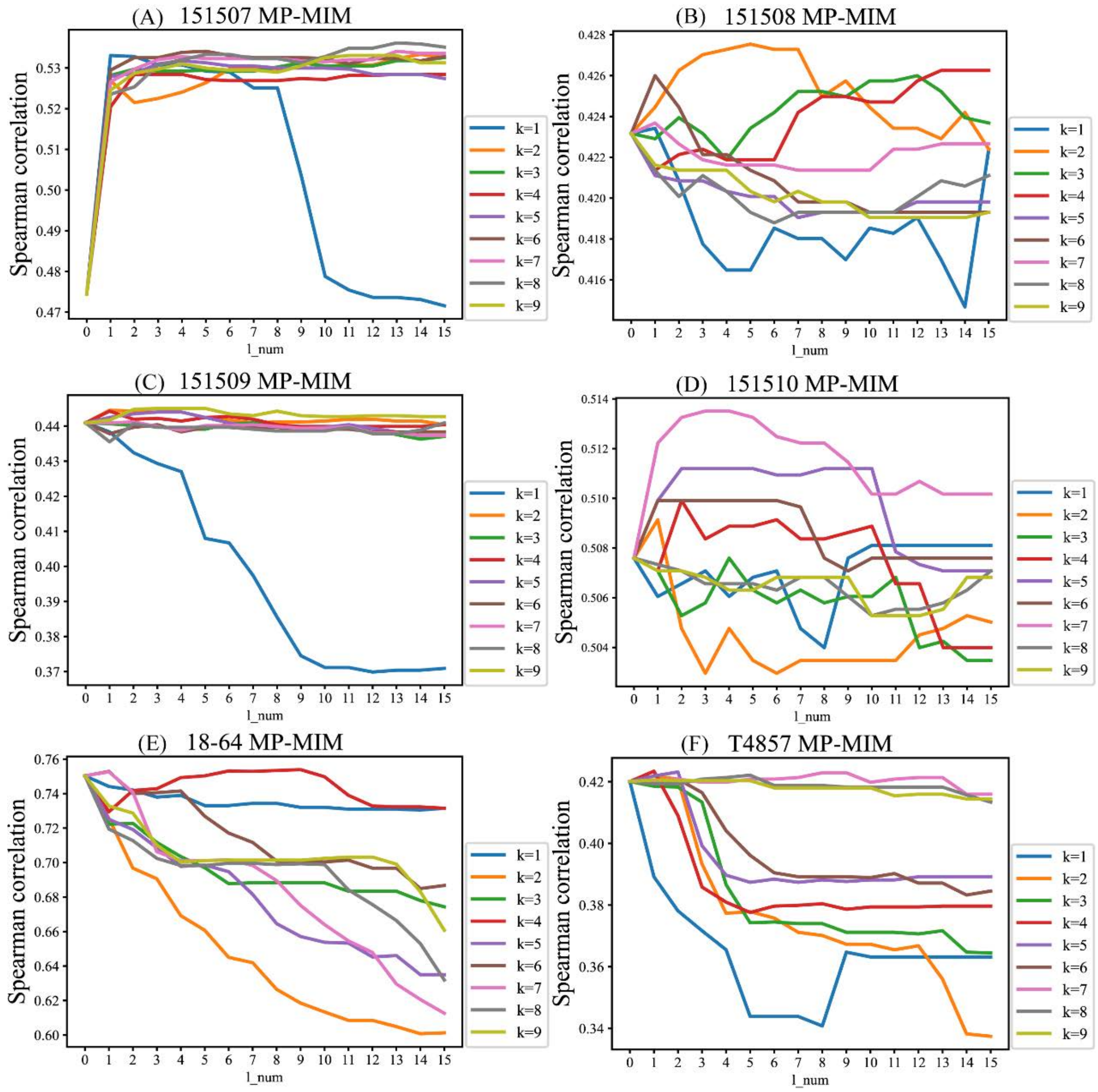

Supplemental Figure S10. Sensitivity of the hyperparameters *k_num* and *l_num* in message passing to the Spearman correlation on six SpaGCN embedding samples. The horizontal axis represents the number of layers in the message passing. The vertical axis represents the Spearman correlation measured by the ground-truth ranking and the ranking of the MP-MIM. *k* denotes the number of nearest neighbors, which is the predefined parameter in the KNN graph.

1.11 Supplemental Figure S11

1.11.1 151507

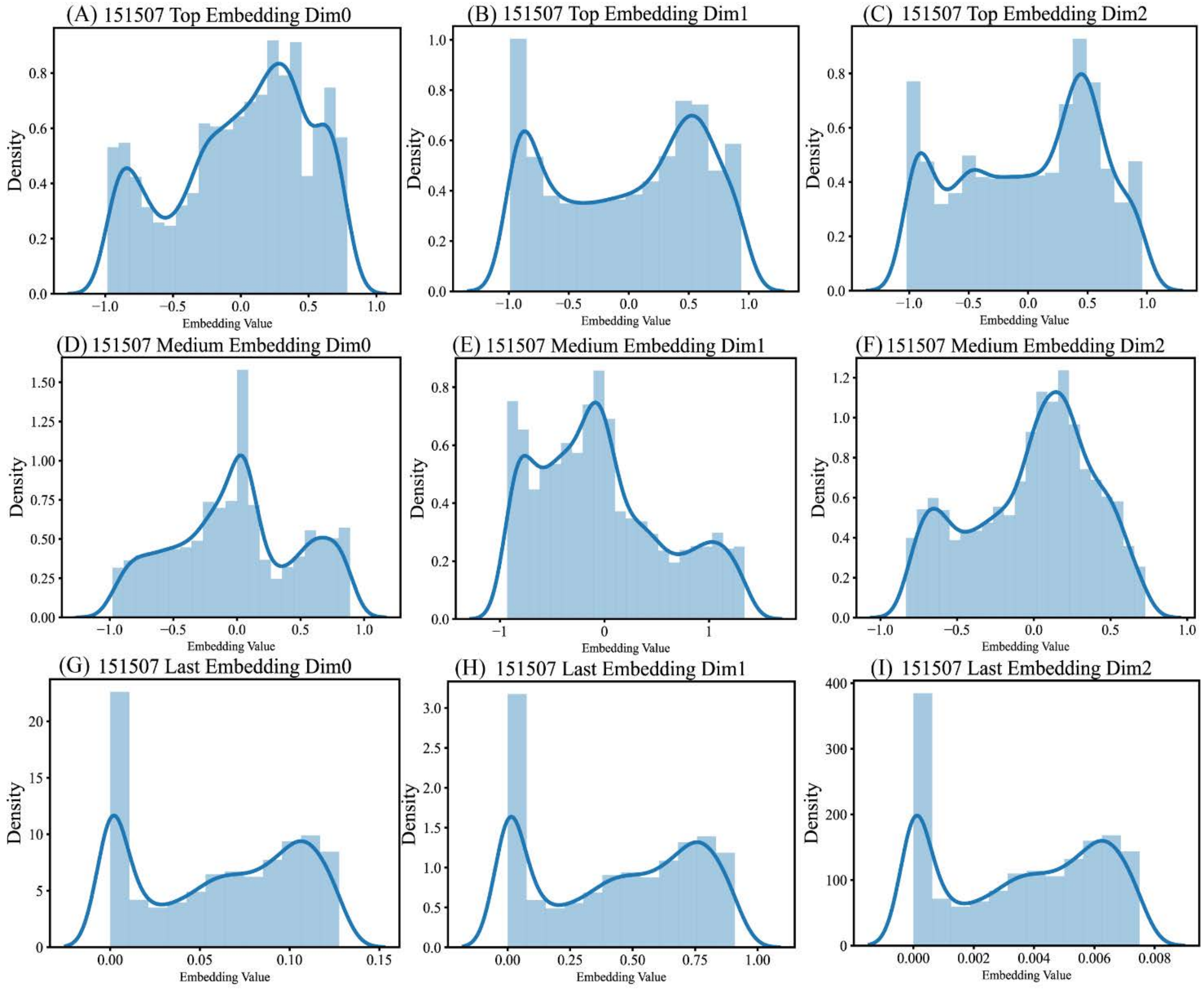

1.11.2 151510

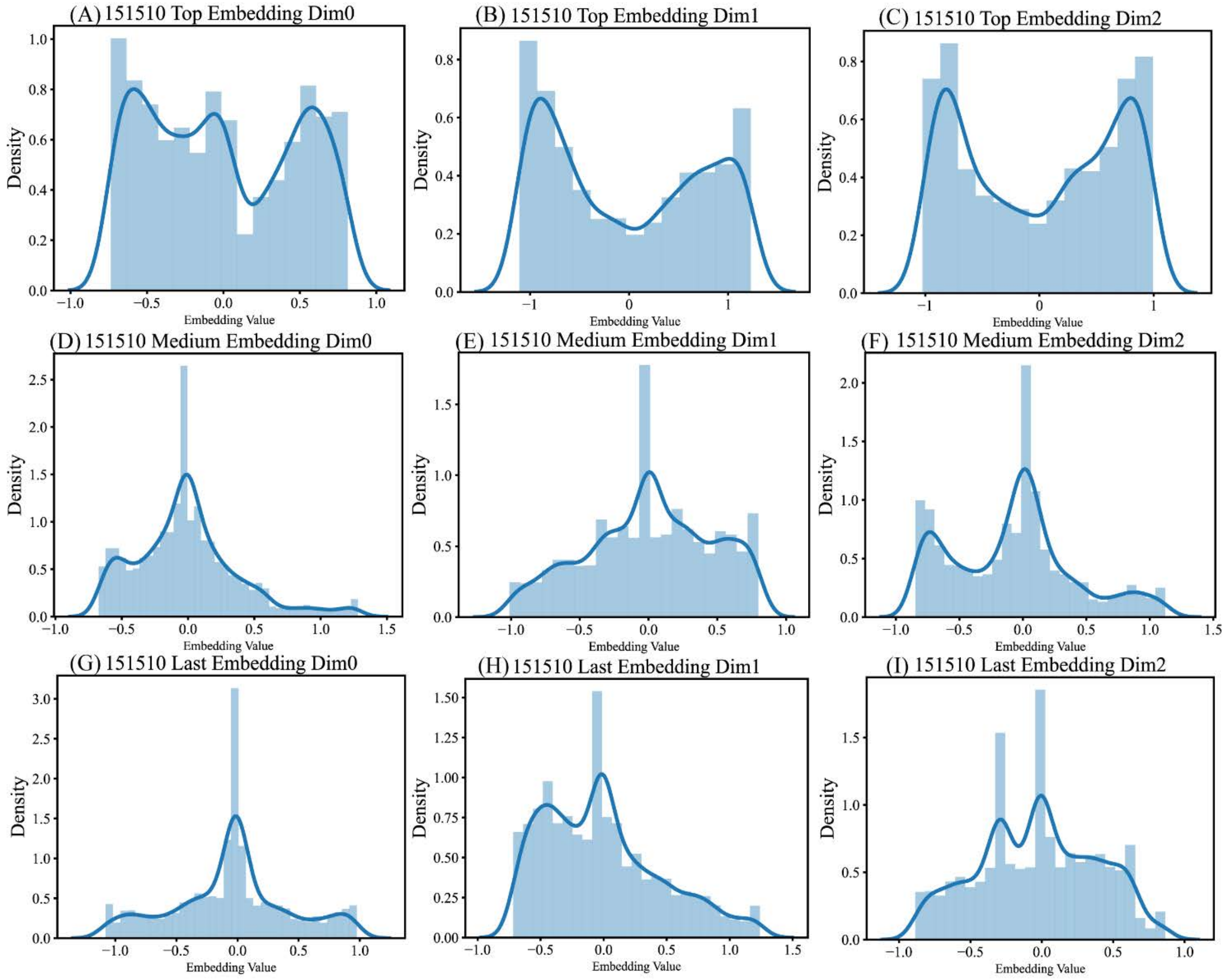
(A) 151510 Top Embedding Dim0
(B) 151510 Top Embedding Dim1
(C) 151510 Top Embedding Dim2
(D) 151510 Medium Embedding Dim0
(E) 151510 Medium Embedding Dim1
(F) 151510 Medium Embedding Dim2
(G) 151510 Last Embedding Dim0
(H) 151510 Last Embedding Dim1
(I) 151510 Last Embedding Dim2
Density
Embedding Value

1.11.3 151673

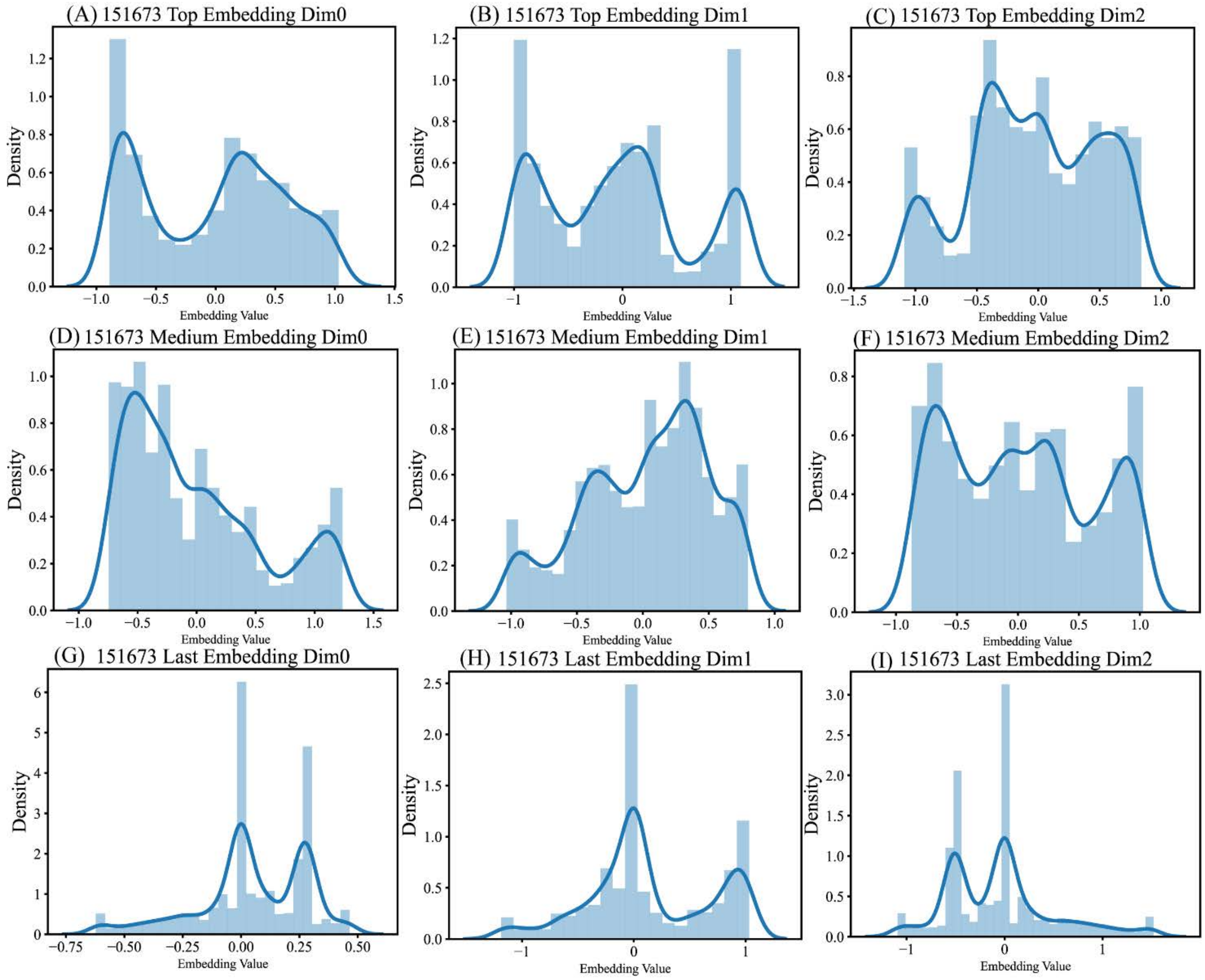
(A) 151673 Top Embedding Dim0
(B) 151673 Top Embedding Dim1
(C) 151673 Top Embedding Dim2
(D) 151673 Medium Embedding Dim0
(E) 151673 Medium Embedding Dim1
(F) 151673 Medium Embedding Dim2
(G) 151673 Last Embedding Dim0
(H) 151673 Last Embedding Dim1
(I) 151673 Last Embedding Dim2
Density
Embedding Value

1.11.4 151669

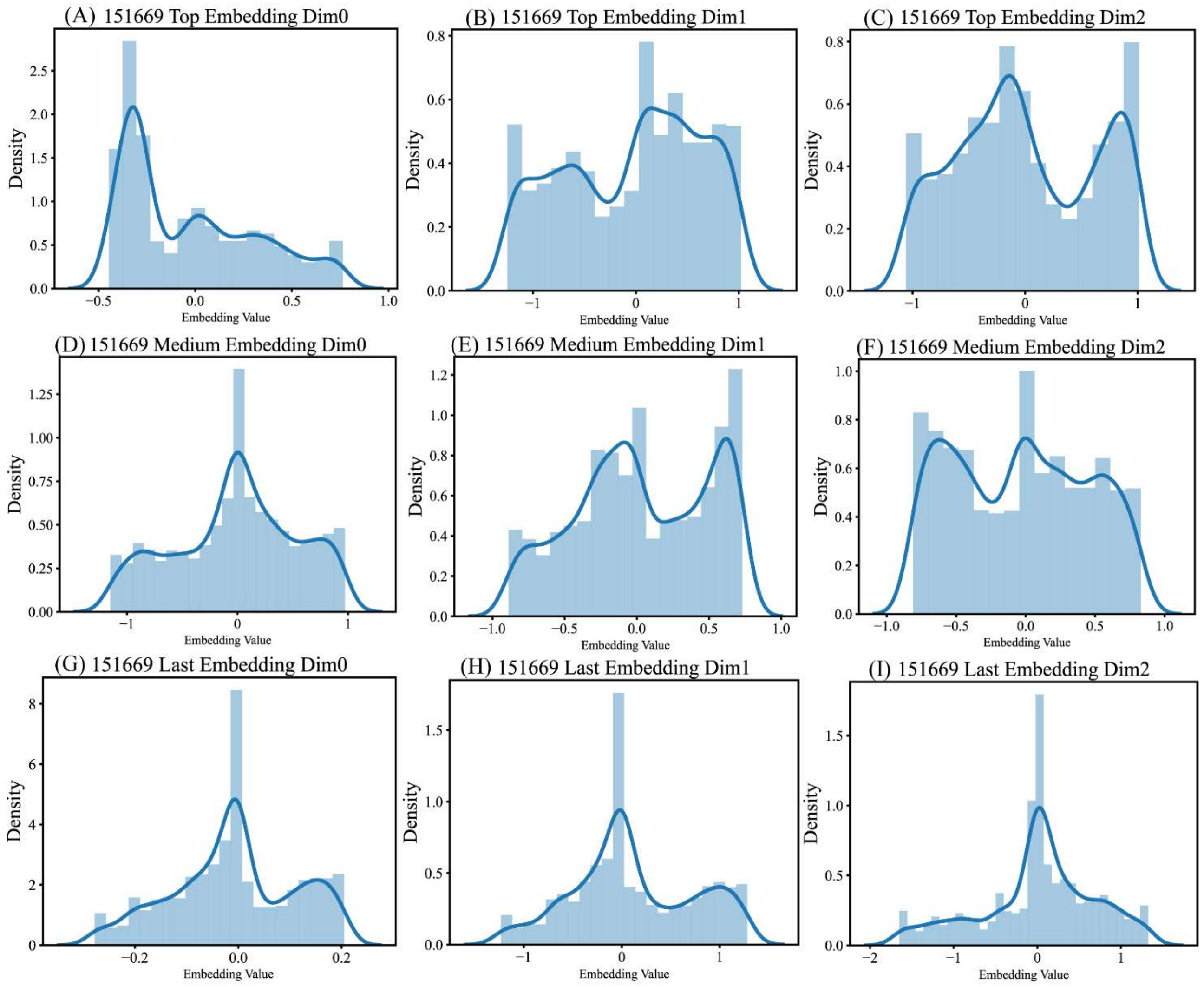

(A) 151669 Top Embedding Dim0
(B) 151669 Top Embedding Dim1
(C) 151669 Top Embedding Dim2
(D) 151669 Medium Embedding Dim0
(E) 151669 Medium Embedding Dim1
(F) 151669 Medium Embedding Dim2
(G) 151669 Last Embedding Dim0
(H) 151669 Last Embedding Dim1
(I) 151669 Last Embedding Dim2
Density
Embedding Value

1.11.5 151670

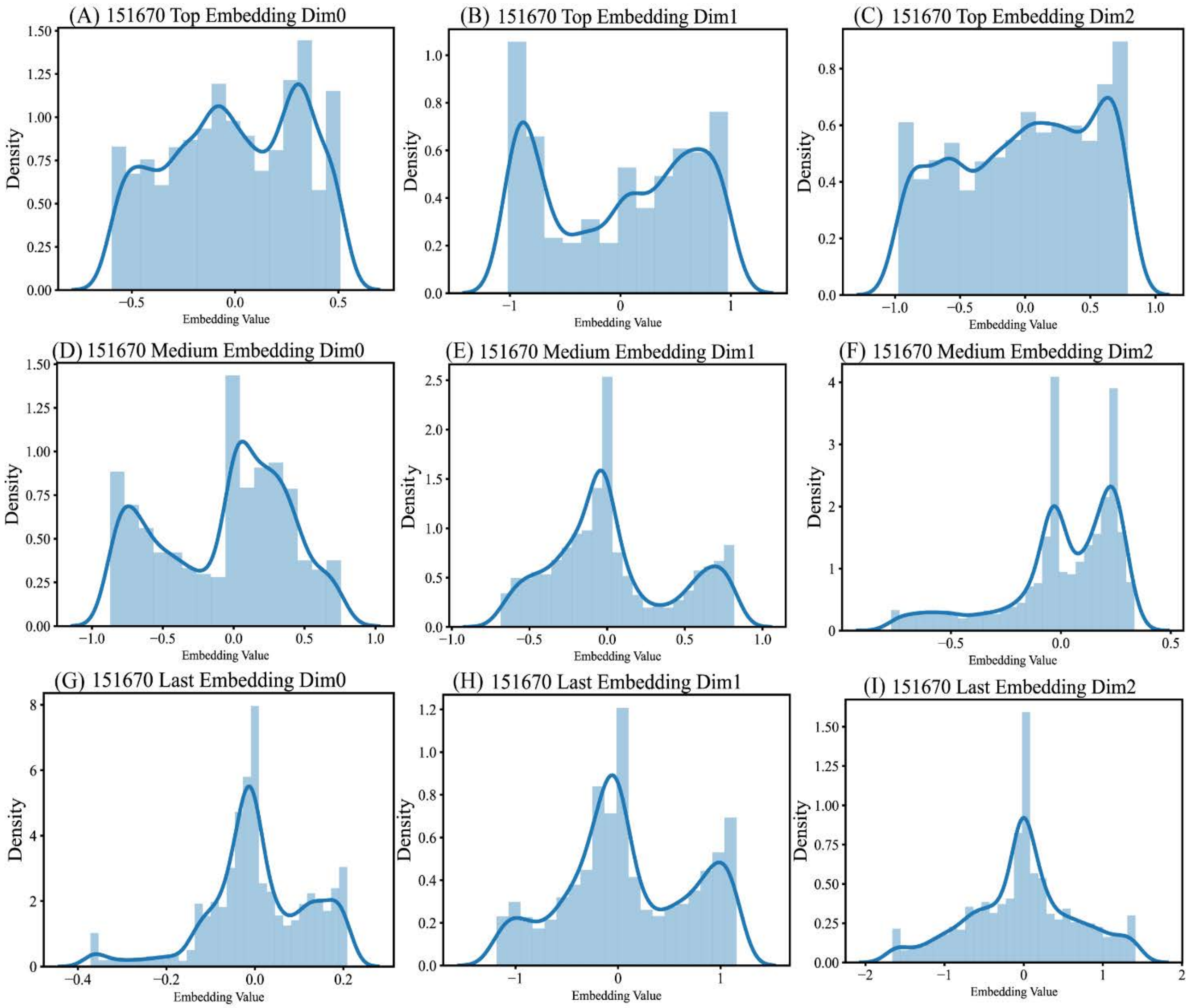

(A) 151670 Top Embedding Dim0
(B) 151670 Top Embedding Dim1
(C) 151670 Top Embedding Dim2
(D) 151670 Medium Embedding Dim0
(E) 151670 Medium Embedding Dim1
(F) 151670 Medium Embedding Dim2
(G) 151670 Last Embedding Dim0
(H) 151670 Last Embedding Dim1
(I) 151670 Last Embedding Dim2
Density
Embedding Value

1.11.6 2-8

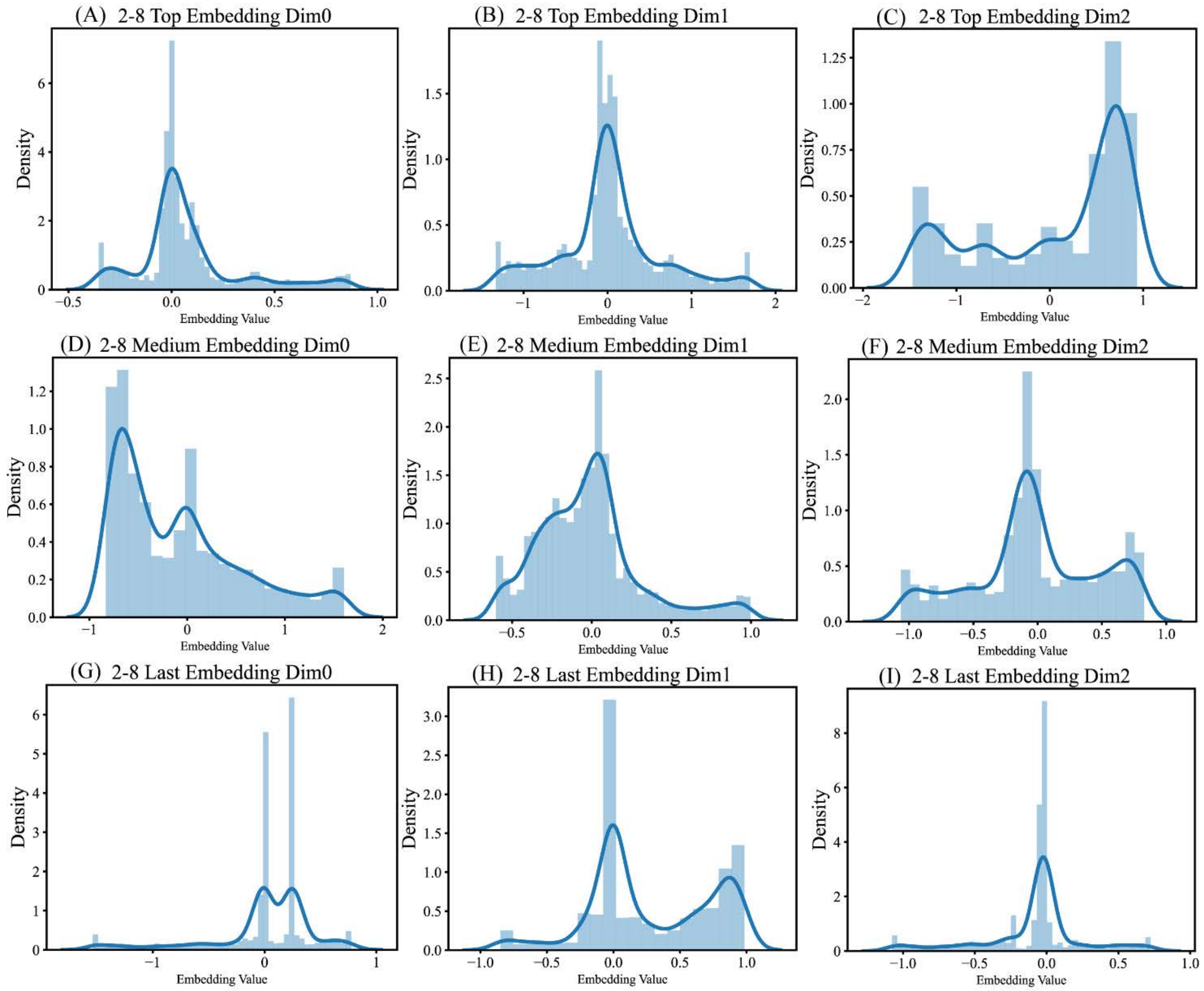

Supplemental Figure S11. Comparison results of different distributions of graph embeddings ranked as top, medium, and bottom by MP-MIM rankings on six RESEPT embedding samples. 151507, 151510, and 151673 samples have promising results, and 151669, 151670, and 2-8 samples have poor results in Spearman correlation analysis. The distributions of first, medium, and bottom-ranked graph embeddings are shown in the first, second, and third rows, respectively. In each subfigure of a particular dimension, the horizontal axis shows all the values in each dimension, and the vertical axis shows the density of different values.

1.12 Supplemental Figure S12

1.12.1 151507

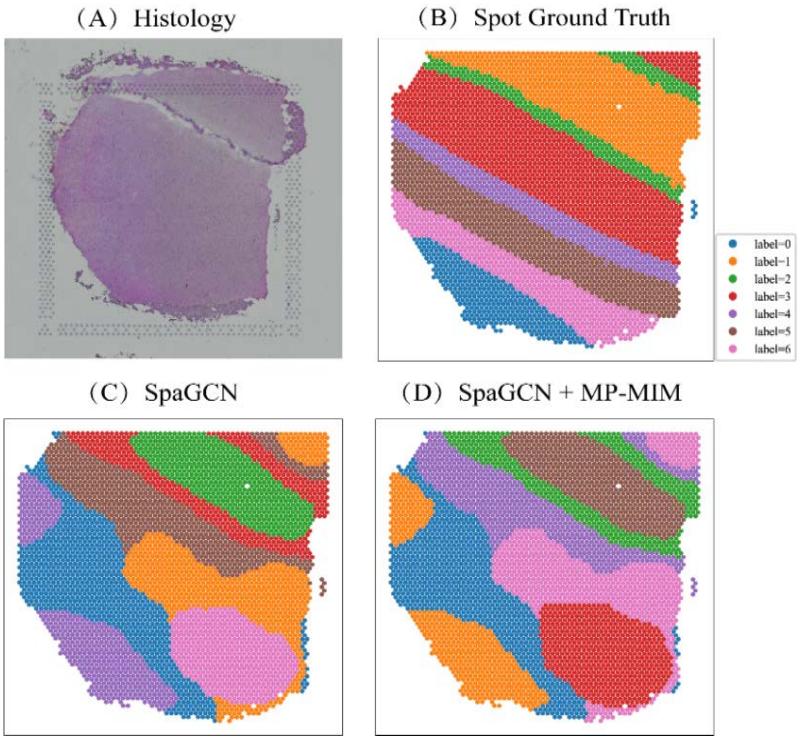

1.12.2 151508

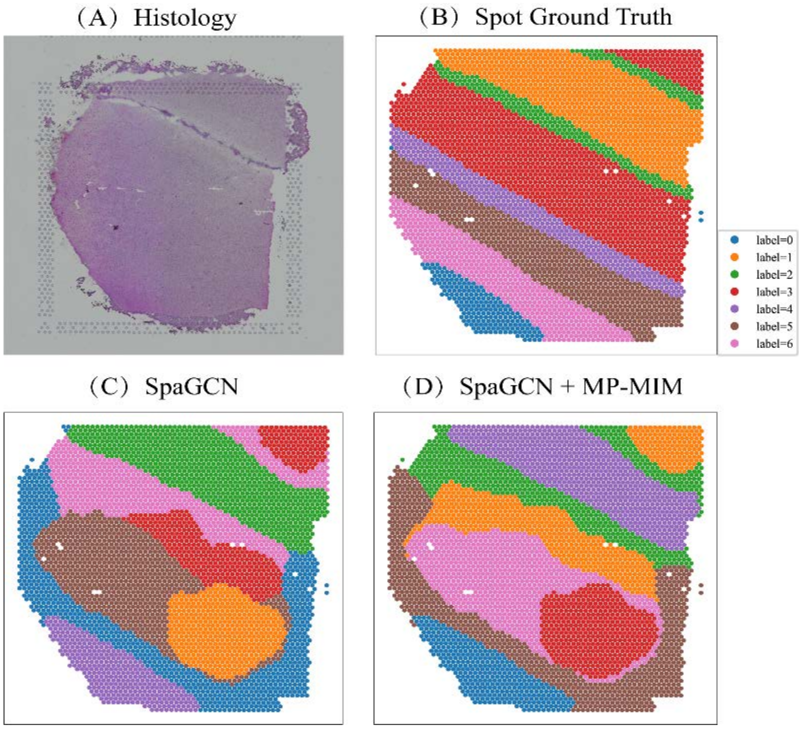

1.12.3 151509

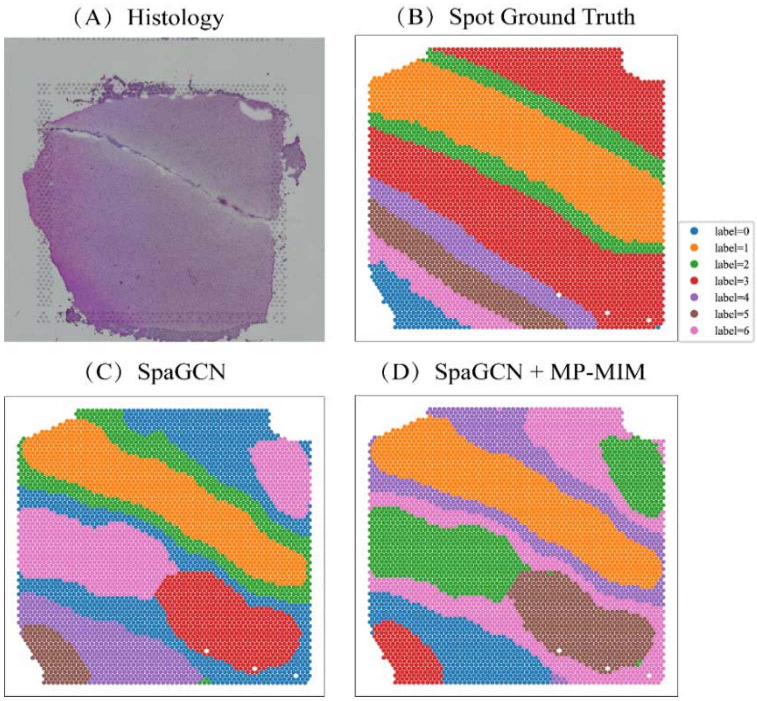

1.12.4 151510

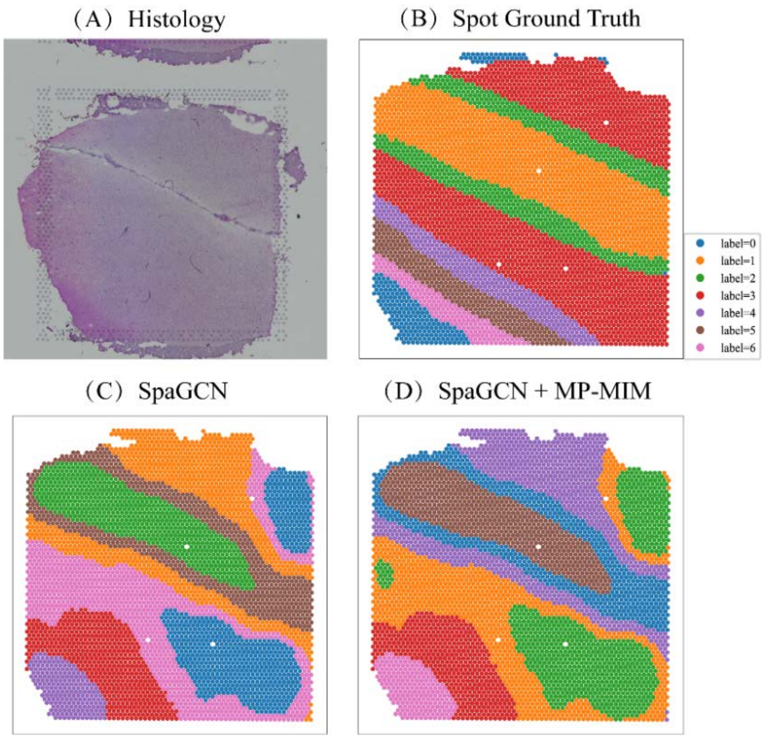

1.12.5 151669

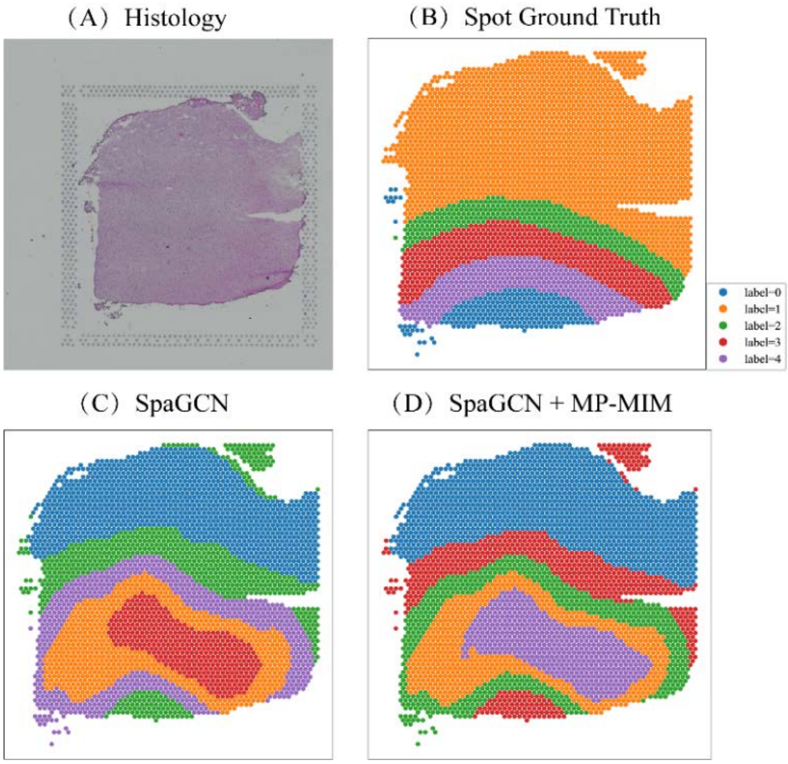

1.12.6 151670

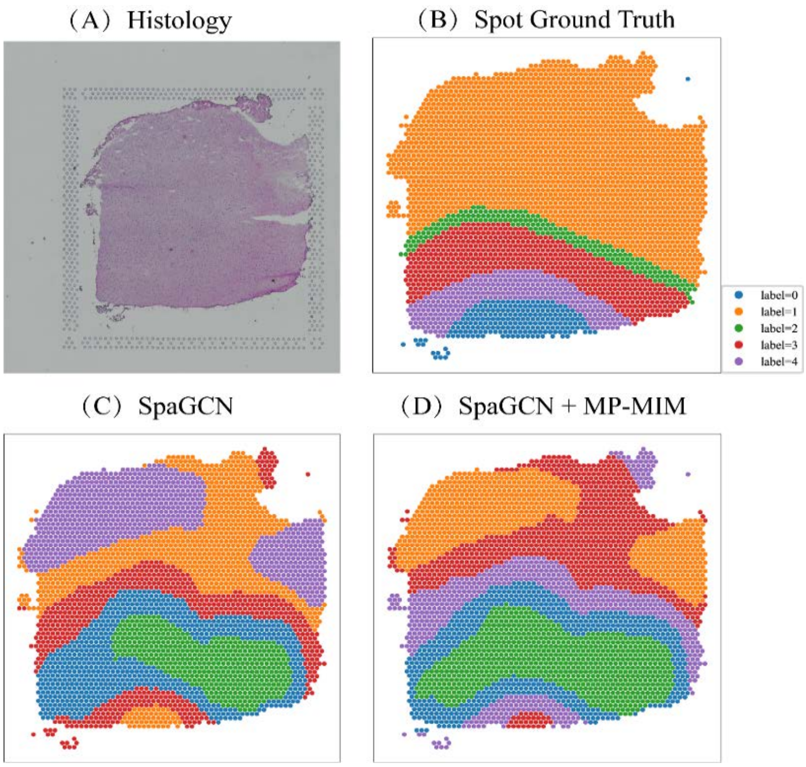

1.12.7 151671

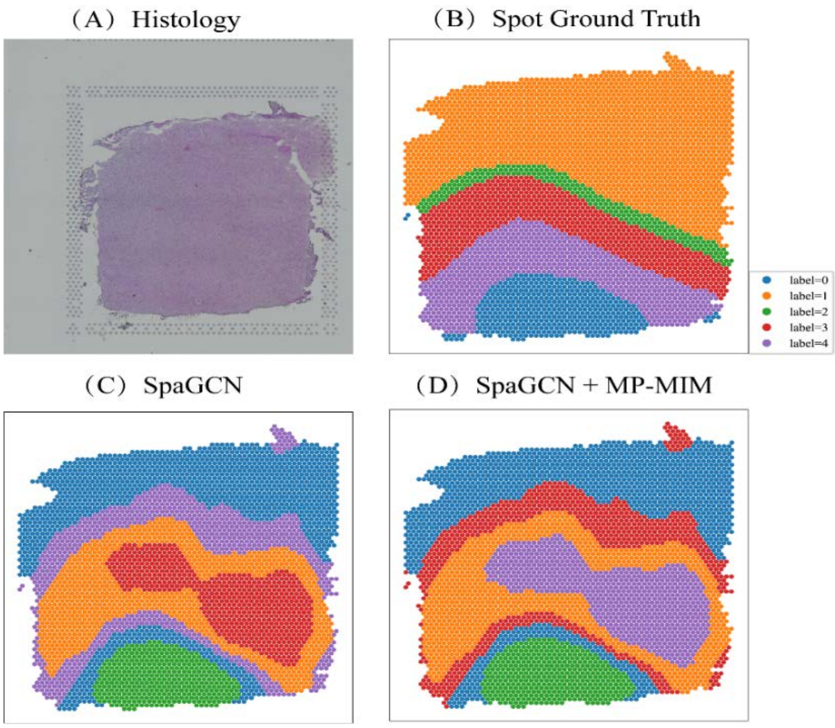

1.12.8 151672

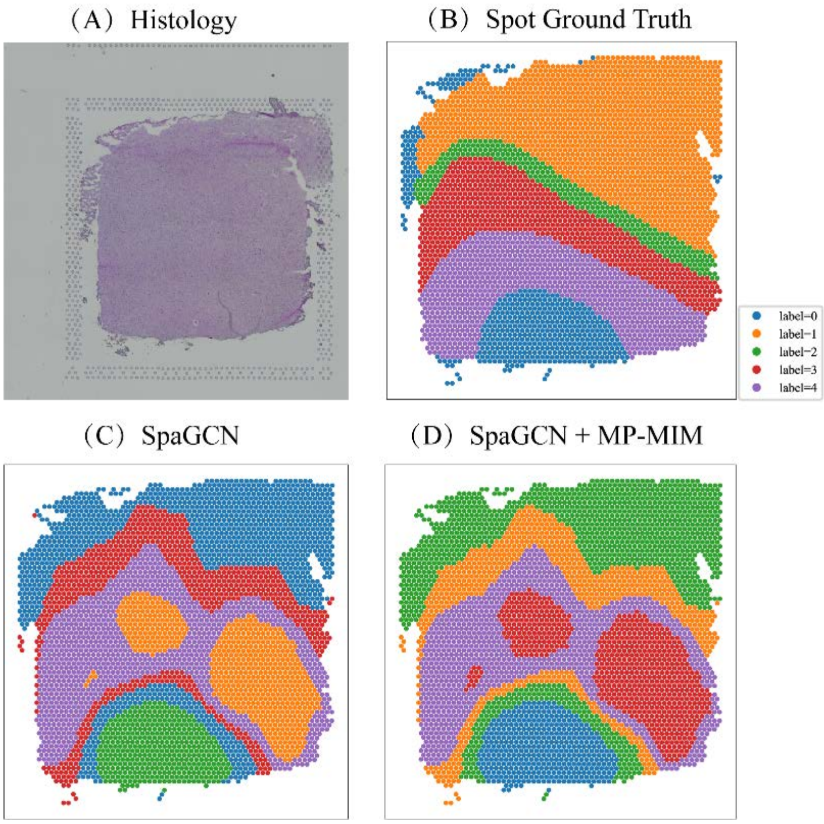

1.12.9 151673

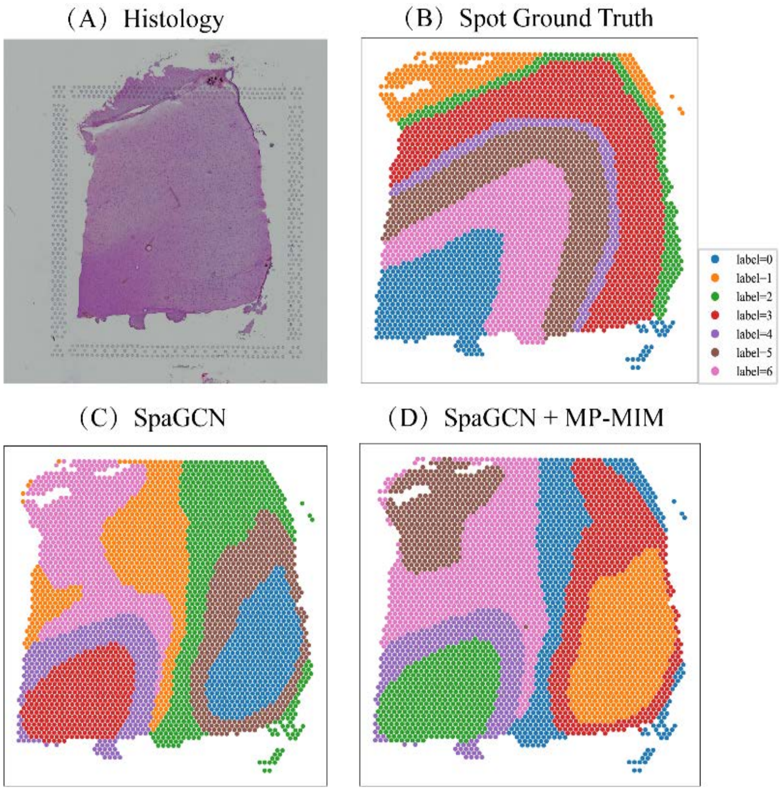

1.12.10 151674

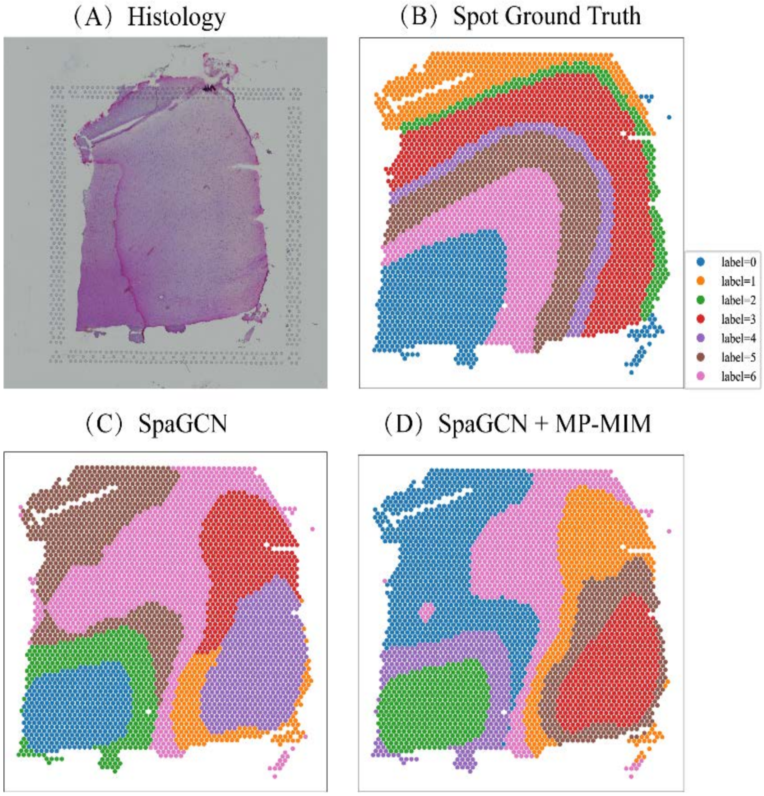

1.12.11 151675

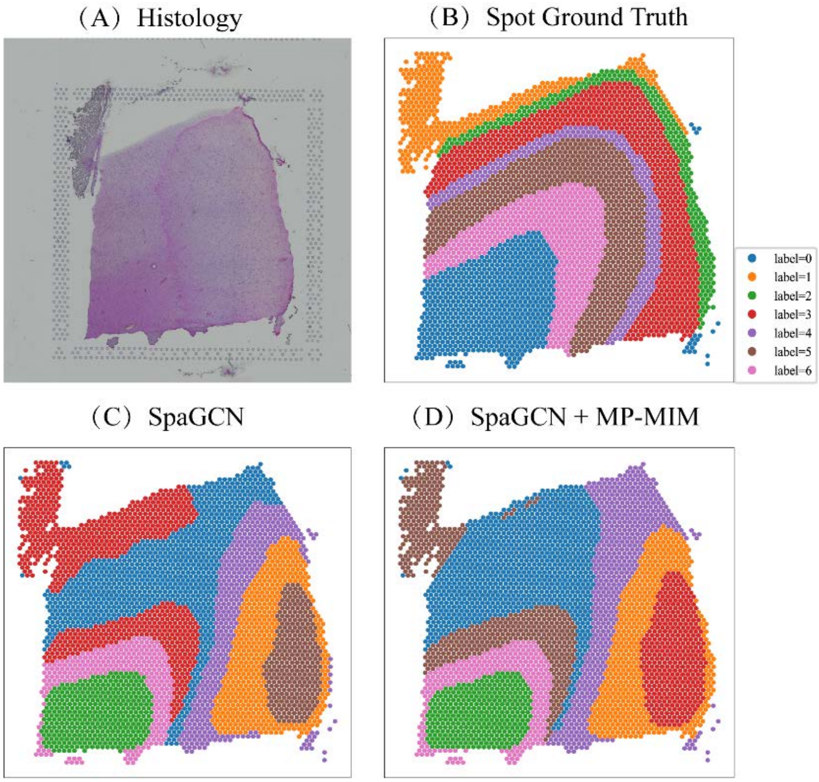

1.12.12 151676

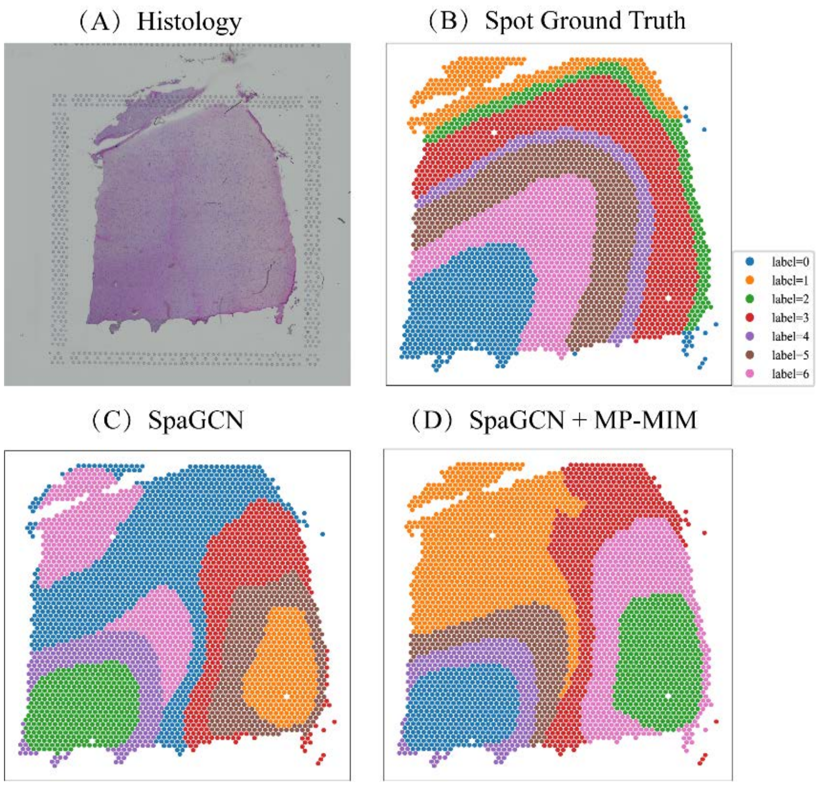

1.12.13 2-5

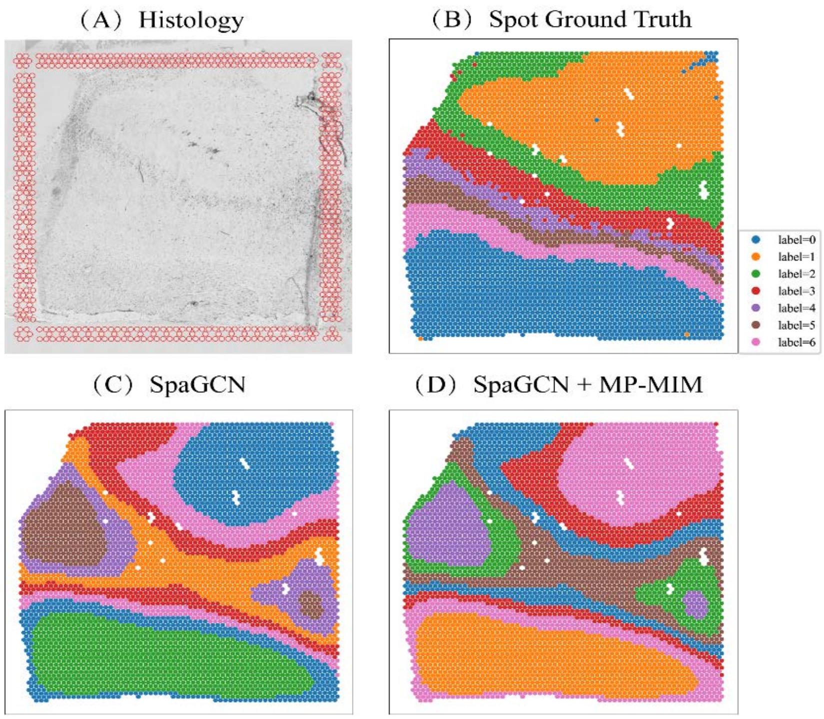

1.12.14 2-8

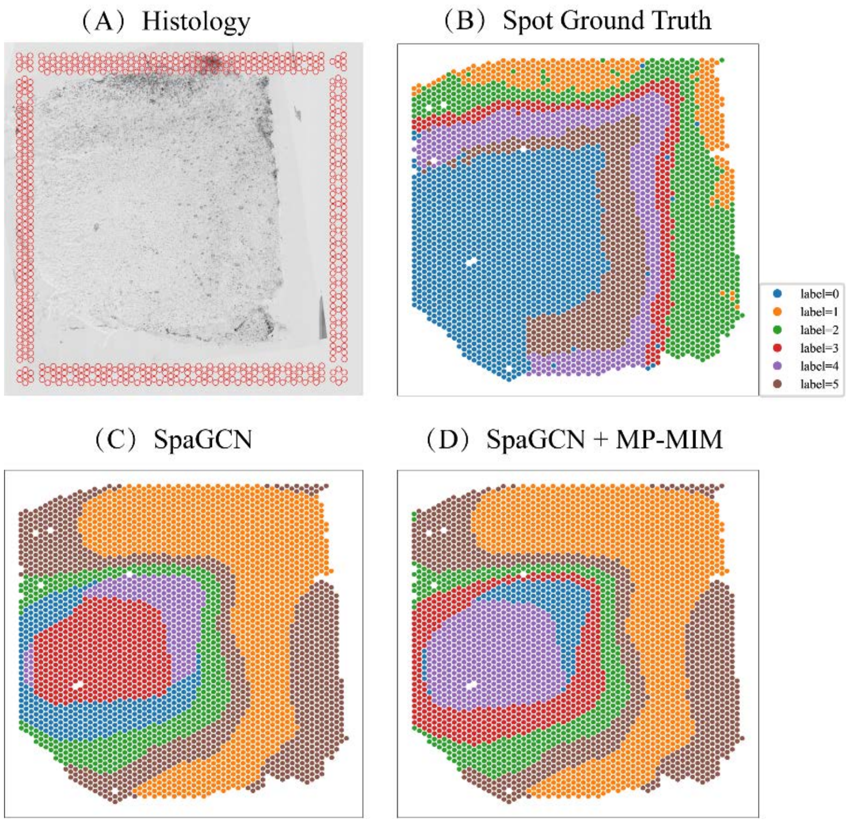

1.12.15 18-64

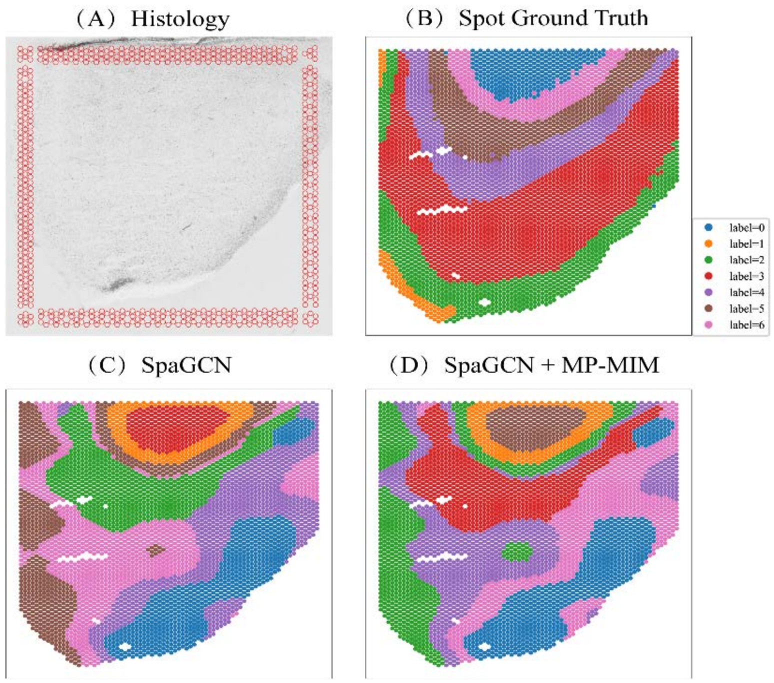

1.12.16 T4857

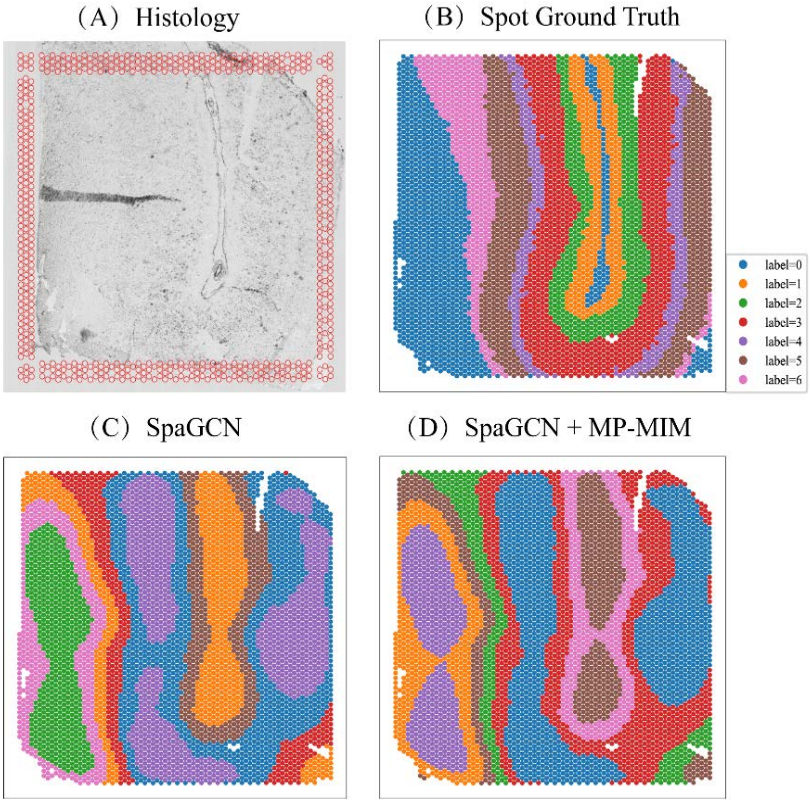

Supplemental Figure S12. Comparison of tissue architectures among the ground truth, original SpaGCN and SpaGCN after integrated with MP-MIM on 16 samples. **(A)** shows the actual histology. **(B)** shows the ground truth label in spot resolution. **(C)** and **(D)** are the tissue architecture identifications of original SpaGCN and SpaGCN integrated with MP-MIM, respectively.

1.13 Supplemental Figure S13

1.13.1 151507

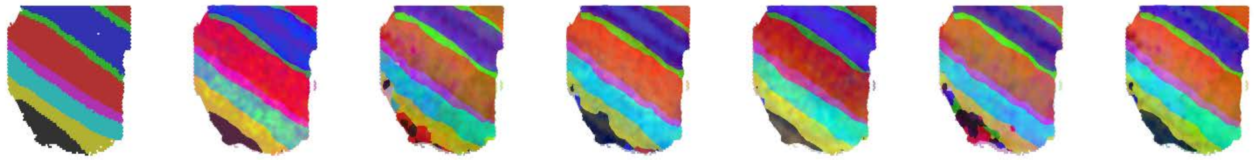

(A) Ground Truth. (B) Baseline Segmentation. (C) First in Top-5. (D) Second in Top-5. (E) Third in Top-5. (F) Fourth in Top-5. (G) Fifth in Top-5.

1.13.2 151508

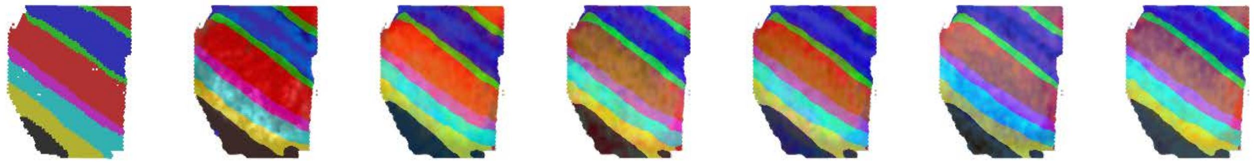

(A) Ground Truth. (B) Baseline Segmentation. (C) First in Top-5. (D) Second in Top-5. (E) Third in Top-5. (F) Fourth in Top-5. (G) Fifth in Top-5.

1.13.3 151509

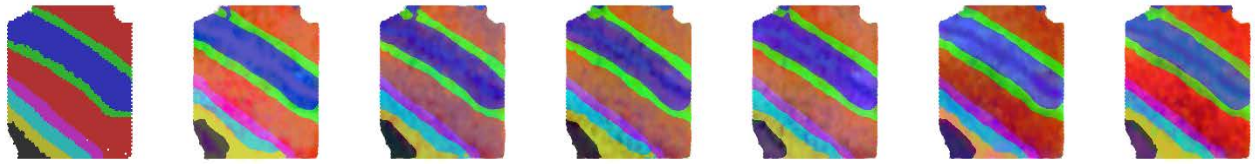

(A) Ground Truth. (B) Baseline Segmentation. (C) First in Top-5. (D) Second in Top-5. (E) Third in Top-5. (F) Fourth in Top-5. (G) Fifth in Top-5.

1.13.4 151510

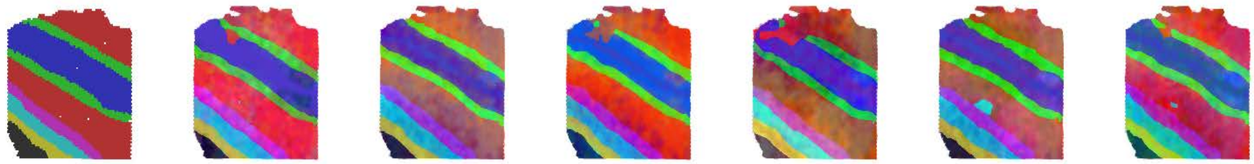

(A) Ground Truth. (B) Baseline Segmentation. (C) First in Top-5. (D) Second in Top-5. (E) Third in Top-5. (F) Fourth in Top-5. (G) Fifth in Top-5.

1.13.5 151669

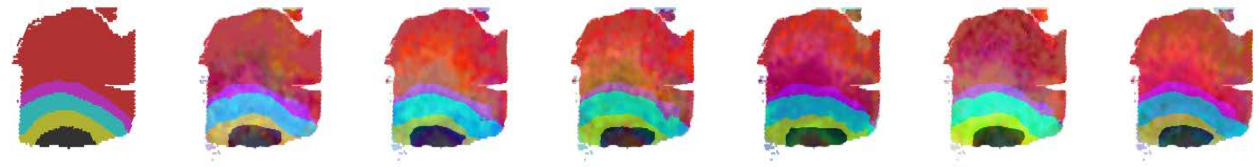

(A) Ground Truth. (B) Baseline Segmentation. (C) First in Top-5. (D) Second in Top-5. (E) Third in Top-5. (F) Fourth in Top-5. (G) Fifth in Top-5.

1.13.6 151670

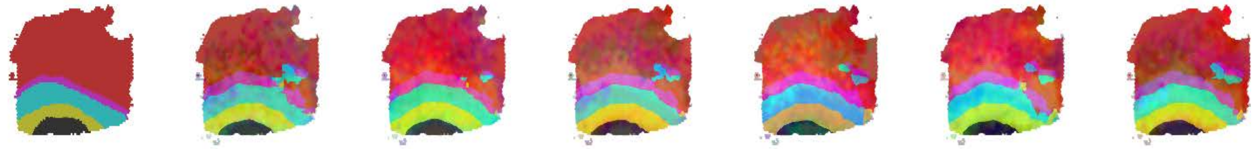

(A) Ground Truth. (B) Baseline Segmentation. (C) First in Top-5. (D) Second in Top-5. (E) Third in Top-5. (F) Fourth in Top-5. (G) Fifth in Top-5.

1.13.7 151671

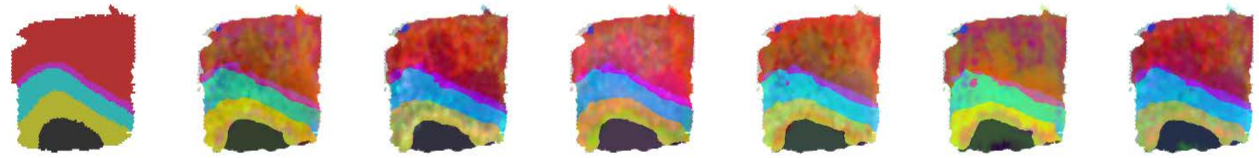

(A) Ground Truth. (B) Baseline Segmentation. (C) First in Top-5. (D) Second in Top-5. (E) Third in Top-5. (F) Fourth in Top-5. (G) Fifth in Top-5.

1.13.8 151672

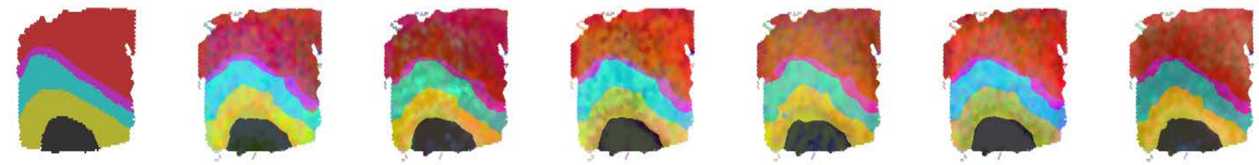

(A) Ground Truth. (B) Baseline Segmentation. (C) First in Top-5. (D) Second in Top-5. (E) Third in Top-5. (F) Fourth in Top-5. (G) Fifth in Top-5.

1.13.9 151673

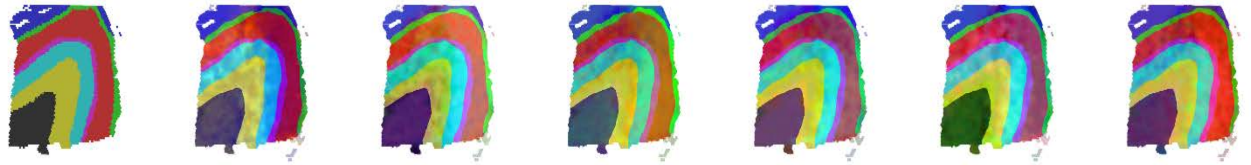

(A) Ground Truth. (B) Baseline Segmentation. (C) First in Top-5. (D) Second in Top-5. (E) Third in Top-5. (F) Fourth in Top-5. (G) Fifth in Top-5.

1.13.10 151674

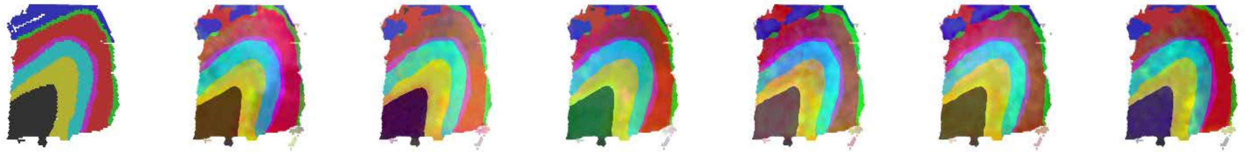

(A) Ground Truth. (B) Baseline Segmentation. (C) First in Top-5. (D) Second in Top-5. (E) Third in Top-5. (F) Fourth in Top-5. (G) Fifth in Top-5.

1.13.11 151675

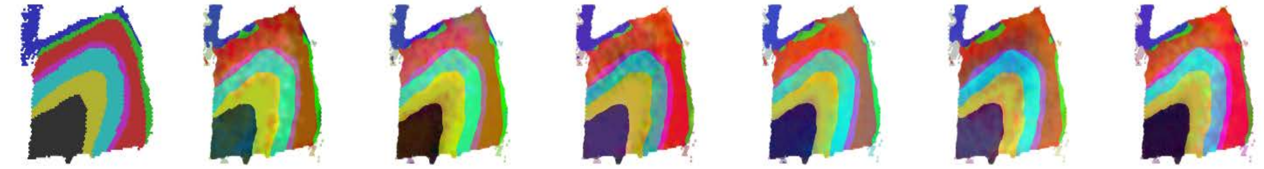

(A) Ground Truth. (B) Baseline Segmentation. (C) First in Top-5. (D) Second in Top-5. (E) Third in Top-5. (F) Fourth in Top-5. (G) Fifth in Top-5.

1.13.12 151676

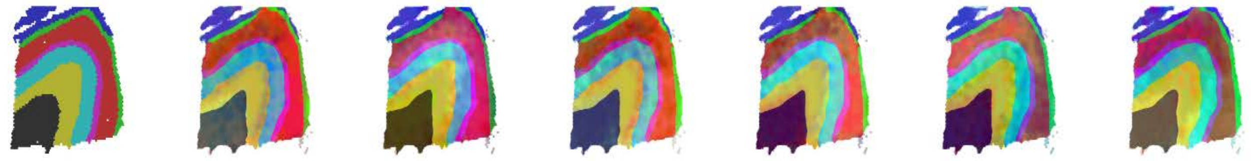

(A) Ground Truth. (B) Baseline Segmentation. (C) First in Top-5. (D) Second in Top-5. (E) Third in Top-5. (F) Fourth in Top-5. (G) Fifth in Top-5.

1.13.13 2-5

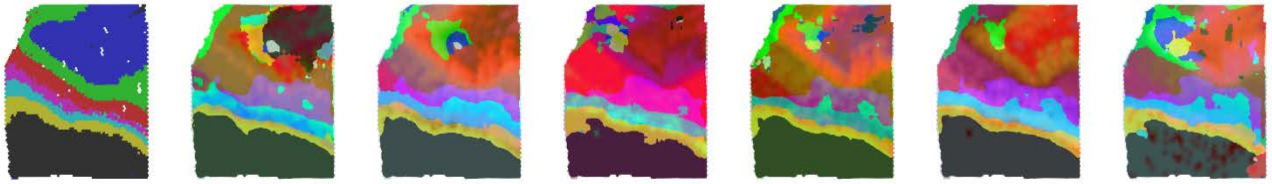

(A) Ground Truth. (B) Baseline Segmentation. (C) First in Top-5. (D) Second in Top-5. (E) Third in Top-5. (F) Fourth in Top-5. (G) Fifth in Top-5.

1.13.14 2-8

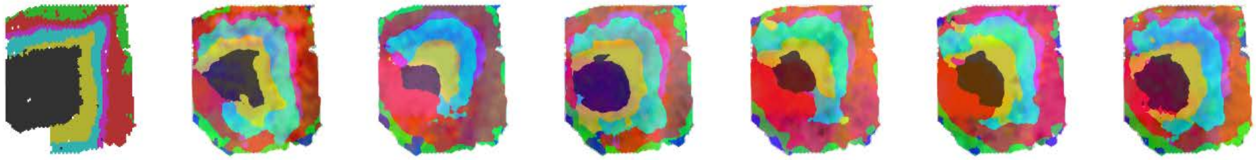

(A) Ground Truth. (B) Baseline Segmentation. (C) First in Top-5. (D) Second in Top-5. (E) Third in Top-5. (F) Fourth in Top-5. (G) Fifth in Top-5.

1.13.15 18-64

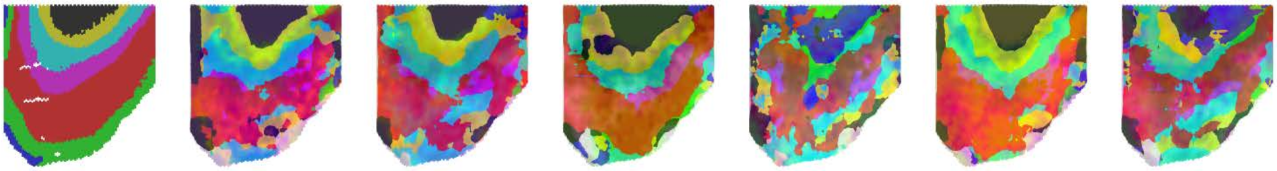

(A) Ground Truth. (B) Baseline Segmentation. (C) First in Top-5. (D) Second in Top-5. (E) Third in Top-5. (F) Fourth in Top-5. (G) Fifth in Top-5.

1.13.16 T4857

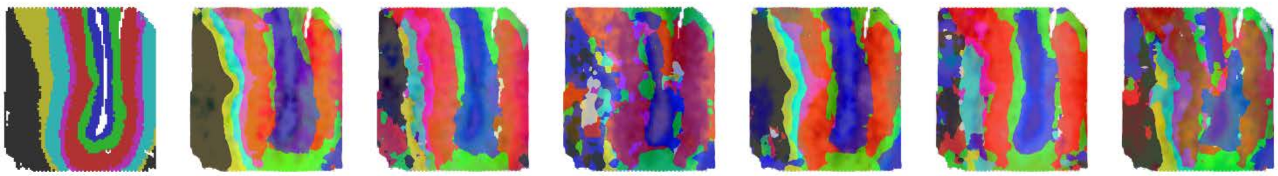

(A) Ground Truth. (B) Baseline Segmentation. (C) First in Top-5. (D) Second in Top-5. (E) Third in Top-5. (F) Fourth in Top-5. (G) Fifth in Top-5.

Supplemental Figure S13. Comparison segmentations among the ground truth, baseline of RESEPT, and Top 5 of RESEPT after integrated MP-MIM on 16 samples. **(A)** shows the segmentation ground truth in the pixel resolution of RGB image. **(B)** shows the baseline segmentation with the median value of all ARIs from RESEPT. From **(C)** to **(G)**, they are the top 5 segmentations of MP-MIM in RESEPT.

2 Supplemental Tables

2.1 Supplemental Table S1

Message Passing	*Moran's I*	*Parameter*	*Considered Value*	*Integration Model*
Basic graph convolution	KNN weight matrix	***k_num***	10,20,30,…,70,80,90	SpaGCN
		l_num	1,2,3,…,13,14,15	
		$k_{nearest}$	4	
Distance-based graph convolution	Radius distance weight matrix	***k_num***	10,20,30,…,70,80,90	RESEPT
		l_num	1,2,3,…,13,14,15	
		d_{radius}	$\sqrt{2}$	

Supplemental Table S1. Hyperparameter settings in MP-MIM, where *k_num* is the preset parameter of the KNN graph and *l_num* is the number of layers for the message passing, $k_{nearest}$ is the number of nearest neighbors, and d_{radius} is the distance threshold in the spatial weight matrix of Moran's I.

2.2 Supplemental Table S2

Parameter	*Considered Value*
Zdim	3,10,16,32,64,128,256
PEalpha	0.1,0.2,0.3,0.5,1.0,1.2,1.5,2.0

Supplemental Table S2. The details about the parameter settings in RESEPT, where *Zdim* is the number of latent space dimensions, and *PEalpha* is the degree of employing location information in feature autoencoder.

2.3 Supplemental Table S3

Parameter	*Considered Value*
PCA_num	5,50
Resolution	0.1,1,3,5,7,10
L_value	0.43,1.43,2.43

Supplemental Table S3. The details about the hyperparameter settings in SpaGCN, where *PCA_num* is the number of dimensions of GCN input, *Resolution* is the setting of initial Louvain's clustering, and *L_value* controls the percentage of total expression contributed by neighborhoods.

2.4 Supplemental Table S4

Sample	***151507***	***151508***	***151509***	***151510***	***151669***	***151670***	***151671***	***151672***
Label_num	7	7	7	7	5	5	5	5
Spot_num	4226	4384	4789	4634	3661	3498	4110	4015
Sample	***151673***	***151674***	***151675***	***151676***	***2-5***	***2-8***	***18-64***	***T4857***
Label_num	7	7	7	7	7	6	7	7
Spot_num	3639	3673	3592	3460	4701	3445	4225	4832

Supplemental Table S4. The details about the experimental dataset, where *Label_num* is the number of ground-truth labels, and *Spot_num* is the number of spots in each sample.

2.5 Supplemental Table S5

2.5.1 151507

**MI_emb0_top5**	**MI_emb1_top5**	***MI_emb2_top5***	**MP_MIM_top5**
pe0.5_zdim256 (**1**)	pe0.5_zdim128 (9)	pe2.0_zdim256 (12)	pe2.0_zdim256 (12)
pe0.2_zdim256 (4)	pe0.2_zdim256 (4)	pe1.5_zdim256 (11)	pe0.5_zdim256 (**1**)
pe2.0_zdim64 (20)	pe1.0_zdim256 (8)	pe0.5_zdim256 (**1**)	pe0.5_zdim128 (9)
pe0.3_zdim32 (7)	pe1.2_zdim256 (19)	pe0.1_zdim256 (2)	pe0.2_zdim256 (4)
pe1.2_zdim64 (15)	pe1.5_zdim128 (6)	pe1.0_zdim128 (3)	pe1.5_zdim256 (11)

2.5.2 151508

MI_emb0_top5	**MI_emb1_top5**	**MI_emb2_top5**	**MP_MIM_top5**
pe2.0_zdim256 (**1**)	pe2.0_zdim256 (**1**)	pe2.0_zdim256 (**1**)	pe2.0_zdim256 (**1**)
pe0.5_zdim256 (2)	pe0.5_zdim128 (29)	pe0.5_zdim128 (29)	pe0.5_zdim128 (29)
pe1.2_zdim256 (6)	pe1.2_zdim256 (6)	pe1.0_zdim256 (27)	pe1.2_zdim256 (6)
pe2.0_zdim128 (4)	pe0.2_zdim256 (3)	pe0.2_zdim256 (3)	pe0.5_zdim256 (2)
pe1.5_zdim128 (24)	pe0.5_zdim256 (2)	pe1.0_zdim128 (5)	pe0.2_zdim256 (3)

2.5.3 151509

**MI_emb0_top5**	***MI_emb1_top5***	**MI_emb2_top5**	***MP_MIM_top5***
pe1.0_zdim256 (5)	pe2.0_zdim256 (7)	pe1.0_zdim256 (5)	pe1.0_zdim256 (5)
pe1.2_zdim256 (4)	pe1.0_zdim256 (5)	pe2.0_zdim256 (7)	pe1.2_zdim256 (4)
pe0.3_zdim256 (2)	pe1.2_zdim256 (4)	pe1.2_zdim128 (6)	pe2.0_zdim256 (7)
pe0.2_zdim256 (**1**)	pe0.2_zdim256 (**1**)	pe2.0_zdim128 (10)	pe0.3_zdim256 (2)
pe1.5_zdim256 (8)	pe0.3_zdim256 (2)	pe1.2_zdim256 (4)	pe0.2_zdim256 (**1**)

2.5.4 151510

MI_emb0_top5	*MI_emb1_top5*	MI_emb2_top5	*MP_MIM_top5*
pe2.0_zdim256 (11)	pe0.2_zdim256 (9)	pe0.5_zdim256 (6)	pe0.5_zdim256 (6)
pe0.5_zdim256 (6)	pe0.5_zdim128 (5)	pe1.0_zdim128 (16)	pe0.2_zdim256 (9)
pe1.0_zdim128 (10)	pe2.0_zdim256 (11)	pe0.2_zdim256 (9)	pe0.5_zdim128 (5)
pe0.2_zdim256 (9)	pe0.5_zdim256 (6)	pe0.1_zdim128 (3)	pe2.0_zdim256 (11)
pe1.5_zdim256 (18)	pe1.2_zdim256 (**1**)	pe1.5_zdim256 (18)	pe1.2_zdim256 (**1**)

2.5.5 151669

MI_emb0_top5	MI_emb1_top5	MI_emb2_top5	MP_MIM_top5
pe1.2_zdim128 (9)	pe1.0_zdim64 (35)	pe1.2_zdim256 (**6**)	pe1.2_zdim256 (**6**)
pe0.5_zdim128 (7)	pe1.5_zdim128 (19)	pe0.2_zdim128 (21)	pe0.2_zdim128 (21)
pe2.0_zdim256 (11)	pe1.5_zdim256 (36)	pe2.0_zdim128 (32)	pe2.0_zdim128 (32)
pe1.2_zdim256 (**6**)	pe0.5_zdim256 (12)	pe1.0_zdim256 (39)	pe1.0_zdim64 (35)
pe0.3_zdim256 (27)	pe0.3_zdim128 (56)	pe0.5_zdim128 (7)	pe1.5_zdim128 (19)

2.5.6 151670

MI_emb0_top5	*MI_emb1_top5*	MI_emb2_top5	MP_MIM_top5
pe2.0_zdim128 (42)	pe2.0_zdim256 (8)	pe1.5_zdim256 (39)	pe2.0_zdim256 (8)
pe1.0_zdim64 (5)	pe1.0_zdim256 (**2**)	pe1.0_zdim256 (**2**)	pe1.5_zdim256 (39)
pe1.0_zdim128 (18)	pe0.5_zdim256 (9)	pe0.5_zdim64 (27)	pe2.0_zdim128 (42)
pe2.0_zdim256 (8)	pe1.2_zdim256 (7)	pe1.0_zdim32 (32)	pe1.0_zdim256 (**2**)
pe1.5_zdim64 (43)	pe1.5_zdim128 (28)	pe1.2_zdim3 (15)	pe0.5_zdim256 (9)

2.5.7 151671

MI_emb0_top5	MI_emb1_top5	*MI_emb2_top5*	MP_MIM_top5
pe1.5_zdim128 (**2**)	pe1.2_zdim64 (13)	pe0.5_zdim256 (7)	pe1.5_zdim128 (**2**)
pe1.0_zdim128 (10)	pe1.5_zdim64 (9)	pe2.0_zdim256 (5)	pe1.2_zdim64 (13)
pe0.5_zdim128 (23)	pe1.5_zdim256 (11)	pe1.5_zdim256 (11)	pe1.0_zdim128 (10)
pe1.2_zdim128 (6)	pe1.2_zdim256 (3)	pe1.2_zdim128 (6)	pe0.5_zdim256 (7)
pe1.0_zdim64 (15)	pe2.0_zdim256 (5)	pe0.1_zdim256 (4)	pe0.5_zdim128 (23)

2.5.8 151672

MI_emb0_top5	MI_emb1_top5	*MI_emb2_top5*	MP_MIM_top5
pe1.5_zdim128 (15)	pe1.5_zdim256 (**1**)	pe2.0_zdim256 (2)	pe2.0_zdim256 (2)
pe2.0_zdim64 (8)	pe1.0_zdim64 (13)	pe1.2_zdim256 (9)	pe1.5_zdim128 (15)
pe1.5_zdim256 (**1**)	pe1.2_zdim64 (4)	pe0.2_zdim256 (7)	pe1.2_zdim256 (9)
pe2.0_zdim128 (28)	pe1.5_zdim64 (10)	pe1.5_zdim256 (6)	pe2.0_zdim64 (8)
pe1.2_zdim128 (3)	pe0.5_zdim256 (5)	pe1.5_zdim256 (**1**)	pe1.5_zdim256 (**1**)

2.5.9 151673

MI_emb0_top5	MI_emb1_top5	MI_emb2_top5	*MP_MIM_top5*
pe1.5_zdim256 (12)	pe0.5_zdim256 (**2**)	pe1.2_zdim256 (11)	pe0.5_zdim256 (**2**)
pe1.5_zdim64 (16)	pe0.2_zdim256 (28)	pe2.0_zdim256 (4)	pe1.5_zdim256 (12)
pe1.5_zdim32 (17)	pe1.2_zdim128 (7)	pe1.0_zdim128 (6)	pe1.2_zdim256 (11)
pe0.3_zdim64 (20)	pe0.3_zdim256 (29)	pe1.0_zdim256 (13)	pe1.5_zdim64 (16)
pe0.1_zdim64 (10)	pe1.0_zdim32 (26)	pe1.0_zdim16 (40)	pe1.5_zdim32 (17)

2.5.10 151674

MI_emb0_top5	MI_emb1_top5	*MI_emb2_top5*	MP_MIM_top5
pe2.0_zdim32 (18)	pe0.3_zdim256 (12)	pe2.0_zdim128 (4)	pe0.3_zdim256 (12)
pe1.5_zdim32 (20)	pe0.2_zdim128 (35)	pe1.0_zdim256 (3)	pe0.2_zdim128 (35)
pe1.5_zdim256 (10)	pe1.5_zdim128 (26)	pe1.5_zdim16 (24)	pe2.0_zdim128 (4)
pe0.1_zdim64 (29)	pe2.0_zdim256 (5)	pe2.0_zdim16 (28)	pe1.0_zdim256 (3)
pe1.5_zdim64 (**1**)	pe1.0_zdim128 (37)	pe1.2_zdim256 (2)	pe2.0_zdim32 (18)

2.5.11 151675

MI_emb0_top5	MI_emb1_top5	MI_emb2_top5	MP_MIM_top5
pe0.1_zdim16 (24)	pe0.1_zdim256 (7)	pe0.3_zdim64 (8)	pe0.1_zdim256 (7)
pe2.0_zdim256 (12)	pe1.2_zdim256 (20)	pe0.3_zdim256 (17)	pe0.1_zdim16 (24)
pe1.2_zdim64 (10)	pe0.3_zdim256 (17)	pe0.1_zdim64 (25)	pe1.2_zdim256 (20)
pe0.5_zdim64 (16)	pe0.2_zdim256 (22)	pe1.0_zdim256 (14)	pe2.0_zdim256 (12)
pe1.0_zdim64 (9)	pe0.5_zdim256 (15)	pe1.5_zdim64 (**3**)	pe0.3_zdim64 (8)

2.5.12 151676

**MI_emb0_top5**	**MI_emb1_top5**	**MI_emb2_top5**	***MP_MIM_top5***
pe1.2_zdim64 (8)	pe0.2_zdim64 (18)	pe2.0_zdim128 (31)	pe1.2_zdim64 (8)
pe0.2_zdim32 (10)	pe0.3_zdim64 (9)	pe0.2_zdim256 (26)	pe0.2_zdim32 (10)
pe1.5_zdim16 (29)	e0.5_zdim256 (35)	pe2.0_zdim64 (7)	pe0.2_zdim64 (18)
pe1.5_zdim32 (24)	pe0.5_zdim64 (19)	pe1.2_zdim128 (3)	pe0.3_zdim64 (9)
pe0.5_zdim128 (23)	pe1.0_zdim128 (**2**)	pe0.1_zdim32 (20)	pe2.0_zdim128 (31)

2.5.13 2-5

MI_emb0_top5	**MI_emb1_top5**	**MI_emb2_top5**	**MP_MIM_top5**
pe0.2_zdim128 (5)	pe1.0_zdim32 (41)	pe0.3_zdim128 (6)	pe0.2_zdim128 (5)
pe0.2_zdim64 (9)	pe0.1_zdim64 (4)	pe0.5_zdim256 (16)	pe0.3_zdim128 (6)
pe0.5_zdim128 (11)	pe2.0_zdim256 (35)	pe0.2_zdim256 (10)	pe0.2_zdim64 (9)
pe0.3_zdim256 (**3**)	pe1.5_zdim64 (27)	pe1.0_zdim256 (14)	pe0.5_zdim128 (11)
pe0.1_zdim128 (7)	pe0.3_zdim256 (**3**)	pe0.5_zdim32 (19)	pe0.5_zdim256 (16)

2.5.14 2-8

**MI_emb0_top5**	***MI_emb1_top5***	**MI_emb2_top5**	**MP_MIM_top5**
pe0.5_zdim64 (11)	pe1.2_zdim256 (24)	pe0.2_zdim256 (15)	pe0.2_zdim256 (15)
pe0.3_zdim128 (19)	pe1.5_zdim128 (26)	pe0.3_zdim256 (14)	pe1.2_zdim256 (24)
pe1.5_zdim32 (28)	pe0.5_zdim256 (29)	pe2.0_zdim64 (44)	pe1.5_zdim128 (26)
pe1.0_zdim32 (12)	pe0.1_zdim256 (7)	pe2.0_zdim256 (42)	pe0.5_zdim64 (11)
pe0.5_zdim128 (47)	pe1.2_zdim128 (2)	pe1.5_zdim64 (**1**)	pe0.5_zdim256 (29)

2.5.15 18-64

**MI_emb0_top5**	**MI_emb1_top5**	**MI_emb2_top5**	***MP_MIM_top5***
pe1.0_zdim128 (5)	pe1.2_zdim16 (14)	pe1.0_zdim16 (38)	pe1.0_zdim128 (5)
pe1.5_zdim256 (**2**)	pe0.5_zdim32 (7)	pe0.1_zdim10 (46)	pe1.2_zdim16 (14)
pe2.0_zdim256 (6)	pe0.5_zdim256 (18)	pe0.2_zdim3 (15)	pe1.5_zdim256 (**2**)
pe1.0_zdim32 (32)	pe1.2_zdim64 (10)	pe1.2_zdim16 (14)	pe1.0_zdim16 (38)
pe0.5_zdim128 (24)	pe1.5_zdim32 (36)	pe0.3_zdim3 (4)	pe2.0_zdim256 (6)

2.5.16 T4857

**MI_emb0_top5**	**MI_emb1_top5**	***MI_emb2_top5***	**MP_MIM_top5**
pe1.2_zdim128 (33)	pe2.0_zdim256 (23)	pe2.0_zdim256 (23)	pe2.0_zdim256 (23)
pe1.5_zdim256 (24)	pe1.2_zdim256 (19)	pe1.5_zdim256 (24)	pe1.2_zdim128 (33)
pe1.2_zdim256 (19)	pe1.5_zdim256 (24)	pe0.2_zdim256 (48)	pe1.2_zdim256 (19)
pe0.2_zdim16 (41)	pe1.2_zdim128 (33)	pe1.2_zdim128 (33)	pe1.5_zdim256 (24)
pe1.0_zdim256 (42)	pe0.2_zdim256 (48)	pe0.5_zdim3 (**10**)	pe0.2_zdim256 (48)

Supplemental Table S5. Comparison of top 5 RESEPT embeddings for native Moran's I and MP-MIM methods based on the ground truth on the 16 samples. The number in parentheses is the specific rank order in the ground truth ranking. The first three columns are the results of using Moran's I to measure the first dimension (emb0), second dimension (emb1), and third dimension (emb2) of each embedding, respectively.

2.6 Supplemental Table S6

Sample	***MI_emb0***	***MI_emb1***	***MI_emb2***	***GC_emb0***	***GC_emb1***	***GC_emb2***	***MP-MIM***
151507	0.474	*0.811*	0.419	0.415	0.798	0.408	**0.875**
151508	0.626	*0.683*	0.409	0.571	0.655	0.355	**0.741**
151509	*0.618*	0.384	0.540	0.480	0.304	0.457	**0.685**
151510	0.532	*0.654*	0.526	0.485	0.632	0.460	**0.796**
151669	***0.307***	0.087	0.052	0.295	0.072	0.077	0.141
151670	0.012	*0.345*	-0.074	0.032	***0.355***	0.005	-0.062
151671	*0.372*	0.328	0.409	0.335	0.271	0.297	**0.793**
151672	0.461	0.545	0.339	0.388	0.468	0.227	**0.796**
151673	*0.663*	0.328	0.229	0.506	0.269	0.144	**0.819**
151674	*0.535*	0.402	0.378	0.444	0.340	0.297	**0.757**
151675	0.631	*0.654*	0.498	0.606	0.647	0.474	**0.829**
151676	0.550	*0.580*	0.693	0.548	0.567	0.688	**0.728**
2-5	*0.584*	0.396	0.374	0.569	0.333	0.351	**0.592**
2-8	-0.135	-0.168	-0.100	-0.116	-0.156	*-0.076*	**0.069**
18-64	0.344	*0.448*	0.371	0.306	0.381	0.333	**0.590**
T4857	0.062	0.162	***0.244***	0.031	0.105	0.193	0.172

Supplemental Table S6. Comparison between the baseline method using Moran's I or Geary's C on each dimension of RESEPT original embedding and MP-MIM on 16 samples.

2.7 Supplemental Table S7

2.7.1 151507

**MI_emb0_top5**	***MI_emb1_top5***	**MI_emb2_top5**	***MP_MIM_top5***
pca5_res5_l2.43 (24)	pca50_res7_l2.43 (16)	pca5_res0.1_l2.43 (20)	pca50_res7_l2.43 (16)
pca5_res1_l2.43 (21)	pca50_res5_l2.43 (**13**)	pca5_res1_l2.43 (21)	pca50_res5_l2.43 (**13**)
pca5_res0.1_l2.43 (20)	pca50_res1_l2.43 (17)	pca5_res5_l2.43 (24)	pca50_res10_l2.43 (15)
pca5_res3_l2.43 (19)	pca50_res3_l2.43 (14)	pca5_res3_l2.43 (19)	pca50_res3_l2.43 (14)
pca5_res7_l2.43 (23)	pca50_res10_l2.43 (15)	pca5_res7_l2.43 (23)	pca50_res1_l2.43 (17)

2.7.2 151508

**MI_emb0_top5**	***MI_emb1_top5***	**MI_emb2_top5**	***MP_MIM_top5***
pca5_res1_l2.43 (23)	pca5_res3_l2.43 (18)	pca50_res0.1_l2.43 (24)	pca5_res7_l2.43 (19)
pca5_res3_l2.43 (18)	pca5_res7_l2.43 (19)	pca50_res1_l2.43 (**13**)	pca5_res10_l2.43 (21)
pca5_res0.1_l2.43 (22)	pca5_res10_l2.43 (21)	pca50_res3_l2.43 (15)	pca5_res0.1_l2.43 (22)
pca5_res5_l2.43 (20)	pca5_res5_l2.43 (20)	pca50_res5_l2.43 (17)	pca5_res5_l2.43 (20)
pca5_res10_l2.43 (21)	pca5_res0.1_l2.43 (22)	pca50_res7_l2.43 (14)	pca5_res3_l2.43 (18)

2.7.3 151509

**MI_emb0_top5**	***MI_emb1_top5***	**MI_emb2_top5**	***MP_MIM_top5***
pca50_res0.1_l2.43 (24)	pca5_res1_l2.43 (17)	pca50_res0.1_l2.43 (24)	pca5_res10_l2.43 (**13**)
pca50_res1_l2.43 (30)	pca5_res3_l2.43 (14)	pca50_res1_l2.43 (30)	pca5_res1_l2.43 (17)
pca50_res3_l2.43 (22)	pca5_res7_l2.43 (15)	pca50_res5_l2.43 (20)	pca5_res3_l2.43 (14)
pca50_res10_l2.43 (19)	pca5_res10_l2.43 (**13**)	pca50_res10_l2.43 (19)	pca5_res0.1_l2.43 (18)
pca50_res7_l2.43 (21)	pca5_res0.1_l2.43 (18)	pca50_res7_l2.43 (21)	pca5_res7_l2.43 (15)

2.7.4 151510

**MI_emb0_top5**	***MI_emb1_top5***	**MI_emb2_top5**	**MP_MIM_top5**
pca50_res1_l2.43 (18)	pca5_res1_l2.43 (15)	pca50_res0.1_l2.43 (18)	pca50_res0.1_l2.43 (24)
pca50_res0.1_l2.43 (24)	pca5_res3_l2.43 (17)	pca50_res5_l2.43 (22)	pca50_res1_l2.43 (18)
pca50_res5_l2.43 (22)	pca5_res7_l2.43 (13)	pca50_res3_l2.43 (23)	pca50_res5_l2.43 (22)
pca50_res3_l2.43 (23)	pca5_res5_l2.43 (14)	pca50_res0.1_l2.43 (24)	pca50_res7_l2.43 (20)
pca50_res7_l2.43 (20)	pca5_res10_l2.43 (**12**)	pca50_res7_l2.43 (20)	pca50_res10_l2.43 (21)

2.7.5 18-64

MI_emb0_top5	**MI_emb1_top5**	**MI_emb2_top5**	**MP_MIM_top5**
pca5_res3_l2.43 (2)	pca50_res0.1_l2.43 (21)	pca50_res1_l2.43 (24)	pca50_res1_l2.43 (24)
pca5_res5_l2.43 (4)	pca5_res0.1_l2.43 (**1**)	pca50_res3_l2.43 (23)	pca50_res3_l2.43 (23)
pca5_res10_l2.43 (5)	pca50_res1.0_l2.43 (24)	pca50_res5_l2.43 (20)	pca50_res5_l2.43 (20)
pca5_res7_l2.43 (3)	pca5_res1.0_l2.43 (22)	pca50_res10_l2.43 (19)	pca5_res3_l2.43 (2)
pca5_res1_l2.43 (22)	pca5_res3.0_l2.43 (2)	pca50_res0.1_l2.43 (21)	pca5_res10_l2.43 (5)

2.7.6 T4857

**MI_emb0_top5**	**MI_emb1_top5**	**MI_emb2_top5**	**MP_MIM_top5**
pca50_res0.1_l2.43 (36)	pca50_res0.1_l2.43 (36)	pca5_res1_l2.43 (23)	pca50_res0.1_l2.43 (36)
pca50_res5_l2.43 (15)	pca50_res3_l2.43 (16)	pca5_res0.1_l2.43 (19)	pca50_res3_l2.43 (16)
pca50_res10_l2.43 (**13**)	pca50_res1_l2.43 (17)	pca5_res7_l2.43 (20)	pca50_res1_l2.43 (17)
pca50_res3_l2.43 (16)	pca50_res10_l2.43 (**13**)	pca5_res5_l2.43 (21)	pca50_res10_l2.43 (**13**)
pca50_res5_l2.43 (21)	pca50_res7_l2.43 (14)	pca5_res3_l2.43 (22)	pca50_res7_l2.43 (14)

Supplemental Table S7. Comparison of top 5 SpaGCN embeddings for native Moran's I and MP-MIM methods based on the ground truth on six samples. The number in parentheses is the specific rank order in the ground truth ranking. The first three columns are the results of using Moran's I to measure the first dimension (emb0), second dimension (emb1), and third dimension (emb2) of each embedding, respectively.

2.8 Supplemental Table S8

Sample	***MI_emb0***	***MI_emb1***	***MI_emb2***	***GC_emb0***	***GC_emb1***	***GC_emb2***	***MP-MIM***
151507	0.426	0.524	0.481	0.421	0.525	0.482	**0.531**
151508	0.460	0.427	**0.463**	0.450	0.427	**0.463**	0.419
151509	0.381	0.434	0.420	0.387	0.433	0.420	**0.442**
151510	0.439	0.551	0.427	0.439	**0.557**	0.427	0.506
18-64	**0.749**	0.648	0.558	0.748	0.661	0.558	0.660
T4857	0.407	0.411	0.356	0.405	0.411	0.356	**0.414**

Supplemental Table S8. Comparison between the baseline method using Moran's I or Geary's C on each dimension of the SpaGCN original embedding and MP-MIM on six samples.
